# Spatial Regression Models for Field Trials: A Comparative Study and New Ideas

**DOI:** 10.3389/fpls.2022.858711

**Published:** 2022-03-30

**Authors:** Stijn Hawinkel, Sam De Meyer, Steven Maere

**Affiliations:** ^1^Department of Plant Biotechnology and Bioinformatics, Ghent University, Ghent, Belgium; ^2^VIB Center for Plant Systems Biology, Ghent, Belgium

**Keywords:** prediction, spatial autocorrelation, field trial, generalized least squares, cross-validation, feature selection, simulation

## Abstract

Naturally occurring variability within a study region harbors valuable information on relationships between biological variables. Yet, spatial patterns within these study areas, e.g., in field trials, violate the assumption of independence of observations, setting particular challenges in terms of hypothesis testing, parameter estimation, feature selection, and model evaluation. We evaluate a number of spatial regression methods in a simulation study, including more realistic spatial effects than employed so far. Based on our results, we recommend generalized least squares (GLS) estimation for experimental as well as for observational setups and demonstrate how it can be incorporated into popular regression models for high-dimensional data such as regularized least squares. This new method is available in the BioConductor R-package *pengls*. Inclusion of a spatial error structure improves parameter estimation and predictive model performance in low-dimensional settings and also improves feature selection in high-dimensional settings by reducing “red-shift”: the preferential selection of features with spatial structure. In addition, we argue that the absence of spatial autocorrelation (SAC) in the model residuals should not be taken as a sign of a good fit, since it may result from overfitting the spatial trend. Finally, we confirm our findings in a case study on the prediction of winter wheat yield based on multispectral measurements.

## 1. Introduction

Measurements on a cohesive study area, such as performed in field trials (Singh et al., 2003), ecological surveys (Liebhold, 2002), or remote sensing (Wójtowicz et al., 2016), provide a wealth of knowledge on variability occurring between living organisms. On the one hand, the common origin of the data and the use of the same cultivar, soil, weather, and cultivating conditions reduce the variability to manageable levels. On the other hand, there often remains sufficient variability that can be linked to other data types gathered on the same area, such as genomic or optic measurements of the vegetation or environmental features. In observational studies, naturally occurring variability in the outcome variable is linked to naturally occurring variability in the explanatory variables (Bernal-Vasquez et al., 2014; Barmeier and Schmidhalter, 2016; Wójtowicz et al., 2016; Yue et al., 2017; Rocha et al., 2018, 2019; Cruz et al., 2020; Fu et al., 2020; Harkel et al., 2020; Ploton et al., 2020; Tang et al., 2021; Yoosefzadeh-Najafabadi et al., 2021; Zhou et al., 2021). Alternatively, in experimental (interventional) studies, researchers may actively apply a treatment to part of the study area and investigate the link between this treatment and the variability in the field (Lado et al., 2013; Elias et al., 2018; Mao et al., 2020). For both cases (observational or experimental studies), links between the variables under study can then be tested through formal hypothesis tests and exploited to build, e.g., prediction models for phenotypic traits based on easily measurable variables.

Observations of a given variable across the study area are often not independent but instead exhibit spatial patterns. These patterns may take the shape of small clusters of similar observations, but also of linear gradients or edge effects (despite the use of buffer rows at the border) (Austin and Blackwell, 1980; Langton, 1990; Romani et al., 1993; Haase, 1995; Barmeier and Schmidhalter, 2016; Cruz et al., 2020; Zhou et al., 2021). All of these spatial patterns introduce a dependence between nearby observations, known as spatial autocorrelation (SAC), which violates the assumption of independence among observations of a single variable underlying many statistical methods (Cressie, 1993; Diggle et al., 1998). Confined to a spatial domain, variables that are independent may exhibit an apparent association when they are both spatially autocorrelated, which is known as “spatial confounding” (Hodges and Reich, 2010; Paciorek, 2010; Nobre et al., 2020). As a result, SAC can inflate the type I error rate (Dormann et al., 2007; Kissling and Carl, 2008; Beale et al., 2010), can increase the bias and variability of parameter estimators, and decrease predictive model performance (Dormann et al., 2007; Beale et al., 2010; Rocha et al., 2018, 2019), and can unduly favor the selection of features with spatial structure in feature selection procedures or hypothesis tests (known as “red-shift”) (Lennon, 2000; Bini et al., 2009; Meyer et al., 2019; Harisena et al., 2021). Another consequence of SAC concerns data splitting procedures such as cross-validation (CV), whereby the available dataset is repeatedly split into a training and a test set to get an estimate of model performance on new data. The SAC between observations may cause overoptimistic assessment of model performance, as training and test sets are not truly independent (Brenning, 2005; Pohjankukka et al., 2017; Roberts et al., 2017; Meyer et al., 2019; Schratz et al., 2019; Ploton et al., 2020).

A large body of theory is dedicated to spatial interpolation, i.e., leveraging from SAC to improve prediction on new data points lying in between past observations (e.g., kriging) (Cressie, 1993; Diggle et al., 1998). Yet in many contexts (e.g., field trials, ecology), models are trained for application on new datasets gathered in another setup. Spatial coordinates cannot be included in the prediction model in this case, and SAC is a mere nuisance: one wants to learn the relationship between outcome and regressors regardless of spatial effects. Spatial regression models developed for this purpose account for spatial structure in either the mean or error term, but ignore this term when making predictions on new data. A selection of these methods is included and discussed here, but many more exist (see Beale et al., 2010 for a more exhaustive enumeration). Generalized least squares (GLS) is a generalization of ordinary least squares (OLS) to the case of dependent errors, which can be applied for instance to spatial data. Since it requires the estimation of a large number of covariances between observations, additional assumptions are needed to render the estimation feasible. For the case of spatial data, some functional relationship with a low number of parameters is assumed, which describes the decay of covariance with distance. The corresponding parameters are then estimated from the data (Pinheiro and Bates, 2000). The integrated nested Laplace approximation (INLA) method is based on a similar error model, but performs parameter estimation in a Bayesian context (Selle et al., 2019). Alternatively, neighboring observations can be included as nuisance terms in either the mean or the error model in simultaneous autoregressive models. These models require the definition of a neighborhood based on distance or a certain number of closest neighbors (Kissling and Carl, 2008; Selle et al., 2019). Spatial filtering takes a different approach by performing eigendecomposition on the proximity matrix of the observations and including eigenvectors associated with the outcome variable in the mean model (Tiefelsdorf and Griffith, 2007; Murakami and Griffith, 2015). The spatial structure of the outcome variable can also be modeled more explicitly by including a smooth term in the mean model that is a function of location, e.g., a spline in a generalized additive model (GAM) (Wood, 2003; Rodríguez-Álvarez et al., 2018).

Several simulation studies have investigated how well these methods deal with spatial data. One study to compare a wide range of them was Beale et al. (2010), who found a poor performance for OLS and spatial filtering with forward selection in presence of SAC, and better performance for GAM, GLS, and simultaneous autoregressive models. Kissling and Carl (2008) found simultaneous autoregressive models with spatial terms in the errors to outperform OLS, and Ludwig et al. (2020) found GAM to perform better than OLS in the presence of spatial confounding. Murakami and Griffith (2015) found a better performance of spatial filtering with random effects than with forward selection. The data generation models used in these simulation studies were invariably based on the general assumption of SAC decreasing with the distance between points (Wang and Zhu, 2009; Beale et al., 2010; Alesso et al., 2019; Rocha et al., 2019, 2021; Ludwig et al., 2020; Mao et al., 2020; Harisena et al., 2021). Even when this assumption is acceptable for analysis purposes, this way of drawing observations is not ideally suited to simulate structured spatial patterns observed in some real studies, such as linear gradients or edge effects (Austin and Blackwell, 1980; Romani et al., 1993; Barmeier and Schmidhalter, 2016; Cruz et al., 2020; Zhou et al., 2021).

In field trials, experiments are often designed in anticipation of spatial factors confounding the outcome, e.g., by choosing block designs perpendicular to an expected gradient (Verdooren, 2020). In addition, at the analysis stage, the inclusion of spatial effects in regression models has been shown to improve predictive model performance on real data (Lado et al., 2013; Elias et al., 2018; Mao et al., 2020) and in simulations (Alesso et al., 2019; Rocha et al., 2019; Mao et al., 2020; Harisena et al., 2021), although no improvement was found in another study (Bernal-Vasquez et al., 2014). However, corrections are often done only according to discrete factors of the design, e.g., by adding a correction factor for the rows and columns of checkerboard designs. Yet, the outcome variable may exhibit SAC that is unpredictable at the design stage, within and across subplots. In ecological surveys or when monitoring real cultured fields, there may even be no fixed design factors at all. Spatial dependence between individual plants or parts of fields then needs to be accounted for in the data analysis rather than in the experimental design. An example is remote sensing of cultured fields through unmanned aerial vehicles (UAVs, colloquially known as drones). The idea is to predict a phenotype such as a yield or biomass based on reflectance measurements. Often vegetation indices are extracted from the raw images as ratios of spectral band intensities to serve as predictors. Although imaging and phenotyping data on a single field are often spatially autocorrelated, this spatial structure is often not taken into account in the data analysis (Yue et al., 2017; Zhang et al., 2019; Fu et al., 2020; Harkel et al., 2020; Lee et al., 2020; Tang et al., 2021; Yoosefzadeh-Najafabadi et al., 2021).

Contemporary genomic or hyperspectral measurements can easily yield many more variables than the number of different plants or plots that were phenotyped. In this high-dimensional context, penalized (or regularized) linear models such as the least absolute shrinkage and selection operator (LASSO) are often employed for feature selection and building predictive models. The question then arises how spatial structure can be incorporated into these models as well. Recently, Cai et al. (2019) combined the LASSO with a moment estimator for autocorrelation in the context of spatial data. Yoon et al. (2013) combined penalized regression with autoregressive modeling. The combination of the GLS framework with the penalized regression methods has also been proposed before, but mostly in other contexts than spatial modeling (Shijun and Benzao, 2006; Yang and Yuan, 2019; Mylona and Goos, 2021). Seya et al. (2015) applied the LASSO to eigenvector selection in eigenvector spatial filtering, and Fan et al. (2017) combined ridge regression with spatial filtering to find that inclusion of spatial effects improves parameter estimation. Yet, Wang and Zhu (2009) found a trade-off between predictive model performance and accuracy of the feature selection, with methods giving better predictions having worse feature selection performance. Software implementations are available for some of these methods (Fan et al., 2017; Yang and Yuan, 2019), but the solutions proposed are usually restricted to a small class of problems.

In view of the multitude of experimental designs, analysis goals, potential spatial patterns, and methods to account for them, deciding on an analysis strategy is not a trivial task. Therefore, we present here a simulation study to assess how various established methods to account for SAC effects in regression models perform (in terms of controlling red-shift, estimating model parameters, selecting relevant features and quantitatively predicting the outcome variable) when confronted with different forms of SAC observed in field or greenhouse trials. We consider experimental studies as well as observational studies and include more realistic spatial structures than in other simulation studies (Dormann et al., 2007; Beale et al., 2010; Murakami and Griffith, 2015; Harisena et al., 2021), such as linear gradients and edge effects. We also combined GLS, which accounts for spatial structure, with penalized regression for high-dimensional data, and investigate how this affects model performance and feature selection. This new method is available as the R-package *pengls* from BioConductor at https://bioconductor.org/packages/pengls/. Model performance was estimated using random and blocked CV paradigms and compared with performance on independently simulated data. Furthermore, we investigated the role of residual SAC in assessing model fit. To illustrate our findings, we apply spatial and non-spatial methods to a real dataset from remote sensing of wheat fields.

## 2. Materials and Methods

### 2.1. Simulation Study

#### 2.1.1. Data Generation

Observations are simulated at locations sampled without replacement and with equal probability from an equispaced 15 x 15 grid with interpoint distance 1, indexed by an integer coordinate pair (*x, y*). Outcome variable A and regressors *B*_*j*_ with j = 1,..,p are generated for samples i=1,…,n at locations (*x*_*i*_, *y*_*i*_) as


(1) $$\begin{array}{l} {\text{B}}_{j}\sim MVN\left(f_{bj}(\text{x},\text{y}),\Sigma_{bj}(\text{x},\text{y})\right)\\ \text{A}\sim MVN\left(f_{a}(\text{x},\text{y})+\text{B}\beta ,\Sigma_{a}(\text{x},\text{y})\right) \end{array}$$

MVN indicates the multivariate normal distribution, *f*_*a*_ and *f*_*b*_ are continuous smooth functions that describe the evolution of the expected values with the location. The **Σ** matrices describe the covariance structure between the observations as a function of space, whereby the strength of the correlation decreases with the distance between the observations. Hence, specifying a spatial smooth function or a spatial covariance structure are two different ways of engendering spatial structure in the data. All regressors **B**_*j*_ are drawn independently. Data are drawn for the following scenarios:

1. “None”: no spatial structure.
  - *f*(*x, y*) = 0
  - $\Sigma =\sigma^{2}{\text{I}}_{n}$ with *I*_*n*_ the identity matrix of size *n* and σ^2^ = 1 the residual variance.
2. “Linear”: a linear change in baseline.
  - *f*(*x, y*) = (*x, y*)^*t*^**γ** with **γ** a two-dimensional gradient with components drawn uniformly on [−0.5, 0.5] and independently.
  -
$\Sigma =\sigma^{2}{\text{I}}_{n}$

3. “Edge”: observations along the edge have a different mean.
  - $f\left(x,y\right)=\frac{\beta_{edge}}{d_{edge}\left[\left(x,y\right)\right]}$, with *d*_*edge*_[(*x, y*)] the distance to the closest edge of the field (assumed to be one unit distance away from the border rows) and β_*edge*_ drawn for each realization from a normal distribution with mean 0 and variance 50.
  -
$\Sigma =\sigma^{2}{\text{I}}_{n}$

4. “gaussCor”: correlation between observations decays as a bell curve.
  - *f*(*x, y*) = 0
  - $\Sigma =\sigma^{2}\left(\tau {\text{I}}_{n}+\left(1-\tau \right)\Sigma^{gauss}\right)$ with $\Sigma_{ik}^{gauss}=\exp\left(-{\left(d\left[\left(x_{i},y_{i}\right),\left(x_{k},y_{k}\right)\right]/r\right)}^{2}\right)$ and *d* the Euclidean distance between two points. *r* = 7.5 is called the range parameter and τ = 0.25 is the nugget parameter.

**Figure 1:**
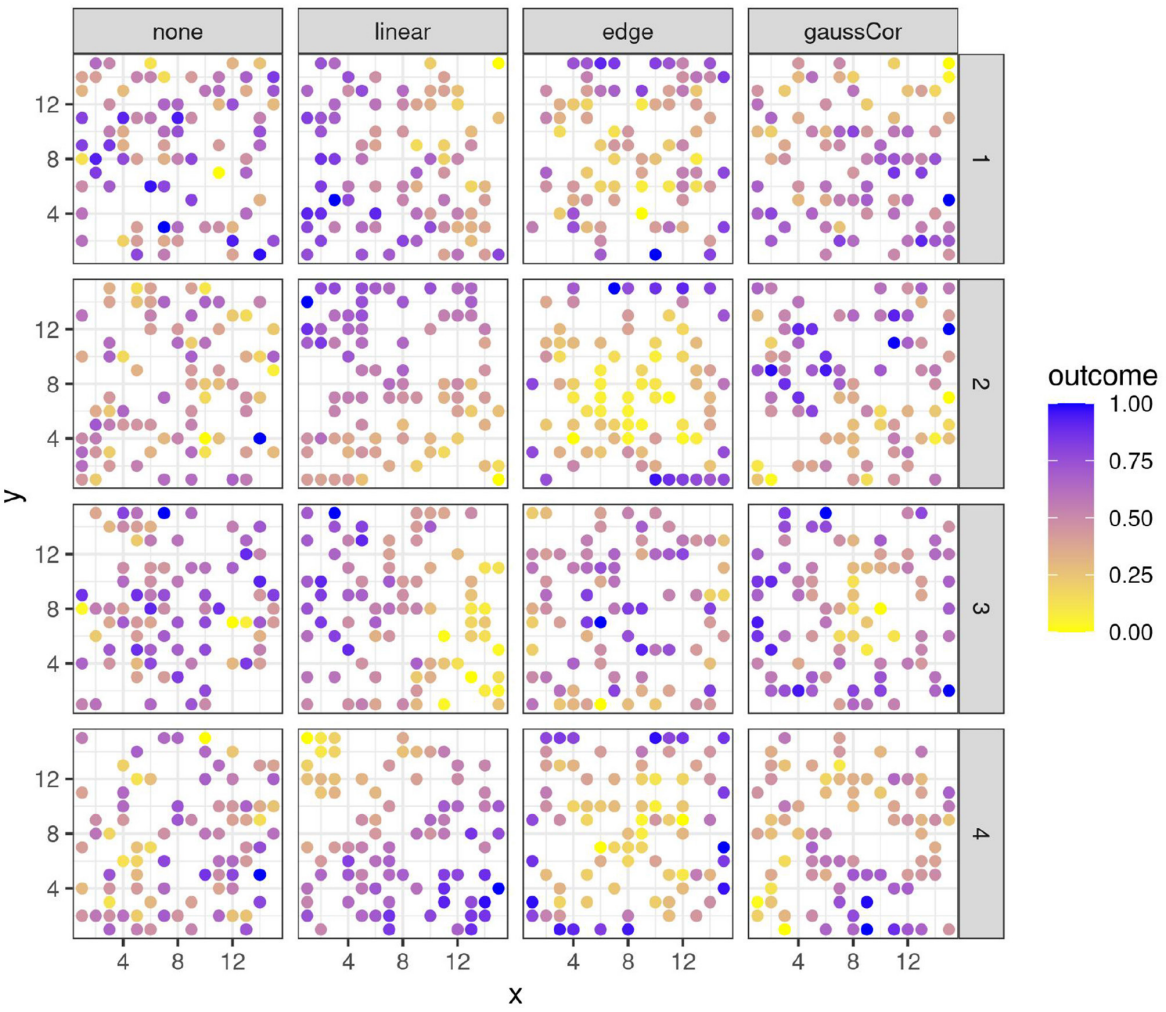
Four instances (rows) of variables with different spatial structures (columns). x- and y-axes represent spatial coordinates. Colors reflect the value of the outcome variable, scaled to the 0-1 range for legibility.

The parameter values were chosen to qualitatively emulate the spatial patterns observed in real data (Austin and Blackwell, 1980; Langton, 1990; Romani et al., 1993; Haase, 1995; Barmeier and Schmidhalter, 2016; Cruz et al., 2020; Zhou et al., 2021). Some examples of variables generated in these ways are shown in Figure 1. All combinations of spatial structures of outcome and regressors were made, whereby all regressors with spatial structure follow the same type of spatial structure per scenario but are otherwise independent. Note that the edge and linear spatial effects increase the variance of a variable, whereas gaussCor decreases its variance (refer to Supplementary Figure S1). The number of features was varied between *p* = 1 (univariate scenario), *p* = 50 (low-dimensional scenario), and *p* = 100, 200, or 300 (high-dimensional scenario). In the univariate scenario, the design is either observational, with a continuous regressor following a spatial pattern, or experimental, whereby a binary regressor (e.g., treatment vs. control) has a checkerboard pattern across the field. In this latter scenario, an equispaced 18x18 grid was used to allow for partitioning into square subplots with sizes 9, 6, and 3 (refer to Supplementary Figures S2–S4), and the 4 different spatial structures are introduced in outcome A only (in the same way as for the 15 x 15 grid). For the training datasets, the sample size was *n* = 100 for the univariate and low-dimensional scenarios and 50 for the high-dimensional scenario. Test datasets were also generated (see below), with the sample size equal to 10 in all cases (the same size as the left-out folds, refer to Section 2.1.3 below). In the multivariate scenarios, 50% of the regressors were generated with spatial structure, the others without. In both groups of regressors, 20% of the components of ***β*** were drawn from a zero-mean normal distribution with standard deviations varying between 0.5, 1, and 2 in the low-dimensional scenario and between 0.25 and 0.5 for the high-dimensional scenario, the other components were zero. This setting leads to comparable true *R*^2^ values between low- and high-dimensional scenarios (refer to Supplementary Figure S5). For the univariate scenarios, a null setting with β = 0 was included, as well as an alternative setting whereby β was drawn from a normal distribution with mean 0 and variance 0.25. In the univariate and low-dimensional scenarios, 1,000 Monte Carlo instances were generated, in the high-dimensional scenarios 200. For the INLA models, the number of Monte Carlo instances was limited to 100 in the low- and high-dimensional scenarios for computational reasons.

In a small side simulation of the low-dimensional case, we investigate the role of the number of features *p*. While fixing the standard deviation of the components of ***β*** at 1, we let the number of features vary between 10, 20, and 50, with a sample size of *n* = 100.

Even when β_*j*_ = 0, some combinations of the spatial configuration of regressor and outcome lead to non-zero true correlations between their means, for instance, in the case when both experience an edge effect in the observational scenario (refer to Supplementary Figure S6), or between edge effect in the outcome and the checkerboard design in the experimental scenario (refer to Supplementary Figure S7). This phenomenon is known as spatial confounding. Strictly statistically speaking, this is then not a null scenario, and excess small *p*-values cannot be seen as a shortcoming of the regression method. Yet, when confined to the two-dimensional space of a field, two variables that are independent can easily exhibit spatial patterns that render them correlated, e.g., two linear gradients in a field are most likely not orthogonal. Therefore, ideally, we would prefer a method that is robust to these kinds of confounded spatial patterns, and we will consider every discovery that results only from this spatial confounding as a false one.

#### 2.1.2. Regression Models

All analyses were performed using the R programming language, version 4.0.3 (R Core Team, 2020). Details on the package versions can be found in Supplementary Section 5.

##### 2.1.2.1. Low-Dimensional Scenario

Ordinary least squares was fitted using the *lm* function in the *stats* package. GLS with Gaussian covariance decay assumed was fitted using the *gls* and *corGaus* functions in the *nlme* R-package (Pinheiro et al., 2021). This function iterates between estimating the spatial covariance structure of the residuals ($\hat{\Sigma }$) and fitting an OLS model on **A** and **B** matrices premultiplied by the inverse square root factor of this covariance structure to estimate ***β***, according to the following model:


(2) $$\begin{array}{r} {\hat{\Sigma }}^{-1/2}\text{A}={\hat{\Sigma }}^{-1/2}\text{B}\beta +\epsilon \end{array}$$

with ϵ~*N*(0, σ^2^) i.i.d. GAM with 2D thin plate spline with 5 degrees of freedom were fitted using the *gam* function in the *mgcv* package (Wood, 2003). Spatial filtering with the forward selection of spatial eigenvectors or with random effects on the spatial eigenvectors is done with the *esf* and *resf* functions from the *spmoran* package, respectively (Murakami, 2021). Simultaneous autoregressive models were fitted using the *errorsarlm* function in the *spatialreg* package (Bivand and Piras, 2015), using the following two definitions of adjacency. Neighborhoods were defined as either the 8 nearest neighbors (“nearest neighbors”), or all other observations lying within one-fifth of the maximum distance between any two observations (“distance”). In the univariate scenario, we used both versions, for the other analyses, we used only the nearest neighbor version as this one performed best in the univariate models. INLA was applied using the AR(1) model as well as the Matérn model as described by Selle et al. (2019). We used INLA's posterior mean of the β parameter as a point estimate for making predictions and for evaluating parameter estimation and considered the null hypothesis β = 0 to be rejected when 0 was outside the 95% credible interval. In the univariate scenario, we used both INLA versions, for the other analyses, we used only the Matérn version as this one performed best in the univariate models. When using these methods for making predictions on new datasets, spatial components are omitted from the models, e.g., the thin plate spline for GAM, the spatial eigenvectors for spatial filtering, the neighboring observations for a simultaneous autoregressive model, or the spatial error term for the GLS and INLA Matérn model. These components are only included to obtain a better estimate of ***β*** but are not included for extrapolation. No feature selection was performed in the low-dimensional scenario; all regressors are included in the models. In the univariate scenario with a checkerboard design, the data are analyzed with and without accounting for row and column effects. Accounting for these effects is done by adding dummy variables for rows and columns (excluding a reference row and column) to the regressor matrix **B**. Note also that in the low-dimensional scenario, no parameter tuning is required, so the parameter estimates are not affected by the CV (refer to Section 2.1.3).

##### 2.1.2.2. High-Dimensional Scenario

For the high-dimensional datasets, a number of variants of the elastic net (EN) implemented by the *glmnet* function in the *glmnet* package are used (Friedman et al., 2010). The mixing parameter α is fixed at 0.5. EN performs feature selection by shrinking some of the coefficients in ***β*** to zero. We fit the following models:

- The regular EN, without spatial correction.
- A GLS version of EN, which iterates until convergence between
  1. Fitting the EN on the data matrix pre-multiplied by the square root inverse covariance matrix ${\hat{\Sigma }}^{-1/2}$ as in (2), which estimates ***β***.
  2. Estimating spatial structure of the residuals **Σ** of this model through restricted maximum likelihood using the *gls* function in the *nlme* package, assuming Gaussian covariance decay.

As starting value, the identity matrix is used for **Σ**. This iterative procedure minimizes the following criterion (Friedman et al., 2010)


(3) $$\begin{array}{r} {\left(\text{A}-\beta_{0}-\text{B}\hat{\beta }\right)}^{t}{\hat{\Sigma }}^{-1}\left(\text{A}-\beta_{0}-\text{B}\hat{\beta }\right)+0.5\lambda ||\hat{\beta }|{|}_{1}+0.25\lambda ||\hat{\beta }|{|}_{2}^{2}, \end{array}$$

where λ is a tuning parameter, β_0_ is the intercept, and ***β*** is the parameter vector of interest, **Σ** is a nuisance parameter, and $\left||\hat{\beta }|{|}_{1}=\overset{p}{\underset{j=1}{\sum }}|\beta_{j}\right|$ and $||\hat{\beta }|{|}_{2}^{2}=\overset{p}{\underset{j=1}{\sum }}\beta_{j}^{2}$ the *L*_1_ and squared *L*_2_ norms of the ***β*** parameter vector. Convergence is assumed when the mean squared change in the predictions between two iterations drops below 0.00025.

- An EN model with spatial eigenvectors as regressors in addition to the regular **B** regressors. The spatial eigenvectors are found using the *meigen* function in the *spmoran* package (Murakami, 2021).
- A GAM-LASSO model is available in the *plsmselect* package (Ghosal and Kormaksson, 2019). We adapted it to allow the value of the mixing parameter α to be set by the user, which allows for EN to be fitted.
  In addition, an INLA Matérn model as in the low-dimensional scenario is fitted. Unlike the previous models, this model is not based on elastic net and does not perform feature selection.

Cross-validation (either blocked or random, see below) is used to tune the penalty parameter λ in the elastic net models, choosing the λ one standard error above the λ with the highest CV *R*^2^, as is the default in the *glmnet* package. Note that since λ is tuned based on different CV paradigms, the test performance on independent test sets may now depend on the paradigm, unlike in the low-dimensional scenario.

#### 2.1.3. Diagnostics

In the univariate scenario, the type I error rate was calculated for the null scenario, and the power to call the association between A and B significant and the mean squared error (MSE) of $\hat{\beta }$ for the alternative scenario. The significance level was set at 5%. For the low and high-dimensional scenarios, methods are evaluated for predictive performance by *R*^2^, which measures the proportion of variance in A explained by the regressors **B**:


(4) $$\begin{array}{r} R^{2}=\frac{Var\left(A\right)-Var\left(A|\text{B}\right)}{Var\left(A\right)}. \end{array}$$

Generally, the regressors **B** are expected to explain only part of the variance in A, yielding *R*^2^ values between 0 and 1. The *R*^2^ is estimated as ${\hat{R}}^{2}=1-\frac{\overset{n}{\underset{i=1}{\sum }}{\left(a_{i}-{â}_{i}\right)}^{2}}{\overset{n}{\underset{i=1}{\sum }}{\left(a_{i}-ā\right)}^{2}}$, with â_*i*_ the predicted outcome for observation *i* and ā the average outcome. When the prediction model is poor, the estimates â_*i*_ may be such that they increase the variance, leading to negative values of the ${\hat{R}}^{2}$ measure. For simplicity, ${\hat{R}}^{2}$ is designated by *R*^2^ in the remainder of the document. *R*^2^ was estimated using independent test sets (see below), but also using CV.

It is known that SAC can cause an upward bias in model evaluation by CV and that this effect can be mitigated by choosing the folds as clusters of nearby observations rather than randomly over the field (refer to Supplementary Figure S8 for an illustration). This is called blocked or spatial CV and reduces the SAC between the folds (Brenning, 2005; Pohjankukka et al., 2017; Roberts et al., 2017; Meyer et al., 2019; Schratz et al., 2019; Ploton et al., 2020). When CV is used to tune hyperparameters, this blocked CV might also lead to better models by discouraging overfitting, although this could not be demonstrated so far (Schratz et al., 2019). We applied random CV, whereby observations are assigned to folds randomly, and blocked CV, whereby folds are formed by clustering nearby points using the *kmeans* function from the *stats* package. The number of folds was 10 for the low-dimensional and 5 for the high-dimensional scenario, in each case leading to fold sizes of 10. Predictions are mean-centered before evaluating *R*^2^ on the test datasets and in the CV, since we are mainly interested in relative differences within fields rather than in accurate prediction of the baseline per field. Three different *R*^2^ measures were calculated per simulated dataset:

- Cross-validation *R*^2^ (cv*R*^2^), the average of the *R*^2^ values calculated on the left-out folds.
- *R*^2^ on a test dataset of size n=10 (test*R*^2^spatial), generated with the same relation ***β*** between A and **B**, and the same spatial structure as the training dataset in regressors and outcome. This means that if, for instance, the spatial structure was linear in the original training dataset, it will also be such in the test dataset, but a new gradient vector ***γ*** is drawn. Its *R*^2^ then reflects prediction performance on all points of a single, similar plot. A 5 x 5 grid was used to obtain similar spacing between the observations as in the training set.
- *R*^2^ on a test dataset of size *n* = 10 (test*R*^2^none), generated with the same relation ***β*** between A and **B**, but without any spatial structure in outcome or regressor. This *R*^2^ reflects the performance of the model over all possible plants from different fields, so without SAC between them.

For evaluation on the test dataset, a model is trained on the training dataset after omitting one of the folds, using the hyperparameter λ optimized in the CV on the entire dataset, such that the size of the training dataset is the same for evaluation on an external test dataset and for CV. Only one test dataset is generated per training set, as the outer Monte Carlo loop of 1,000 instances will average out the test performance over as many test datasets. When the model fitting procedure failed, all *R*^2^ values were set to 0. As a second diagnostic, the MSE of the estimator of ***β*** is calculated. For the high-dimensional scenario, we also consider the feature selection efficiency. We define *TP* as the number of truly predictive features selected, *FP* as the number of non-predictive features selected and *FN* as the number of truly predictive features not selected, we calculate the sensitivity as $\frac{TP}{TP+FN}$ and true discovery proportion (TDP) as $\frac{TP}{TP+FP}$. We also calculate the proportion of features with spatial patterns among the discoveries, which should be 50% on average if the method does not preferentially select spatial features.

Spatial autocorrelation can be quantified through the Moran's I statistic (Moran, 1950). It can be calculated on the raw outcome A (marginal Moran's I or *I*_*m*_) or on the residuals from regressing A on **B** (Moran's I conditional on B or *I*_*c*_). These measures are defined as:


(5) $$\begin{array}{l} I_{m}=\frac{n}{s_{w}}\frac{\sum_{i=1}^{n}\sum_{j=1}^{n}w_{ij}(a_{i}-\bar{a})(a_{j}-\bar{a})}{\sum_{i=1}^{n}{\left(a_{i}-\bar{a}\right)}^{2}}\\ I_{c}=\frac{n}{s_{w}}\frac{\sum_{i=1}^{n}\sum_{j=1}^{n}w_{ij}(a_{i}-{\hat{a}}_{i})(a_{j}-{\hat{a}}_{j})}{\sum_{i=1}^{n}{\left(a_{i}-\hat{a}\right)}^{2}}, \end{array}$$

with $s_{w}=\overset{n}{\underset{i=1}{\sum }}\overset{n}{\underset{j=1}{\sum }}w_{ij}$, ā denotes the average outcome and â_*i*_ denotes the outcome predicted by the regression model. The matrix **W** with entries *w*_*ij*_ is a weight matrix with zeroes on the diagonal and off-diagonal entries usually decreasing with the distance between observations *i* and *j*. Note that the *I*_*c*_ in our case will also capture spatial autocovariance that is included in model components other than the regressors since â_*i*_ is calculated after omitting all spatial terms, i.e., only $\hat{\beta }$ is used for making predictions. The value of the Moran's I statistic strongly depends on the choice of the weight matrix. Here, we set its off-diagonal elements equal to the reciprocal of the euclidean distance between the points, although this choice is not ideal for detecting edge effects (refer to Supplementary Figure S9).

The Moran's I statistic relates the total covariance of each observation with itself $1/n\overset{n}{\underset{i=1}{\sum }}{\left(a_{i}-ā\right)}^{2}$, i.e., the variance (Cov(A, A)=Var(A)), to the short-range weighted spatial autocovariance with nearby observations $\frac{\overset{n}{\underset{i=1}{\sum }}\overset{n}{\underset{j=1}{\sum }}w_{ij}\left(a_{i}-{â}_{i}\right)\left(a_{j}-{â}_{j}\right)}{\overset{n}{\underset{i=1}{\sum }}\overset{n}{\underset{j=1}{\sum }}w_{ij}}$. Since the weighted spatial autocovariance is always smaller than or equal to the variance, Moran's I statistics are product-moment correlations that lie between –1 and 1 (Day et al., 2004).

The difference between *I*_*m*_ and *I*_*c*_ indicates how the model affects unexplained autocovariance, and could thus be used as a measure of model performance. Yet, because of its definition as a ratio, the Moran's I statistic can be difficult to interpret, as a single, directional change in Moran's I may result from several possible changes in variance and/or short range autocorrelation. As for *R*^2^, *I*_*c*_ was estimated on the test and training residuals, and on the residuals of the left-out folds in the CV. Yet, their comparison is complicated by the fact that the expected value of the Moran's I statistic under the null hypothesis depends on the sample size *n*: it equals $E\left(I\right)=\frac{-1}{n-1}$. It will thus automatically differ when calculated on entire training datasets or smaller left-out folds. As a solution, we subtract its expected value from every Moran's I statistic.

### 2.2. Case Study

As a case study, we include an investigation on prediction of wheat grain yield and protein content based on Unmanned Areal Vehicle (UAV) spectral reflectance measurements from real farming fields (Zhou et al., 2021). Four fields were measured in two different years (Fields 1 and 2 in 2018 and Fields 3 and 4 in 2019, with 90 and 101, and 68 and 68 samples, respectively) for the same crop (winter wheat) and cultivating conditions. For the first two fields, plant samples were collected along 3 lateral and one longitudinal transects in the field. For the other two fields, plant samples were collected from 10 m × 15 m grid points across each field (refer to Supplementary Figure S10) and measured for yield and protein content (Supplementary Figure S11). All fields were also surveyed with a camera mounted on a UAV. As usual, to eliminate confounding factors such as atmospheric effects and sun angle, features in the form of vegetation indices and spectral ratios were extracted from the resulting images prior to model fitting. Because of high multicollinearity between these indices (refer to Supplementary Figures S12–S16), for each field, only the set of most important principal components that together explain at least 99% of the variance are included as regressors (3 components for fields 1–3 and 4 components for field 4), refer to Supplementary Figure S17. Low-dimensional prediction models as introduced in Section 2.1.2.1 are trained on all fields separately, and predictive performance is estimated using 5-fold CV. The CV was repeated 20 times and resulting cv*R*^2^s were averaged. Next, the trained models were validated by predicting yield and protein content for other fields, mean-centering these predictions, and comparing them to mean centered observed values of other fields. For validating the models on other fields, we used the entire training dataset to fit the model, rather than leaving out 1 fold as in the simulation study.

## 3. Results

The performance of different spatial regression methods was investigated in simulations for two univariate scenarios and for multivariate low- and high-dimensional scenarios. Next, the results were compared to the performance of the methods on a field trial dataset on winter wheat (Zhou et al., 2021).

### 3.1. Univariate Scenario

**Figure 2:**
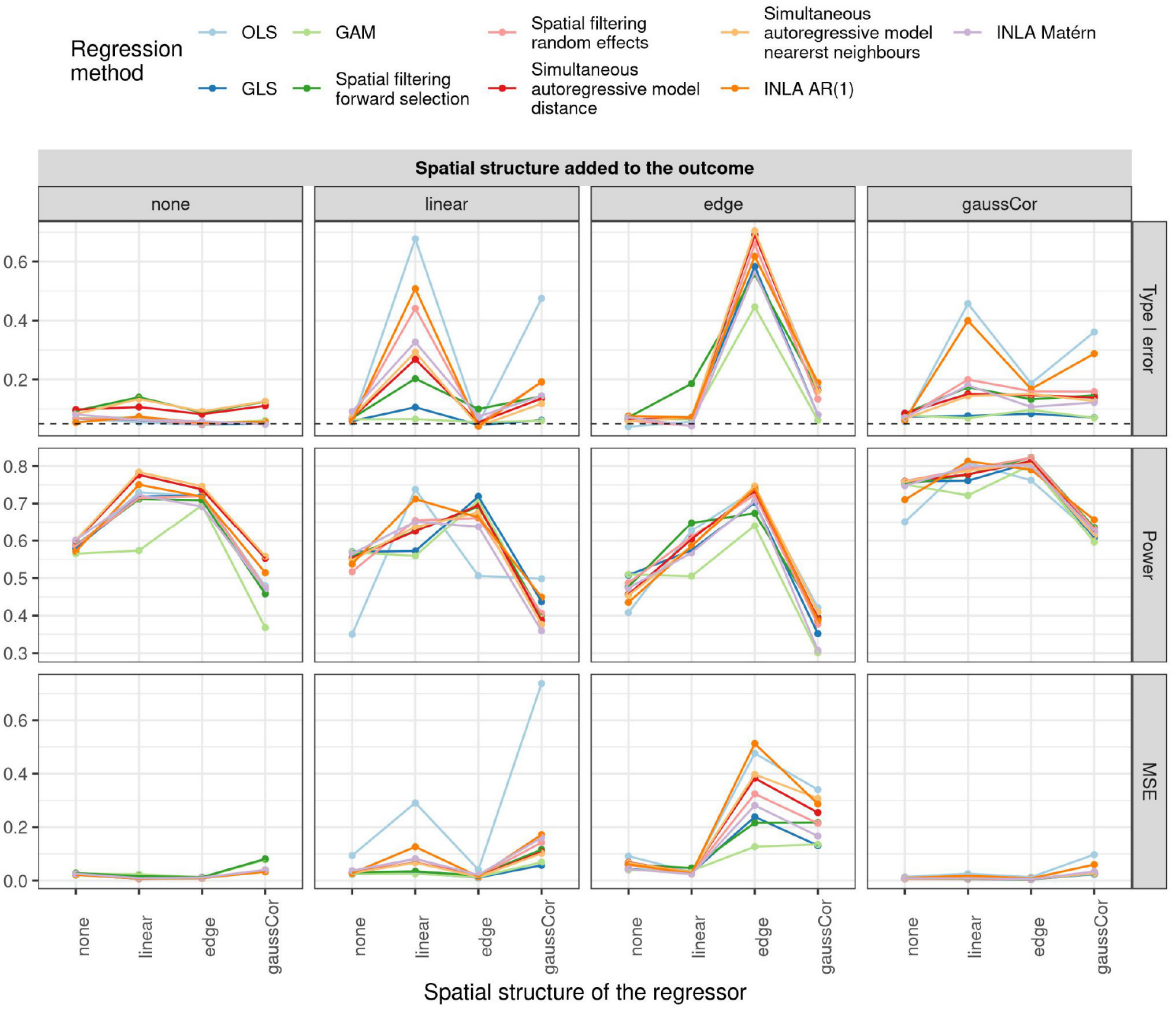
Type I error (top), power (middle), and MSE of $\hat{\beta }$ (bottom) for the univariate observational study scenario for different regression methods (colors) and different spatial structures of the outcome (columns) and regressor (x-axis). The dashed horizontal line indicates the 5% significance level.

The type I error, power, and MSE of $\hat{\beta }$ for the observational study scenario are shown in Figure 2. OLS, spatial filtering, INLA Matérn, and the autoregressive models (simultaneous and INLA) generally struggle the most to control the type I error in presence of spatial effects. On the other hand, GLS and GAM are most robust to spatial confounding, as they generally control the type I error rate at around 5–10%. However, when both outcome and regressor experience an edge effect, no method manages to detect the spatial confounding and all methods call the association significant. Also, combinations of linear and/or Gaussian SAC patterns in the regressor and outcome generally produce high type I error rates, except when using GLS or GAM.

**Figure 3:**
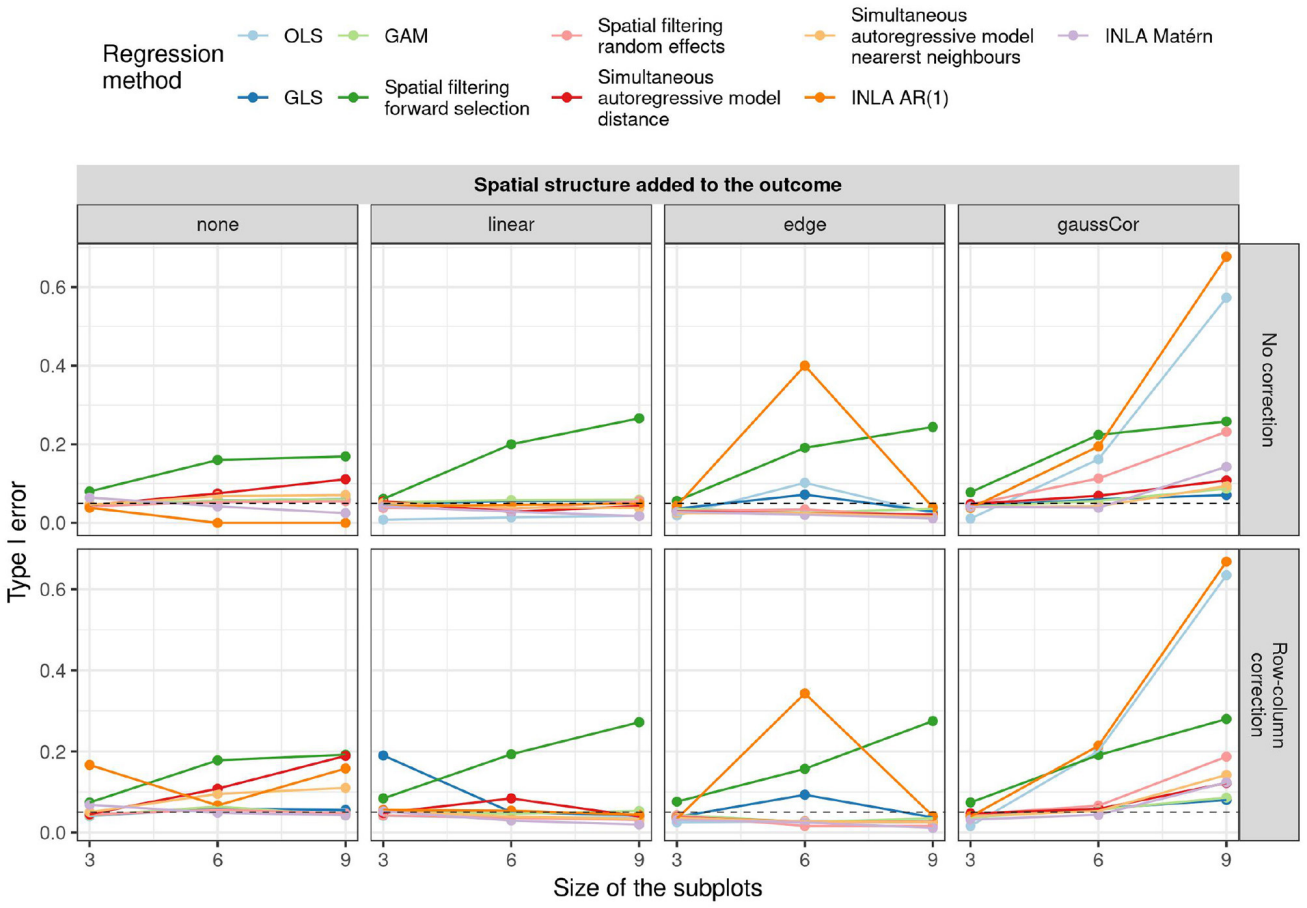
Type I error for the univariate scenario with checkerboard design for different regression methods (colors) for different spatial structures of the outcome (columns) and size of the subplots (x-axis) as a function of whether or not row and column correction was applied (rows). The dashed horizontal line indicates the 5% significance level.

For the checkerboard design, the inflation of the type I error is worst for the case of Gaussian correlation in the outcome, but this inflation is in all cases greatly reduced by using small subplots (Figure 3). Spatial filtering with forward selection generally has a large type I error, presumably because it executes multiple statistical tests in the selection of eigenvalues but only reports the final *p*-value, without accounting for the previous tests. Also OLS, GLS, spatial filtering with random effects, and the autoregressive models have inflated type I error in some scenarios. Note that inflation of the type I error due to edge effect is worst for the intermediate subplot size of 6, especially for INLA AR(1), GLS, and OLS without correction for row/column effects. The reason is that for this design (refer to Supplementary Figure S3), the edge is spatially confounded with the treatment effect because the number of subplots is uneven in this case and either the treatment or the control plots tend to be located toward the edge (refer to Supplementary Figure S7). This result suggests that checkerboard designs are best made with even numbers of subplots to avoid spatial confounding with edge effects. It also explains why there is no inflation of the type I error for the edge effects with other subplot sizes and all linear effects: they are orthogonal to the checkerboard design pattern and hence do not cause spatial confounding. Results on the power and MSE of all methods can be found in Supplementary Figures S18, S19.

### 3.2. Low-Dimensional Scenario

**Figure 4:**
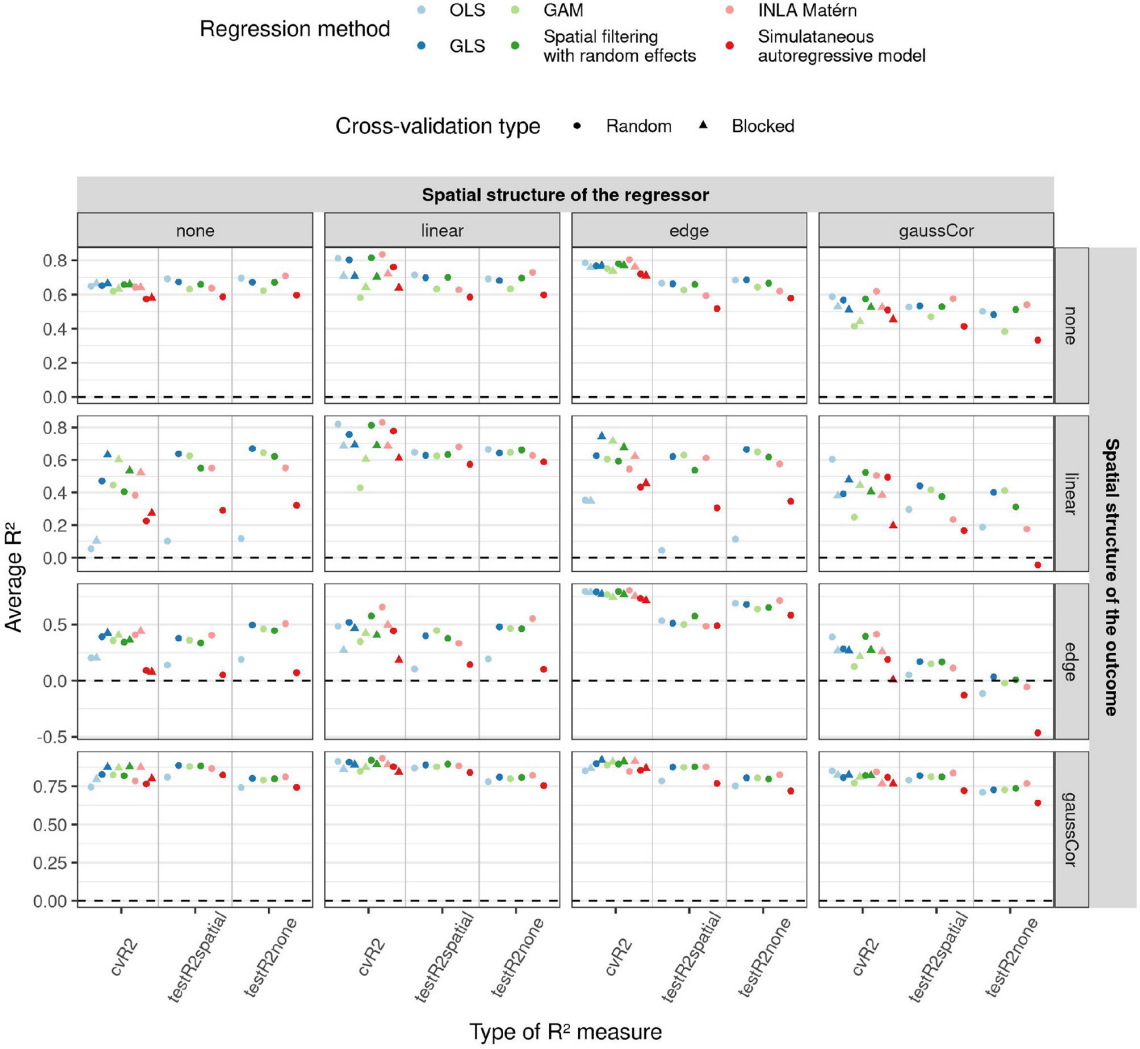
Average *R*^2^ (y-axis) for the low-dimensional scenario estimated by different cross-validation (CV) paradigms (shapes) and test datasets (x-axis) as a function of regression method (color) and of different correlation structures of the regressors (columns) and outcome (rows). In this low-dimensional scenario, the model fit and hence also test *R*^2^ values do not depend on the CV paradigm, so results are only shown for random CV for the test *R*^2^. 1,000 Monte Carlo runs were executed, the standard deviation of the non-zero components of ***β*** were 1. Supplementary Figures S20, S21 show similar figures for standard deviations 0.5 and 2.

An overview of the results for the low-dimensional scenario is shown in Figure 4, more results can be found in Supplementary Figures S20–S23. When the outcome exhibits an edge effect, the testR^2^spatial is generally lower than the testR^2^none, probably because the extra variability of the edge effect cannot be explained by the regressors. When the regressors have a linear or Gaussian spatial structure, random CV overestimates *R*^2^ on unseen test datasets, whereas blocked CV is more accurate. OLS and simultaneous autoregressive models have poor performance on test datasets when there is a linear or edge structure in the outcome, but a different spatial structure in the regressors. For linear structure in the outcome, spatial filtering and INLA Matérn exhibit intermediate performance and GAM and GLS perform best. Spatial filtering and INLA Matérn as well as GAM and GLS perform when there is a Gaussian autocorrelation or edge structure in the outcome; GAM performs poorly in absence of spatial structure in the outcome.

We also ran simulations investigating the role of the number of features. Supplementary Figures S24–S26 show that the difference between the regression methods is generally smaller when built on fewer features. The performance of the simultaneous autoregressive models deteriorates as the number of features increases, even in absence of any spatial pattern. These graphs also reveal that a higher number of features does not always lead to better predictions, as the estimation of the corresponding parameters becomes harder.

**Figure 5:**
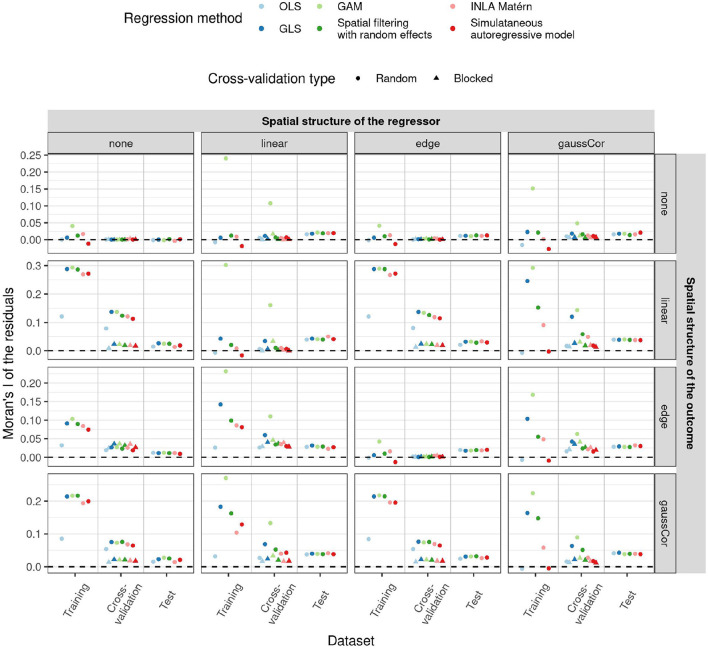
Average Moran's I statistic for spatial autocorrelation (SAC) of the residuals (y-axis) for the low-dimensional scenario estimated in different ways (x-axis) as a function of regression method (color) and of different correlation structures of the regressors (columns) and outcome (rows) in both test and training data. All statistics had their expected value subtracted from them, such that they all have mean 0 under the null hypothesis of no SAC. Shapes reflect the two different CV paradigms. Since in the low-dimensional scenario, the model fit and hence also the training and test Moran's I do not depend on the CV paradigm, results for these quantities are only shown for random CV. 1,000 Monte Carlo runs were executed, the standard deviation of the non-zero components of ***β*** were 1.

Interestingly, despite their poor performance, OLS and simultaneous autoregressive models have the lowest SAC in the residuals of the training data, as measured by the Moran's I statistic (Figure 5). Although the spatial structure cannot be entirely explained by the regressors, i.e., when there is an independent spatial pattern in the outcome, the values of the Moran's I statistic are often close to their expected value under the null hypothesis of no SAC (and hence on the plot close to zero after subtracting the expected value). Yet, when the corresponding models are applied to a new test dataset with spatial structure, there is little difference between the methods in terms of residual spatial autocorrelation. These results suggest that methods ignoring spatial structure, like OLS, try to capture realizations of the spatial effects using the available covariates, yielding low residual SAC in the training data. This gives the false impression of explaining all spatial structures in the outcome through the spatial patterns in the regressors, but this is a case of overfitting the spatial pattern. Just like Moran's I on the test datasets, blocked CV Moran's I was in many cases similar among the methods, indicating that blocked Moran's I CV is a better predictor for differences between the methods in residual spatial autocorrelation than training or random CV Moran's I. For instance, in presence of linear effects in the regressor, GAMs often have large residual SAC in the training dataset and in random CV, but not in the blocked CV or test data with spatial structure.

In summary, GLS generally works best across different SAC scenarios, even when its assumptions on spatial error structure are not entirely fulfilled. An exploration of how GLS accommodates linear and edge effects is given in Supplementary Section 3.

### 3.3. High-Dimensional Scenario

**Figure 6:**
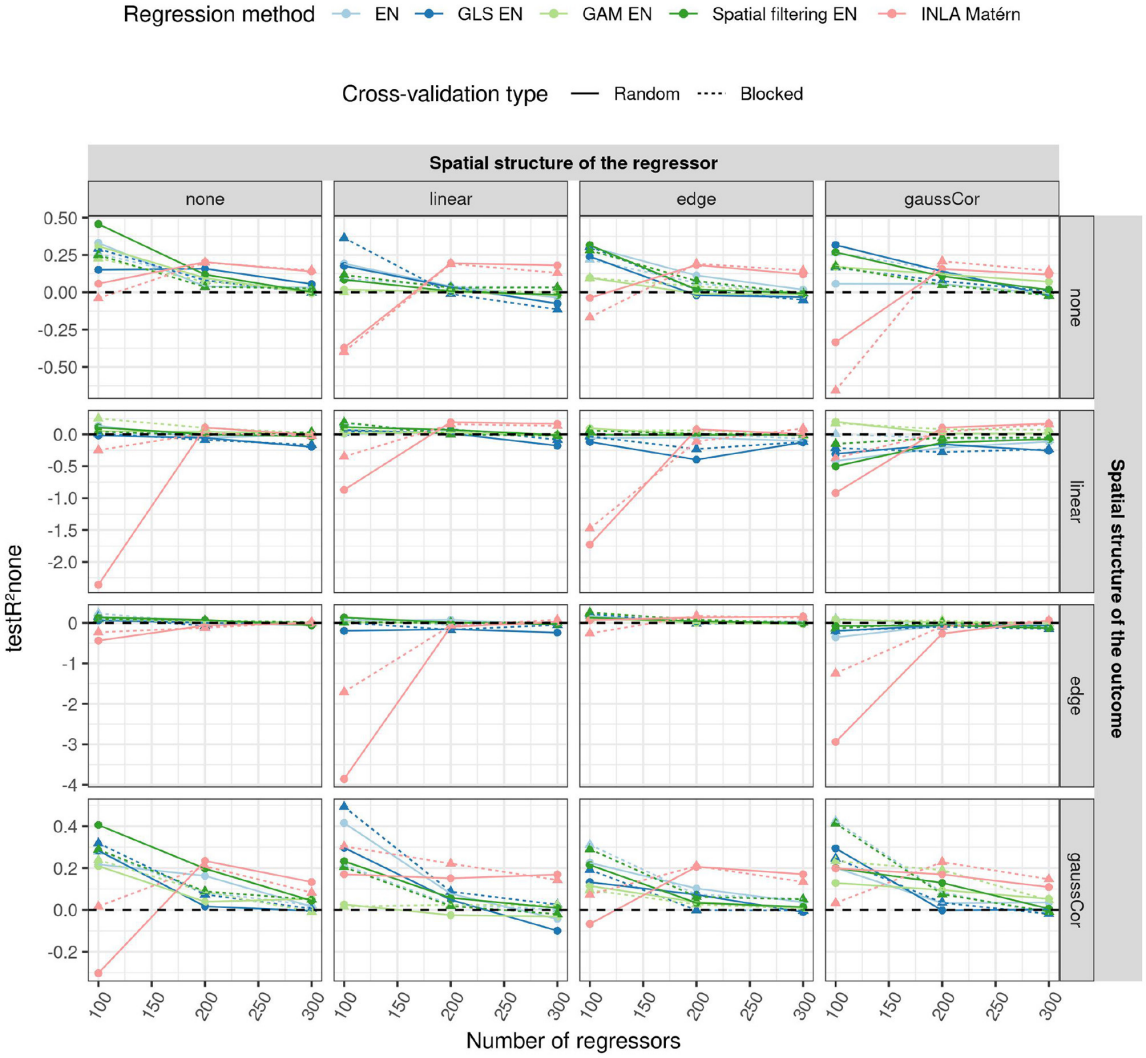
Average *R*^2^ on an independent test dataset without spatial structure (testR^2^none, y-axis) for the high-dimensional scenario when using different CV paradigms (shapes and linetypes) as a function of the number of regressors (x-axis), EN version (colors), the correlation structure of the regressors (columns) and of the outcome (rows). In the high-dimensional case, CV is used to tune the penalty parameter, so the model fit may depend on the CV paradigm. For this reason, test *R*^2^ is shown for both CV paradigms in this case. The standard deviation of the non-zero components of the ***β*** parameter was 0.5. The horizontal dashed line indicates an *R*^2^ of 0.

An overview of the results for the high-dimensional scenario is shown in Figure 6, more results can be found in Supplementary Figures S27–S38. The performance of the prediction models for high-dimensional data is seen to decrease with the number of features (as shown in Figure 6), just like in the low-dimensional scenario (Supplementary Figure S25). An exception is INLA Matérn, which performs worse than the other methods at low numbers of regressors (*p* = 100), but better when the number of regressors is high (*p* = 300). However, its model fitting procedure failed for around 20% of the Monte Carlo instances in all settings.

The feature selection differs much more between the different methods than predictive performance: GLS EN has higher sensitivity in selecting correct predictors in all scenarios, whereas GAM EN has lower sensitivity than the other methods (Supplementary Figure S31). On the other hand, GLS EN has a slightly lower TDP in some scenarios, whereas GAM EN generally has a higher one (Supplementary Figure S32). Overall, the TDP and especially the sensitivity decrease with the number of features, indicating that the task of feature selection becomes more difficult as the dimensionality of the dataset grows. This also explains the decrease in model performance with the number of features, despite the fact that there are more truly predictive features and thus theoretically a higher *R*^2^ (refer to Supplementary Figure S5).

Interestingly, in the presence of spatial patterns in the regressors, regular EN selects more than the expected 50% of features with a spatial structure (refer to Supplementary Figure S33), the so-called “red-shift” (Lennon, 2000). EN with spatial filtering only suffers from this red-shift when there are linear gradients or edge effects in the data. GLS EN appears less sensitive to red-shift, selecting around 50% of spatial features in case of linear patterns or Gaussian correlation in the regressors. Yet, in the case of edge effects in the regressors, GLS EN also selects excess spatial features. GAM EN on the other hand, often selects less than 50% spatial features.

Generalized additive model EN generally has a higher SAC in the residuals than competing methods when modeling training data or left-out CV folds, in particular when there are linear trends in the regressors. INLA Matérn on the other hand has a lower residual SAC when modeling training data or test data with linear trends in the outcome or regressors (refer to Supplementary Figures S34–S37).

### 3.4. Case Study: Predicting Winter Wheat Yield Based on Multispectral Measurements

#### 3.4.1. Data Description

The scatterplots and Pearson correlations in Supplementary Figures S12–S15 indicate strong multicollinearity between the multispectral variables, but less correlation between yield and especially protein content on the one hand and the multispectral variables on the other. PCA biplots in Supplementary Figure S16 suggest a similar covariance pattern between all variables over the 4 different fields, although *nir* and *evi*2 are less correlated with the other variables in fields 3 and 4 than in fields 1 and 2. Average yields differ between the fields, as shown in Supplementary Figure S11, hence a correction for a different baseline is needed. Field 2 has the strongest autocorrelation in almost all variables, followed by field 1 (refer to Supplementary Figure S39).

#### 3.4.2. Prediction Model Performance

**Figure 7:**
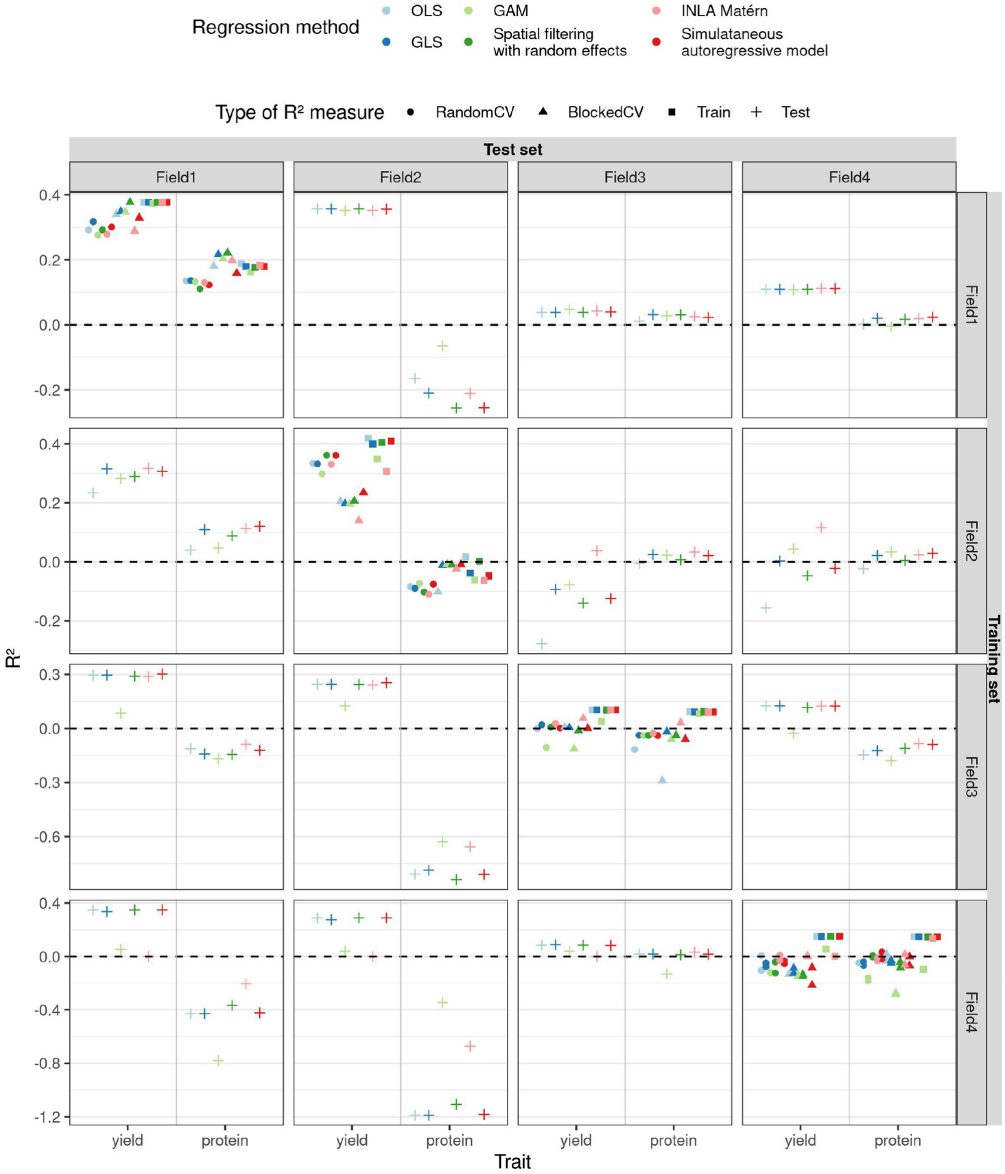
*R*^2^ (y-axis) estimated in different ways (shapes) for the two traits (x-axis) as a function of different training and test fields (rows and columns, respectively) and regression methods (color) for the case study on winter wheat.

Predicted vs. observed yield and protein content are shown in Supplementary Figures S40, S41. The CV, training, and test *R*^2^ are shown in Figure 7, estimated ***β*** parameters of the principal components with standard errors can be found in Supplementary Figure S42. We see that the standard errors of the parameter estimates increase with the order of the principal component, but do not really depend on the method. An exception is the INLA Matérn model trained on field 4, which has small estimates and large standard errors. The sums of the products of the parameters with principal component loadings ***β*****u1**, with **u** the matrix of principal components included in the model and **1** a column vector of ones, are plotted in Supplementary Figure S43. Overall, we find lower values of *R*^2^ than reported by the authors (Zhou et al., 2021). This is likely because we 1) split the data into separate fields, rather than scrambling data from all fields together, 2) use smaller training datasets and 3) do not ensure equal variances in training and test set. Yet, our objective was to estimate generalisability of the models between fields, which is different from the objective of the original paper.

For yield, we observe that when training on field 2, the field with the strongest SAC, the blocked CV *R*^2^ estimate is lower than the random one (as shown in Figure 7). When trained on this field, OLS performs poorly on test sets, as its predictions are often too extreme (refer to Supplementary Figure S40), and also its parameter estimates are more extreme than for the other methods (refer to Supplementary Figure S42). Also for field 2, OLS has a smaller SAC of the residuals of the training data than the other methods (refer to Supplementary Figure S44). These results are in accordance with the results of our simulation study: SAC deteriorates the performance of OLS and causes blocked and random CV *R*^2^ estimates to diverge, but residuals of the training data of OLS are not spatially autocorrelated. The GAM models for yield trained on fields 3 and 4 perform worse on other fields than the other methods, their predictions being too extreme for the model trained on field 3 and too average for the model trained on field 4 (refer to Supplementary Figure S40). The corresponding parameter estimates of GAM follow the same pattern (refer to Supplementary Figure S42). This had been predicted by CV (for field 4 only by random CV) and is reminiscent of the poor performance of GAM on data with Gaussian correlation found in the simulation study. The INLA Matérn model trained on field 2 generalizes best to fields 3 and 4, but on the other hand, the INLA Matérn model trained on field 4 has low training and test *R*^2^.

For models predicting protein content, the model performances are often worse, in concordance with the authors who noticed this phenotype was harder to model using linear models (Zhou et al., 2021). Prediction is also complicated by large outlying values in fields 3 and 4 (refer to Supplementary Figure S41). Yet, also here, OLS performs slightly worse than other models on test fields when trained on field 2, as predicted by blocked CV *R*^2^ only. Blocked CV on field 3 also predicted a lower *R*^2^ for OLS, but this is not seen in the test *R*^2^. For fields 1 and 2, the blocked CV *R*^2^ is higher than random CV *R*^2^ for all model types, but this is only accurate according to the test *R*^2^ values for the protein content models trained on field 2. The simultaneous autoregressive model, INLA Matérn, and spatial filtering methods perform overall similarly to GLS. Yet our simulation results suggest that the simultaneous autoregressive model performs well in this case thanks to the low number of features, but that its performance would deteriorate as the number of features increases (refer to Supplementary Figure S24). The good performance of the spatial filtering method suggests that the setting of this case study is more akin to the Gaussian correlation or edge effect scenario than to linear effects, as could be expected from Supplementary Figure S10.

Looking at generalisability of the yield models, the yield is best predicted for fields 1 and 2, regardless of which field the models were trained on. On the other hand, the yield on fields 3 and 4 is harder to predict. The training *R*^2^ on fields 3 and 4 is even lower than the test *R*^2^ when the corresponding models are applied to fields 1 and 2, which indicates that simply more variability in yield in fields 1 and 2 can be explained by the multispectral variables measured. This is a different situation from the simulation study, where the residual variance σ^2^ was kept constant, allowing us to use CV *R*^2^ as an estimator for test *R*^2^. Nevertheless, CV was able to predict relative differences in performances in the case study and can be a great help in this respect. Despite higher noise levels in fields 3 and 4, the models trained on them are not less accurate than the models trained on field 1. The least accurate models are the ones trained on field 2, illustrating how SAC deteriorates model estimation, even when suitable spatial regression models are used.

## 4. Discussion

In this study, we compare a number of regression methods for data with spatially autocorrelated variables. Simultaneous autoregressive models were only found to work well when the number of regressors is low. Spatial filtering methods performed well in the case of unstructured SAC, but struggled to deal with structured forms of SAC such as linear gradients and edge effects. Generalized additive models (GAMs) account for SAC by explicitly modeling the dependence of the outcome on the location, which yields good insights into the spatial structure of the outcome variable. On the downside, GAMs have a high efficiency cost when there is no long-range spatial dependence in the data. Moreover, GAMs tend to absorb too much of the spatial structure of the outcome in their smooth spatial term, thereby underrating the ability of the available regressors to explain this spatial structure. This overcorrection can lead to a veritable “blue-shift,” whereby regressors with spatial signals are less likely to be recognized as associated with the outcome. The INLA model based on a Gaussian random field with Matérn covariance structure performed reasonably well in both low- and high-dimensional scenarios. When the dimensionality of the data is much higher than the sample size, the INLA Matérn model even outperforms the elastic net models in terms of prediction accuracy. On the downside, its Bayesian model-fitting algorithm may fail to converge, and no feature selection in the high-dimensional scenario is provided. The GLS method was found most robust in accounting for different forms of SAC, in both low and high-dimensional settings. In the low-dimensional scenario, GLS comes with a slight efficiency loss compared to OLS when there is no spatial structure, but outperforms OLS by a large margin in most settings with spatial correlation. For the high-dimensional setting, we newly combined GLS estimation of the covariance structure of the residuals with a penalized least squares method (elastic net). Although we found no improvement in terms of predictive performance, and in some scenarios even a slight decrease, we have shown how it improves upon regular elastic net with respect to feature selection. In the presence of spatial effects, GLS elastic net has higher sensitivity in detecting good predictors and is less sensitive to red-shift (selecting excess features with spatial patterns) than competing methods. This confirms the trade-off between predictive performance and feature selection as described by Wang and Zhu (2009): there are no single best methods for all scientific aims. As a recommendation to the reader, the methods that performed best in the various simulation scenarios are summarized in Table 1.

**Table 1:** Best methods for different scenarios (first row) and criteria (second and third rows).

**Univariate**	**Low-dimensional**	**High-dimensional**			
Hypothesis testing	Prediction	Prediction	Feature selection		
			Sensitivity	TDP	Robustness to red-shift
GLS, GAM	GLS	EN for moderately high dimensions, INLA for very high dimensions	GLS EN	GAM EN	GLS EN

*TDP, true discovery proportion; GLS, generalized least squares; GAM, generalized additive model; EN, elastic net. See text for details*.

In summary, for most purposes, we recommend the use of GLS to account for SAC effects in regression models. GLS has the flexibility to accommodate many forms of autocorrelation in its error structure (refer to Supplementary Figure S45), e.g., anisotropic effects whereby the SAC does not decay equally fast with distance in all directions, or temporal autocorrelation. GLS can be combined with many statistical and machine learning methods through a simple iterative loop, even when this increases computation time (refer to Supplementary Section 4). Note that the iterative procedure we propose is different from simple preconditioning on the marginal spatial covariance structure of the outcome, which has been condemned before for rendering the design matrix ill-conditioned (Jia and Rohe, 2015). In our proposal, as in most regression models, the distribution of the outcome (including its covariance structure) conditional on the regressors is of interest. GLS could also be extended to generalized linear models (GLMs) through their formulation as iteratively reweighted least squares. Yet despite GLS's good performance, the estimation problem of its spatial covariance matrix is so ill-posed that its components (range, nugget, and residual variance) are strongly correlated and hence very variable (refer to Supplementary Section 3).

Since GLS and some other spatial methods account for spatial structure in the errors, the residuals of these models may remain spatially autocorrelated, which is not problematic. The main purpose of a regression method is to estimate a relationship between regressors and outcome, which helps to explain some of the total variance of the outcome. The degree to which this succeeds can be measured by *R*^2^. Yet, a low *R*^2^ does not necessarily imply that the model is wrong, or that its parameter estimates are inaccurate, as a low *R*^2^ may also result from missing important covariates or strong background noise in the training data. A predictive model trained on a noisy dataset with low training *R*^2^ may perform much better on a test dataset with low noise levels, as was demonstrated in the case study on winter wheat. Analogously, as a side effect of model fitting, regression methods may explain part of the marginal spatial autocovariance (as for instance measured by the Moran's I statistic Moran, 1950) in the outcome variable through the spatial patterns found in the regressors, leading to a reduced spatial autocovariance in the residuals. Yet, this is not the primary purpose of the regression method, and a large residual spatial autocovariance does not imply inaccurate estimation of the relationship between outcome and regressors. The residual spatial autocovariance may, just like residual variance, result from missing covariates or spatially autocorrelated noise. If almost all spatial autocovariance can be explained by the regressors (corresponding to the simulation scenarios without additional spatial patterns applied to the outcome), methods ignoring spatial structure work fine and are slightly more efficient than spatially aware methods, at least in the low-dimensional scenario. However, when not all spatial autocovariance can be explained by the regressors (the simulation scenarios with additional spatial patterns applied to the outcome), a method like OLS that ignores spatial structure will nevertheless attempt to fulfill its assumption of i.i.d. errors. This can lead to small residual spatial autocovariance in the training dataset, but also to variable or biased parameter estimates and preferential selection of regressors with spatial patterns (the “red-shift” Lennon, 2000). Hence, the absence of spatial autocovariance in the training residuals is not a guarantee that the spatial structure of the data has been well-modeled (Beale et al., 2010; Roberts et al., 2017), and the model may still fail to explain spatial autocovariance in a new dataset (refer to Figure 5). Since it is hard to predict whether all spatial autocovariance is explicable by the regressors, and the efficiency cost of accounting for spatial structure is low, we recommend always using spatially aware methods when analyzing datasets with potential for spatial structure, such as field trials. Finally, note that the opposite occurrence is indeed problematic: when spatially naive methods like OLS nevertheless have residual SAC, this points at problems with the model fit.

Past comparative simulation studies generated data in an unstructured way (Wang and Zhu, 2009; Beale et al., 2010; Alesso et al., 2019; Rocha et al., 2019, 2021; Mao et al., 2020; Harisena et al., 2021), ignoring the fact that spatial patterns in real fields often take on discernible shapes such as edge effects or linear gradients (Austin and Blackwell, 1980; Langton, 1990; Romani et al., 1993; Haase, 1995; Sarker and Singh, 2015; Barmeier and Schmidhalter, 2016; Cruz et al., 2020; Zhou et al., 2021), and despite linear trends and row- and column effects often being included in the analysis model of real data as well (Singh et al., 2003; Sarker and Singh, 2015). In this study, we have included the linear trend and edge effects scenarios as well and found that they can have a profound effect on model performance. Edge effects are difficult to account for, inflating type I error in hypothesis tests and deteriorating model performance on test fields. Hence, they should preferably be avoided by adequate field trial design. Linear gradients in outcome and regressors are also challenging, as they can easily become spatially confounded in the two-dimensional space of a field. Yet, these linear effects can be captured rather well by spatial regression methods such as GAM and GLS. In theory, one could also account for linear or edge effects in the mean model of an OLS regression when their presence is known beforehand, or obvious from the data. Yet in practice, these effects may not be easily detectable, and combinations of different kinds of spatial patterns may be present in the data, such that robust spatial regression methods seem to be a safer choice.

In the setup of our simulation study, we let linear and edge effects increase the variance of a variable. Whether this assumption is correct is difficult to verify. A higher variance in the outcome variable complicates its prediction, whereas a higher variance in a regressor variable renders the model estimation more precise. More commonly used autocovariance simulation schemes make nearby observations more similar, which reduces the information content of the dataset and increases the variance of parameter estimators. The effect on feature selection is similar though: both short range positive SAC and long range linear gradients and edge effects cause excess selection of spatial features in hypothesis testing and penalized regression.

Correction for spatial effects in field trials is common, but often only occurs between discrete plots of checkerboard designs (Lado et al., 2013; Elias et al., 2018; Alesso et al., 2019; Rocha et al., 2019; Mao et al., 2020; Harisena et al., 2021). Yet in our own simulations, we see that such corrections have only limited effect, and SAC within and across discrete plots can still inflate type I errors and estimator variability. It has been demonstrated that the use of more, smaller subplots can combat this (Alesso et al., 2019), but may involve more work and is only possible at the design stage. On top of using small subplots, we, therefore, recommend spatially aware regression methods such as GLS and GAM for the analysis of checkerboard designs, as they yield good power while controlling type I error rate.

It is known that CV can misestimate model performance on an independent test dataset (Rocha et al., 2018). On the one hand, the model may overfit the training set, and on the other hand, noise levels may differ between training and test set, rendering measures such as mean squared error (MSE) of the predictions and *R*^2^ hard to compare. Yet, this problem may be exacerbated by spatial patterns in the training data. SAC causes information leaks between the training set and left-out fold, violating the independence assumption and causing estimates of model performance to be overoptimistic. It has been shown that this problem can be mitigated, but often not eliminated, by blocked CV, i.e., choosing the folds as blocks of neighboring observations (Brenning, 2005; Pohjankukka et al., 2017; Roberts et al., 2017; Meyer et al., 2019; Schratz et al., 2019; Ploton et al., 2020). We confirm that blocked CV is better able to discriminate between competing methods in the presence of SAC, and is similar to random CV in absence of such autocorrelation. Hence, we recommend blocked CV as a simple and widely applicable solution in all scenarios where SAC may be present. Other corrections for CV on dependent data have been proposed (Rabinowicz and Rosset, 2020), but these are restricted to a smaller class of methods and were not tested here. Furthermore, it has been hypothesized before that a change in CV paradigm could affect hyperparameter tuning (Schratz et al., 2019), but this was not found in our study. Another problem with SAC arises when the test dataset also exhibits spatial patterns that are not completely explicable by the regressors. This causes additional deviations, with a spatial pattern, of the observed values from the expected ones (Rocha et al., 2018).

In summary, we believe that field trials, either observational or experimental, are a great way to learn relationships between variables if the following points are taken into consideration. In simulation studies for spatial data, we recommend the inclusion of more realistic spatial structures such as edge effects and linear gradients. In checkerboard designs, the use of many, small subplots mitigates spatial confounding. For the analysis of data with spatial signatures, we support the use of blocked CV. Moreover, we suggest the inclusion of spatial covariance matrices in regression models, including high-dimensional ones, through GLS, and warn against model evaluation through residual SAC of the training dataset only.

## Data Availability Statement

The data analyzed in this study is subject to the following licenses/restrictions: The dataset is available from Zhou et al. upon request. Requests to access these datasets should be directed to takashit@gifu-u.ac.jp.

## Author Contributions

SH and SD conceived the study. SH performed the analyses. SH wrote the manuscript with input from SD and SM. SM supervised the work. All authors interpreted the results.

## Funding

SH was supported through a research collaboration with Inari Agriculture NV. SD is a fellow of the Research Foundation-Flanders (FWO, grant 1146319N).

## Conflict of Interest

The authors declare that the research was conducted in the absence of any commercial or financial relationships that could be construed as a potential conflict of interest.

## Publisher's Note

All claims expressed in this article are solely those of the authors and do not necessarily represent those of their affiliated organizations, or those of the publisher, the editors and the reviewers. Any product that may be evaluated in this article, or claim that may be made by its manufacturer, is not guaranteed or endorsed by the publisher.
