# Supplementary material for: Spatial Regression Models for Field Trials: A Comparative Study and New Ideas
Source: Front Plant Sci. 2022 Mar 30;13:858711. doi: 10.3389/fpls.2022.858711 (PMC9006620; doi:10.3389/fpls.2022.858711)
Supplement: Supplementary file 1 [file Data_Sheet_1.pdf]

Supplementary material for ‘Regression models for field trials: a comparative study and new ideas’

Stijn Hawinkel, Sam De Meyer and Steven Maere

Contents

1 Materials and methods **1**
1.1 Simulation study . . . 1
1.2 Case study . . . 8

2 Results **14**
2.1 Univariate scenario . . . 14
2.1.1 Experimental studies: checkerboard design . . . 14
2.2 Low-dimensional scenario . . . 16
2.2.1 The role of the number of features . . . 21
2.3 High-dimensional scenario . . . 25
2.4 Case study . . . 38

3 A closer look at parameter estimation in the GLS method **44**
3.1 Point estimates . . . 44
3.2 Correlations of the parameter estimators . . . 44

4 Computation times **46**

5 Software versions **46**

1 Materials and methods

1.1 Simulation study

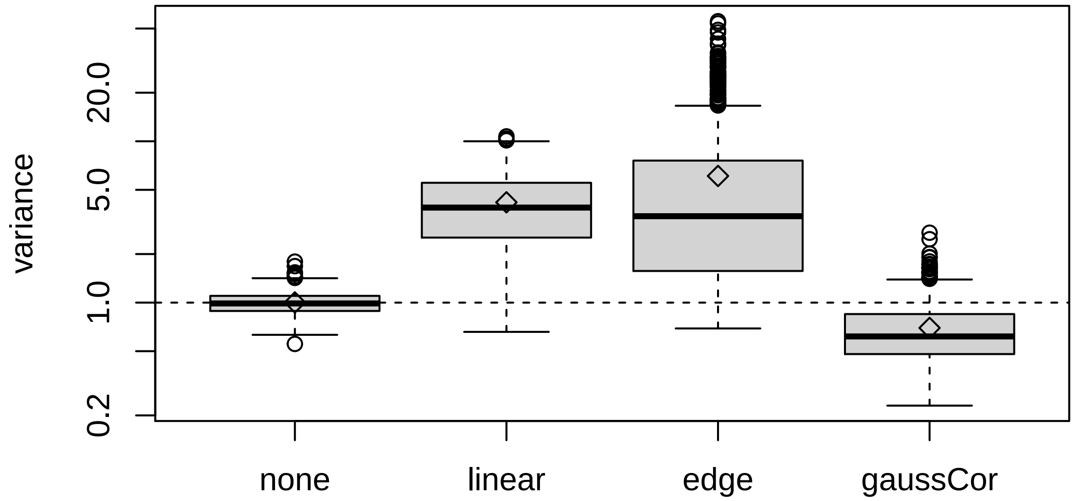

Figure S1: Variance of a random variable (y-axis) generated with 4 spatial structures (x-axis) under parameter values specified in the main text, for 1,000 Monte-Carlo instances. The dashed horizontal line indicates a variance of 1. Diamonds indicate means; the y-axis is on the log-scale. It is clear that Gaussian correlation reduces the variance, whereas linear and edge effects increase the variance of the variable.

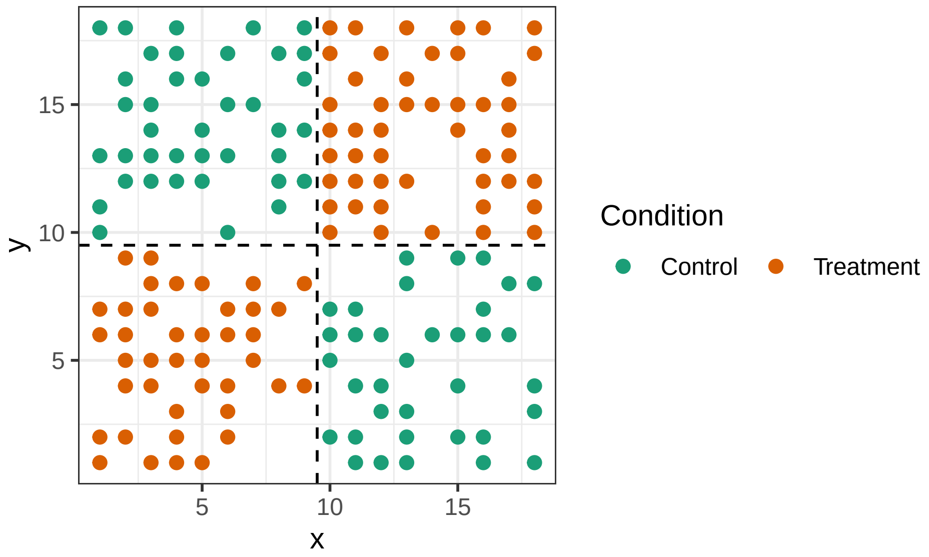

Figure S2: Example of the first checkerboard design for univariate simulation with subplot size of 9. x- and y-axes represent coordinates, colour reflects the grouping factor B. Dashed lines indicate borders of subplots.

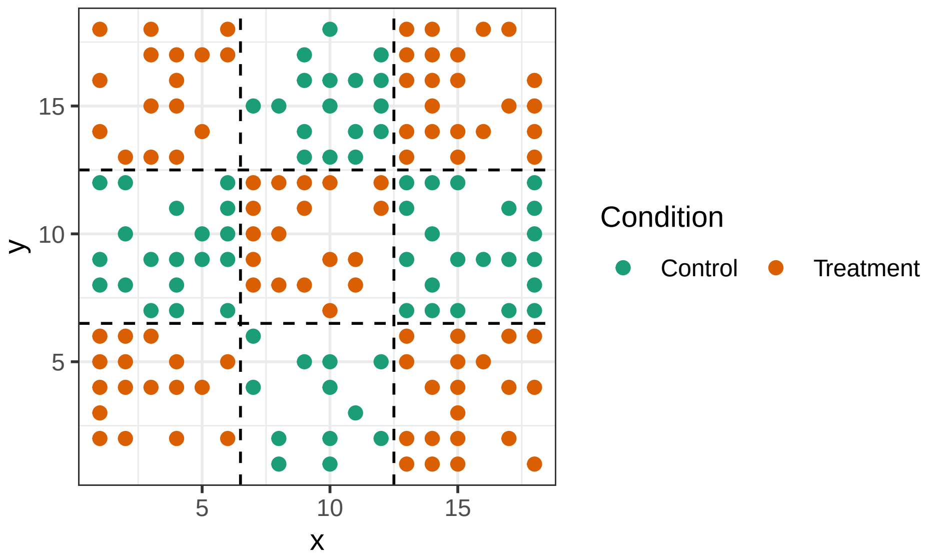

Figure S3: Example of the second checkerboard design for univariate simulation with subplot size of 6. x- and y-axes represent coordinates, colour reflects the grouping factor B. Dashed lines indicate borders of subplots.

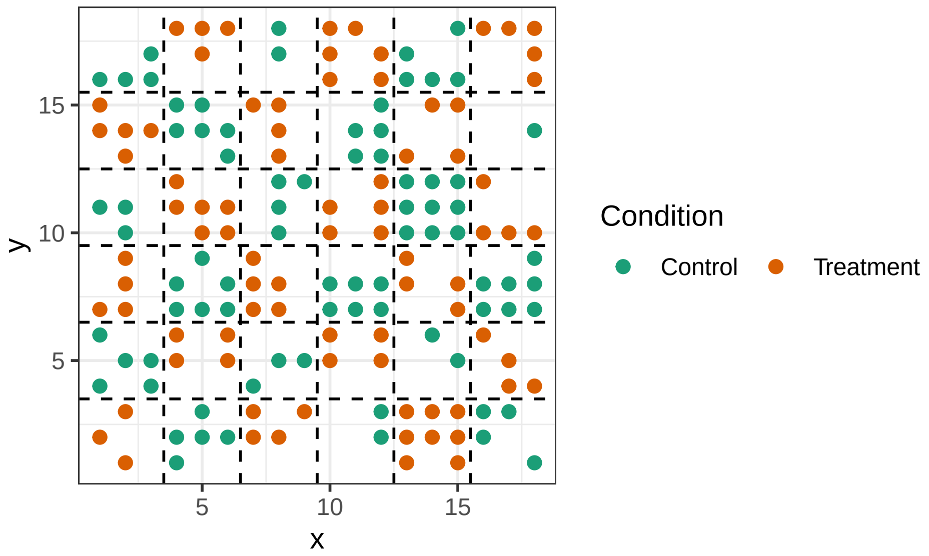

Figure S4: Example of the third checkerboard design for univariate simulation with subplot size of 3. x- and y-axes represent coordinates, colour reflects the grouping factor B. Dashed lines indicate borders of subplots.

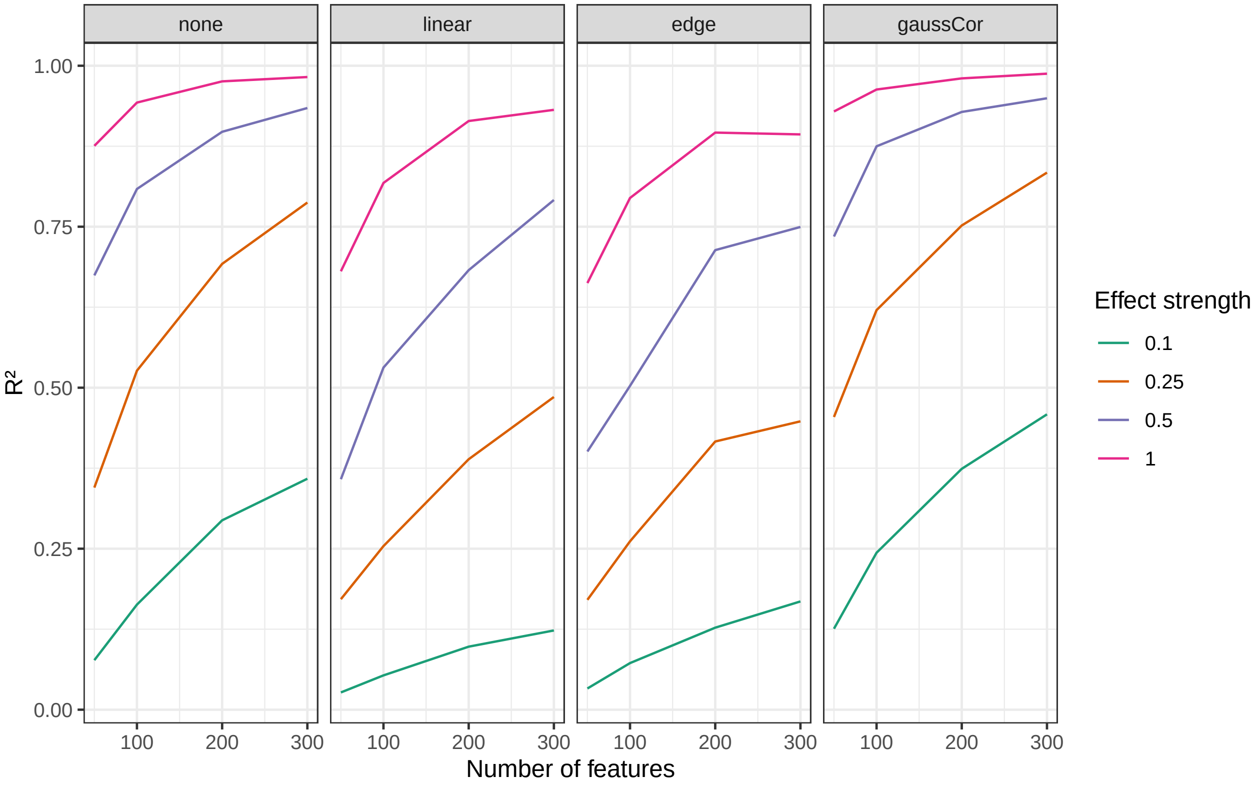

Figure S5: R^2 values (y-axis) when using true parameter values $\boldsymbol{\beta}$ (rather than its estimate based on the data) for different standard deviations of the parameter components (colours), number of features (x-axis) and spatial structure of the outcome (columns) over 100 Monte-Carlo instances. Linear and edge effects lead to a larger residual variance and thus a smaller R^2.

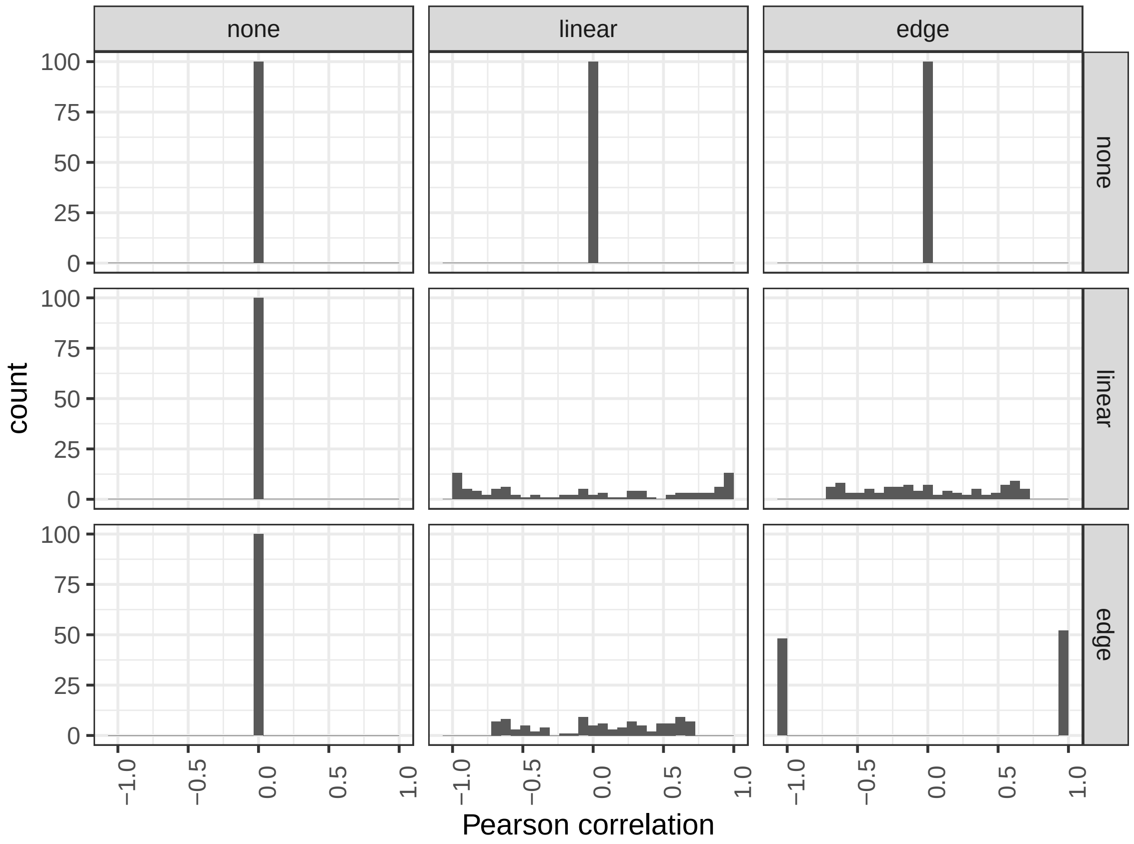

Figure S6: Histograms of correlations between mean outcome and mean regressor (x-axis) of the observational study design for 100 Monte-Carlo instances with different combinations of spatial structures of the outcome (rows) and regressor (columns).

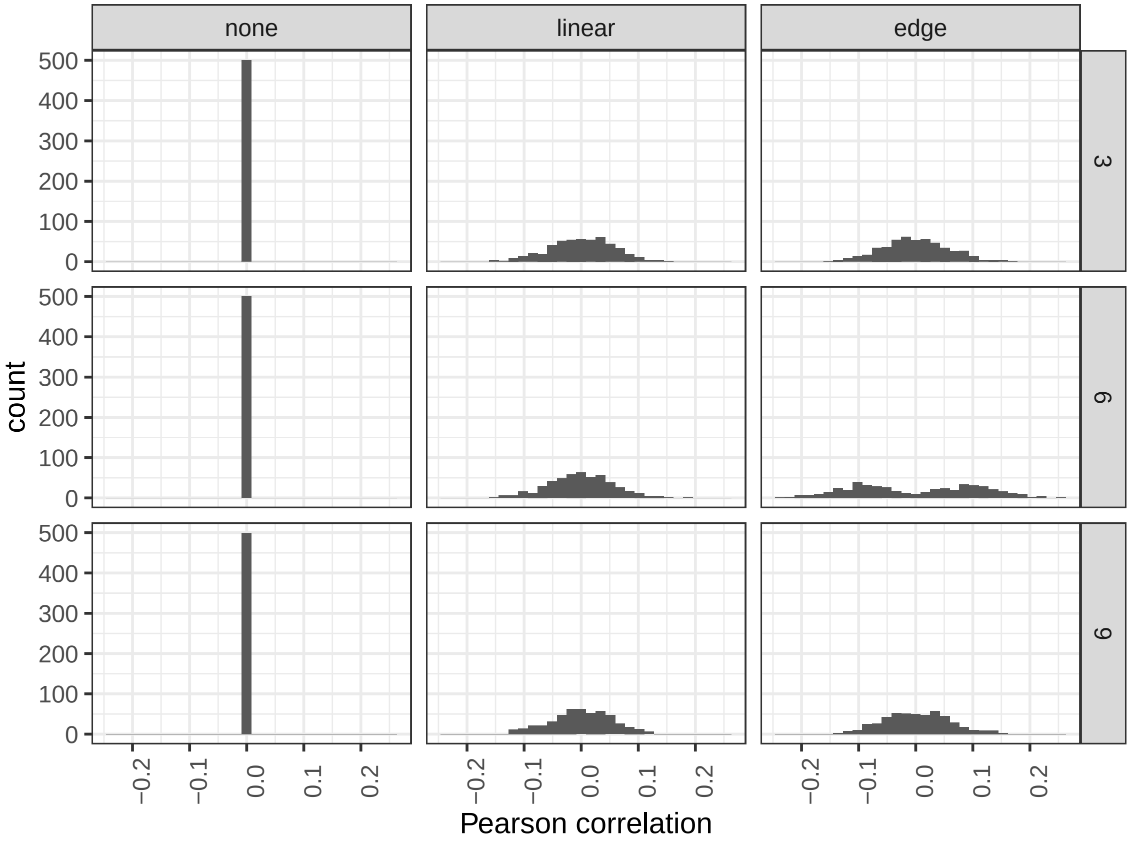

Figure S7: Histograms of correlations between mean outcome and treatment (x-axis) of the checkerboard design for 500 Monte-Carlo instances with different combinations of spatial structures of the outcome (columns) and subplot sizes (rows). The correlation distribution for edge effect with subplot size of 6 is clearly bimodal, indicating true underlying correlation.

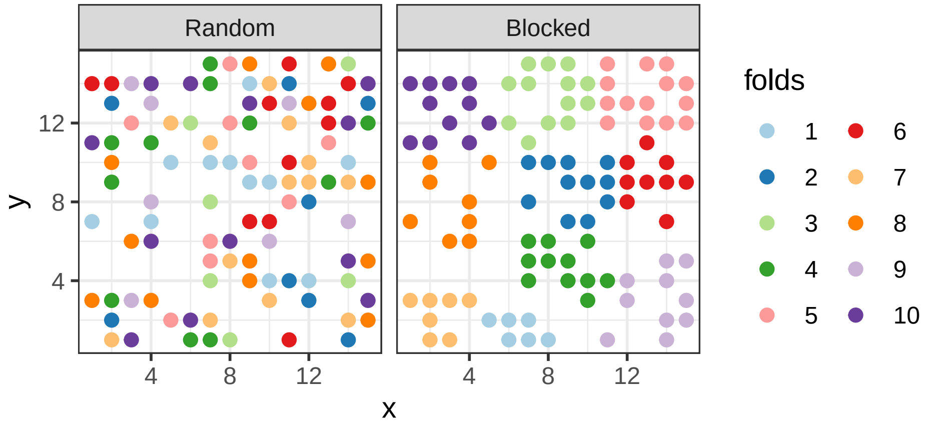

Figure S8: Example setup of random and blocked cross-validation. x- and y-axes respresent spatial coordinates.

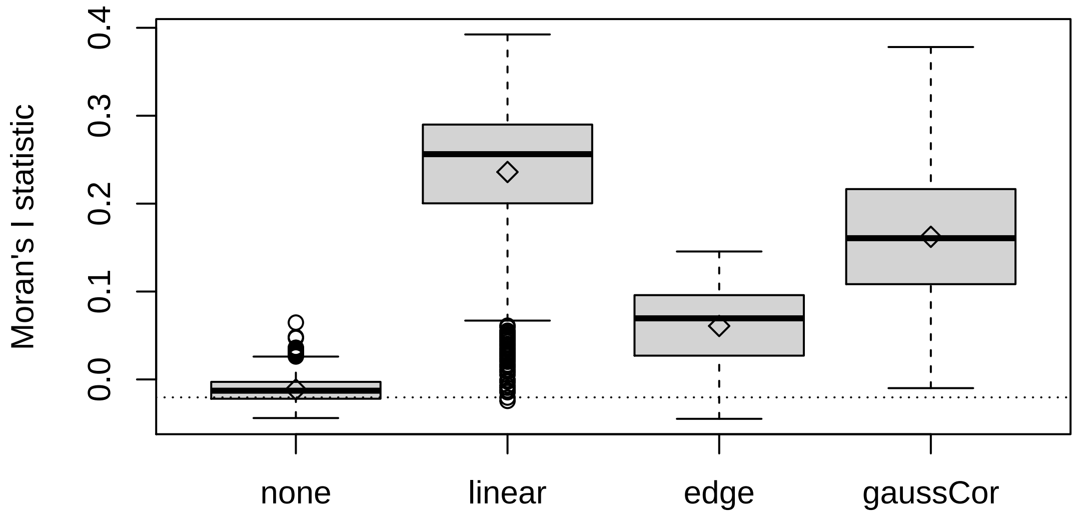

Figure S9: Moran's I statistic of outcome variables (y-axis) generated with 4 spatial structures (x-axis) under parameter values specified in the main text, for 1,000 Monte-Carlo instances. Diamonds indicate means, the dotted horizontal line the expected value of the statistic in absence of spatial structure. Moran's I with weights decreasing with distance is poorly suited for detecting edge effects.

1.2 Case study

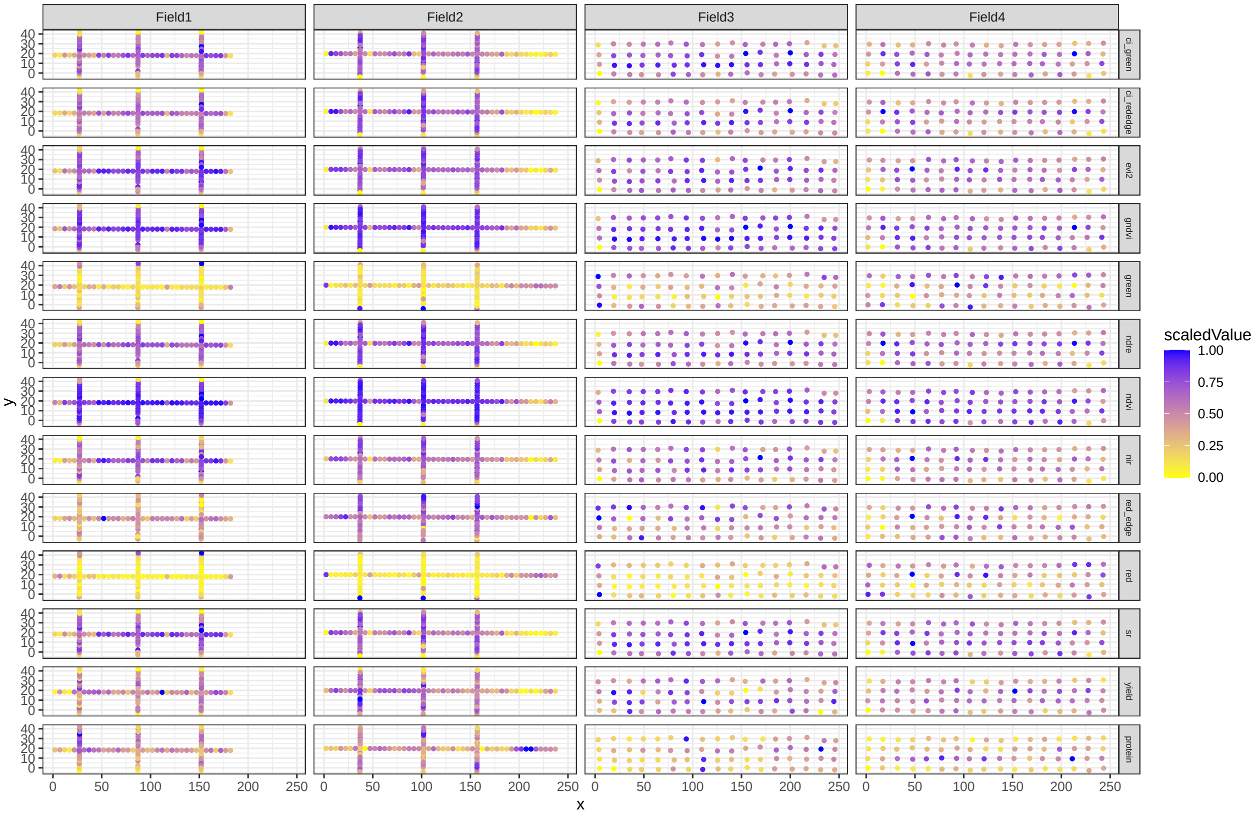

Figure S10: Heatmap of zero-one scaled extracted multispectral features and yield variables (rows) for different fields (columns) for the Zhou2021 dataset (see Zhou *et al.* [2] for an explanation of the variables). In fields 1 and 2, three lateral and one longitudinal transect were measured in a full field, in fields 3 and 4, the sampling was even over the field. x- and y-axes are field coordinates.

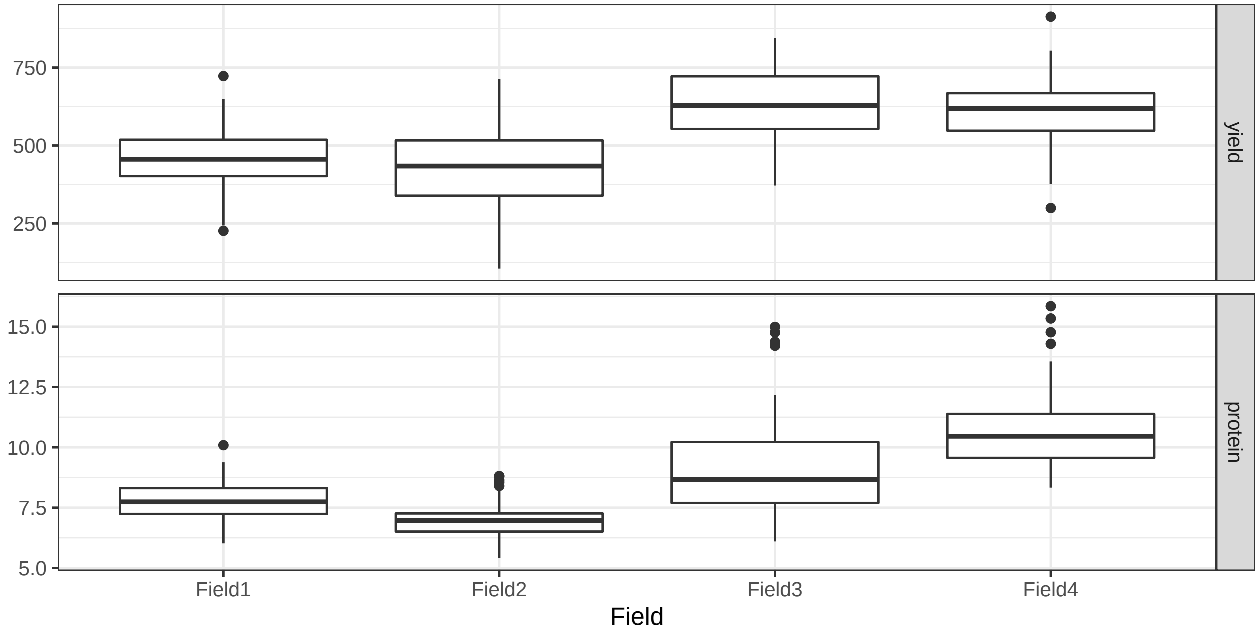

Figure S11: Boxplots of yield variables (rows) of different fields (x-axis).

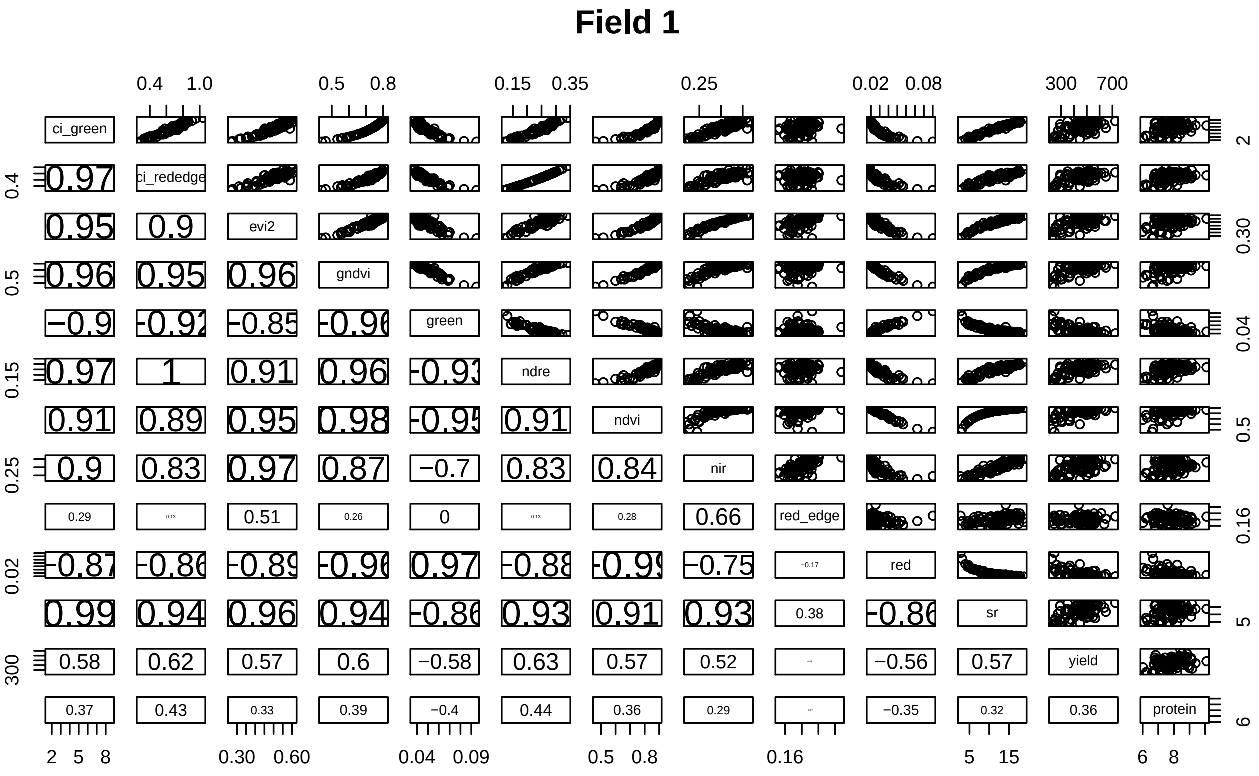

Figure S12: Scatterplots (top right) and Pearson correlations (bottom left) between variables from field 1.

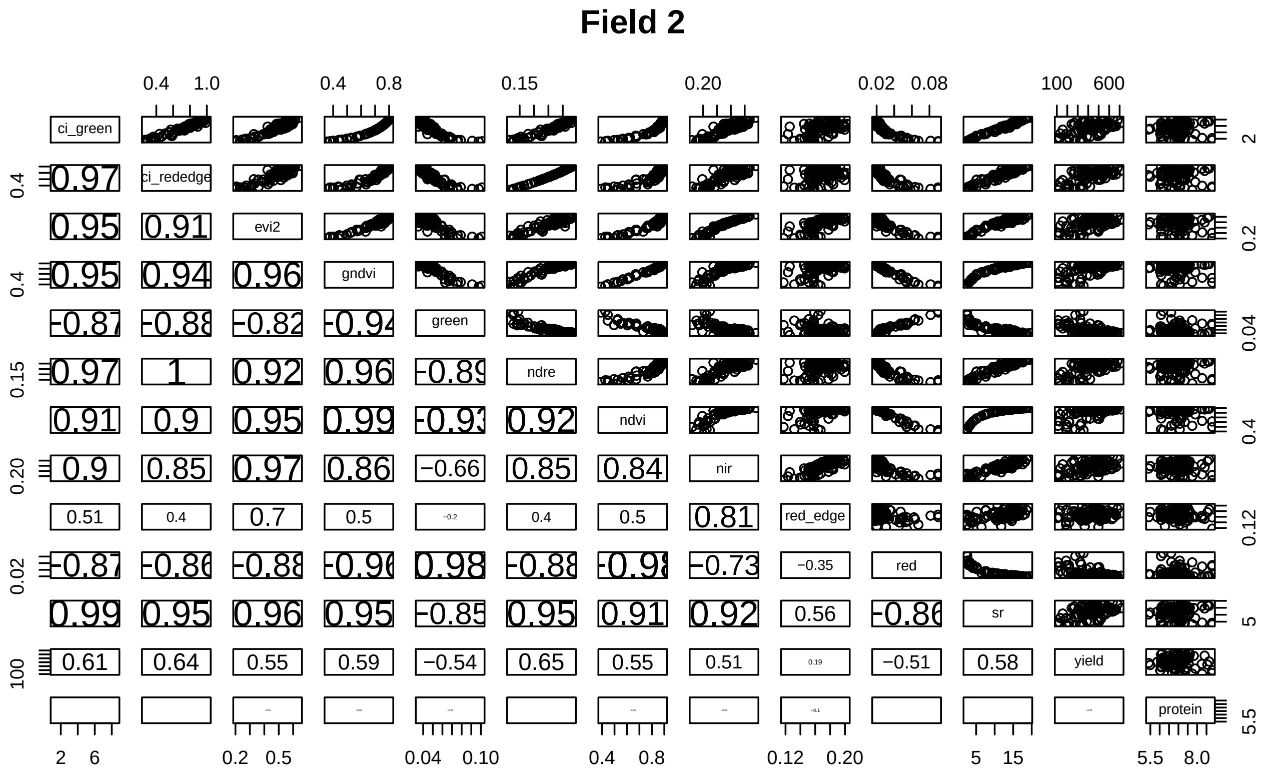

Figure S13: Scatterplots (top right) and Pearson correlations (bottom left) between variables from field 2.

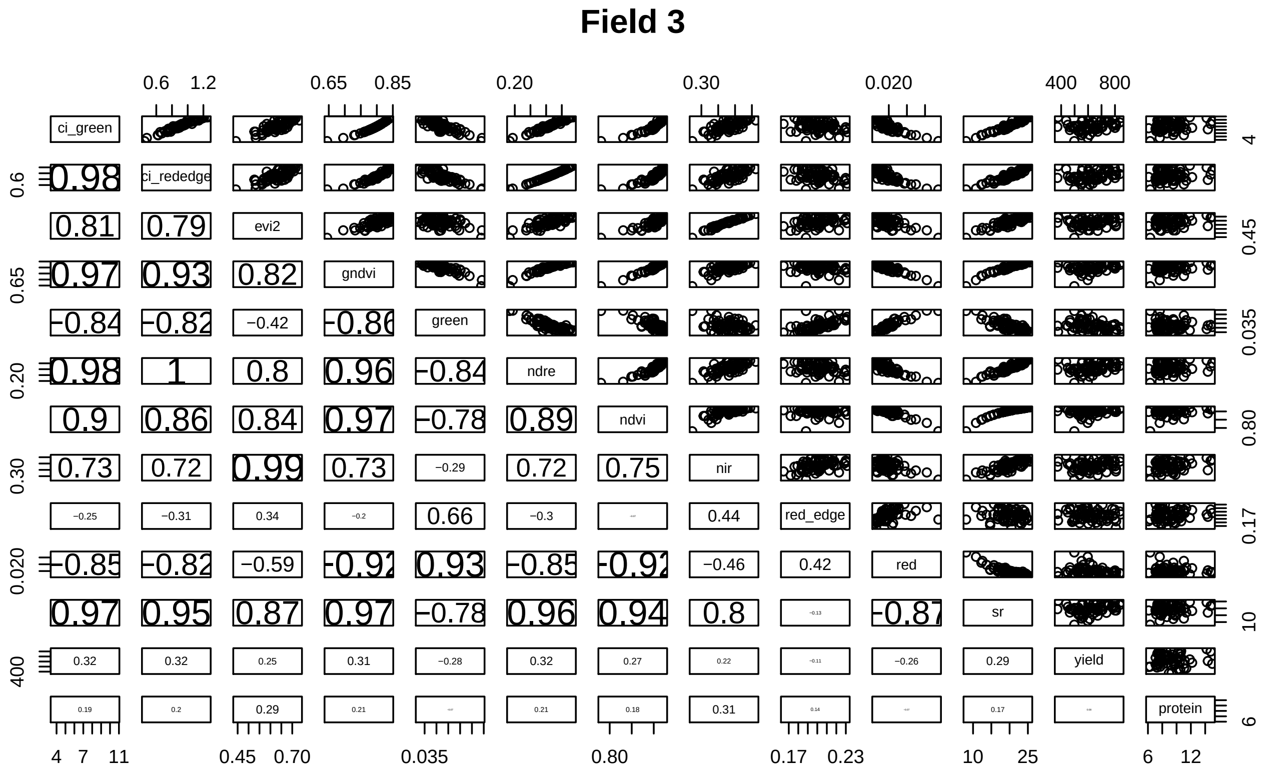

Figure S14: Scatterplots (top right) and Pearson correlations (bottom left) between variables from field 3.

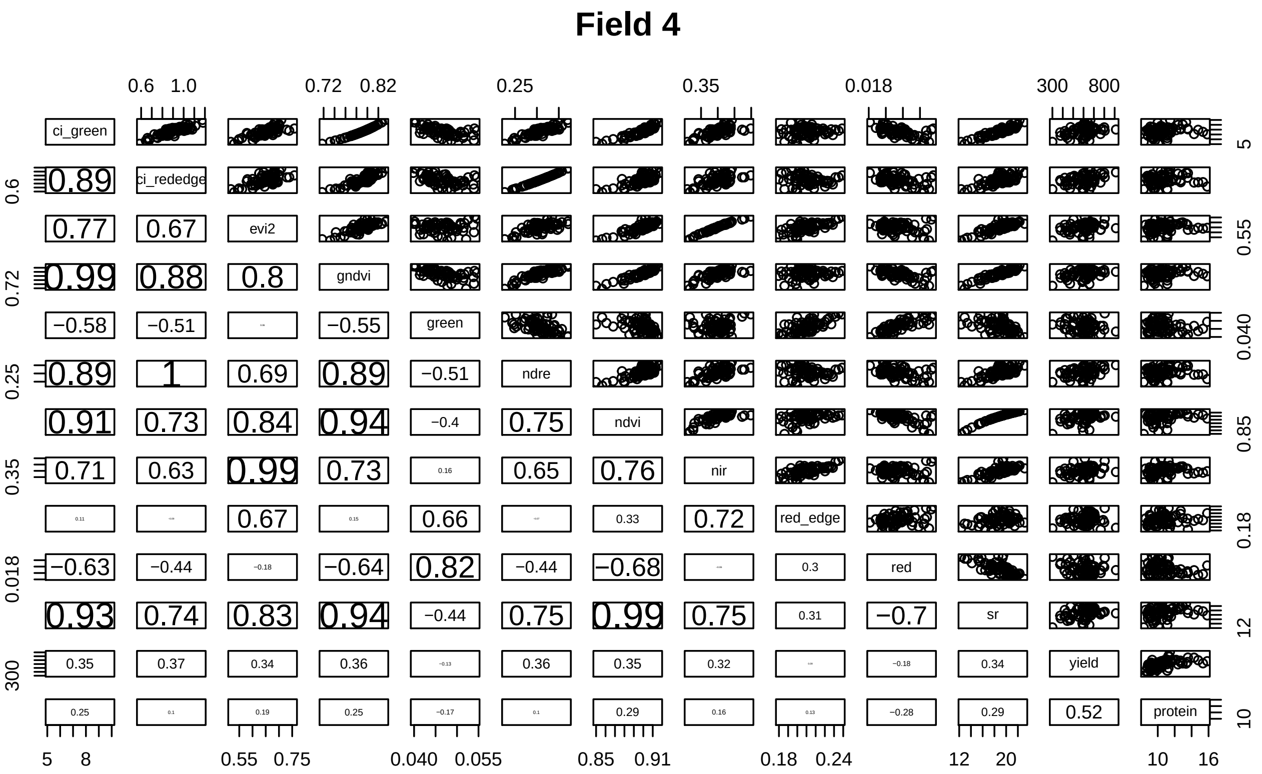

Figure S15: Scatterplots (top right) and Pearson correlations (bottom left) between variables from field 4.

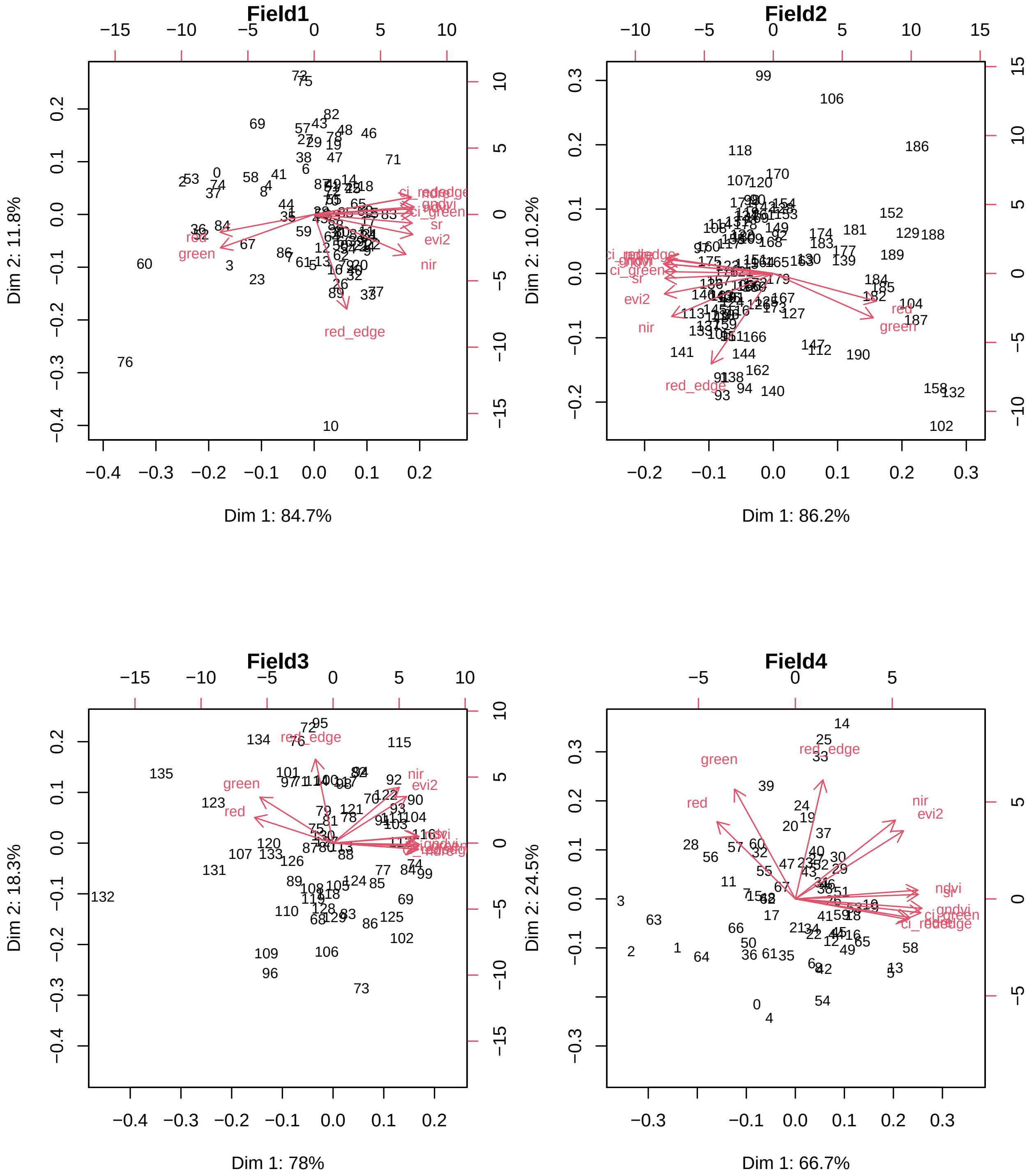

Figure S16: Biplots of PCAs of 4 different fields for the 11 regressors. Black numbers indicate plants, red arrows are the loadings of multispectral features in principal components 1 and 2. The covariance structure between the spectral variables appears similar across the fields.

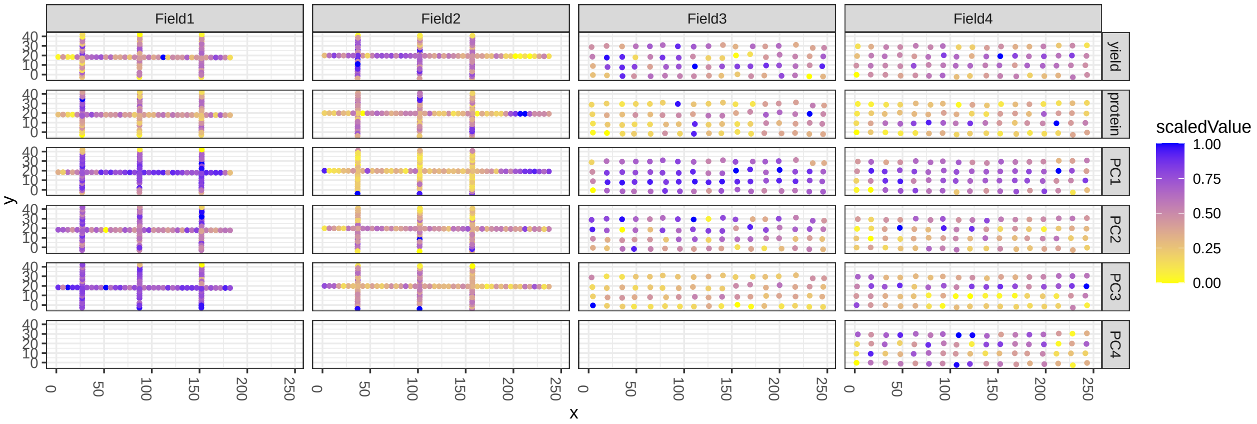

Figure S17: Heatmap of zero-one scaled principal components and yield variables (rows) for different fields (columns) for the Zhou2021 dataset. x- and y-axes are field coordinates. Only the principal components included in the analysis were plotted.

2 Results

2.1 Univariate scenario

2.1.1 Experimental studies: checkerboard design

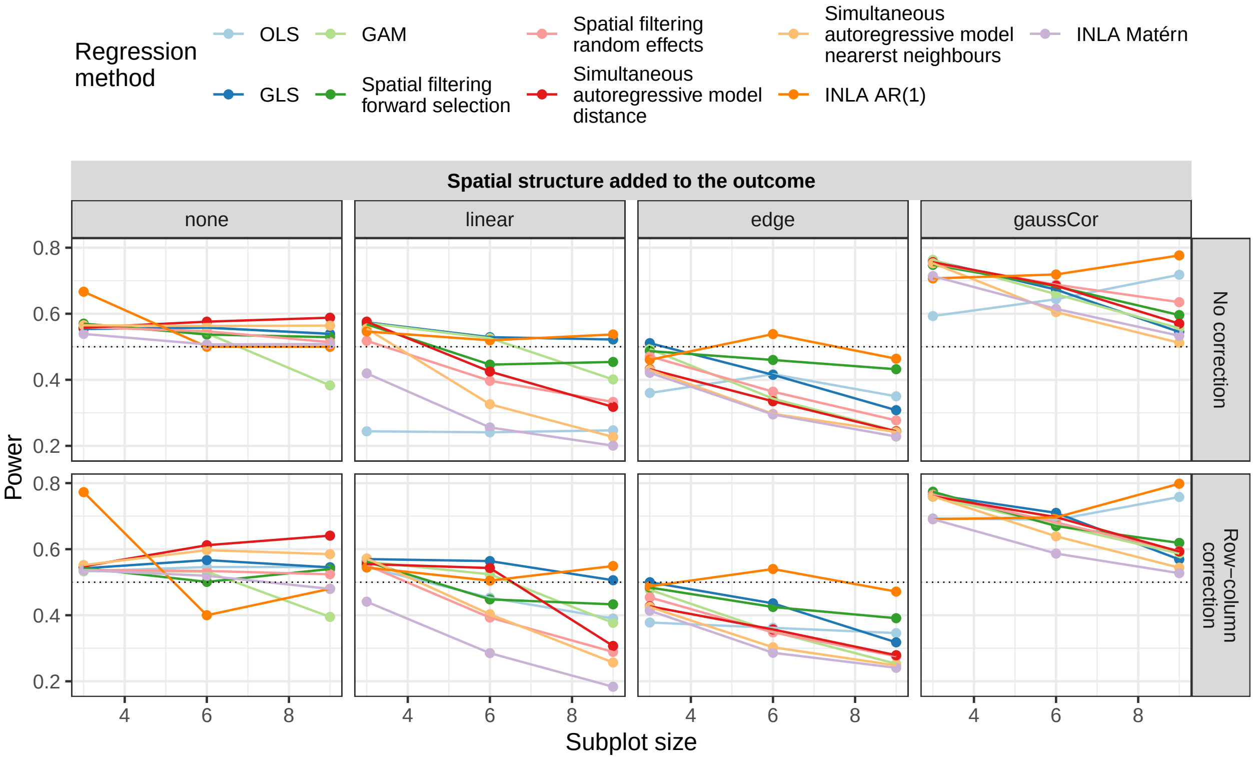

Figure S18: Power (y-axis) as a function of the size of the subplots of the checkerboard design (x-axis), of different analysis methods (colours) for different spatial structures of the outcome (columns) and depending on whether correction for row and column effects was applied (rows). The dotted horizontal line at 0.5 is purely a visual aid. GLS achieves good power in the scenarios where it controls the type I error. OLS has low power in the presence of a linear confounder, which can be remedied by correcting for row and column effects. INLA Matérn has low power overall. The power of most methods decreases with subplot size.

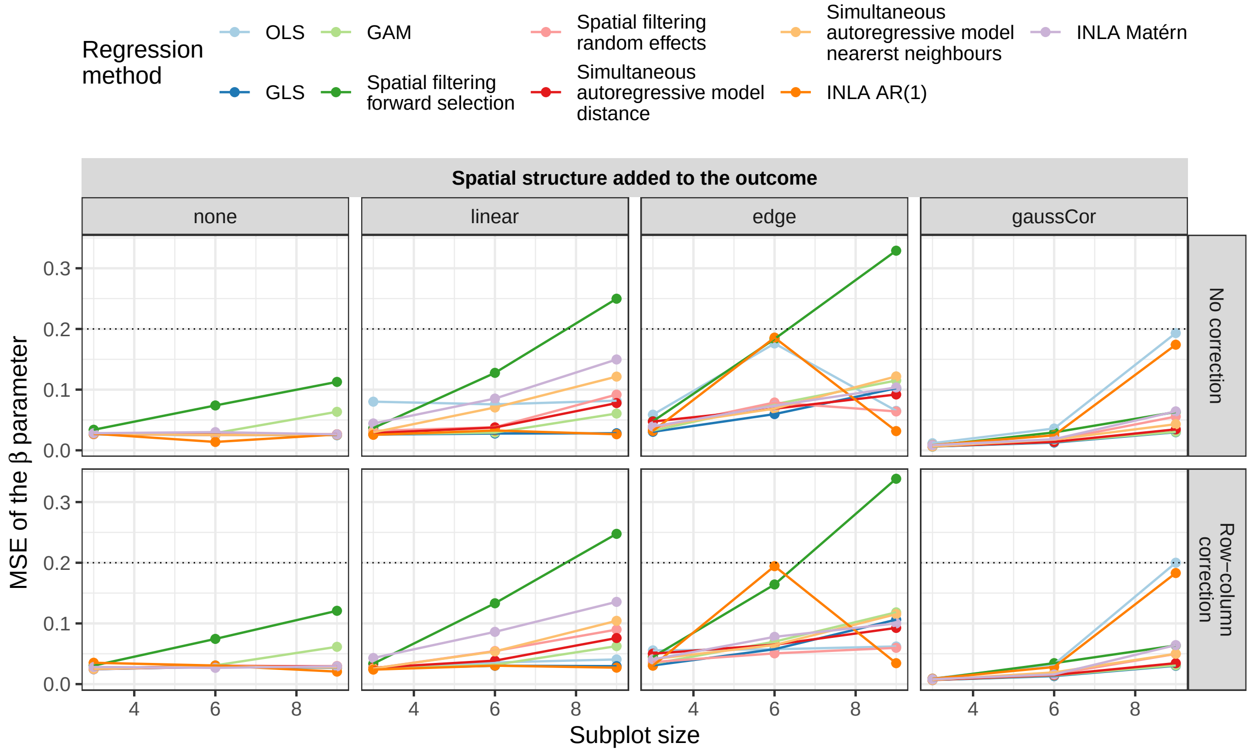

Figure S19: Average MSE of the β parameter (y-axis) as a function of the size of the subplots (x-axis), of different analysis methods (colours) for different spatial structures of the outcome (columns) and depending on whether correction for row and column effects was applied (rows). The dotted horizontal line at 0.2 is purely a visual aid. For this design, all types of spatial patterns in the outcome inflate the MSE of $\hat{\beta}$. Yet this effect can be mitigated by using smaller subplots, or by correcting for row and columns of the split plot design in case of OLS.

2.2 Low-dimensional scenario

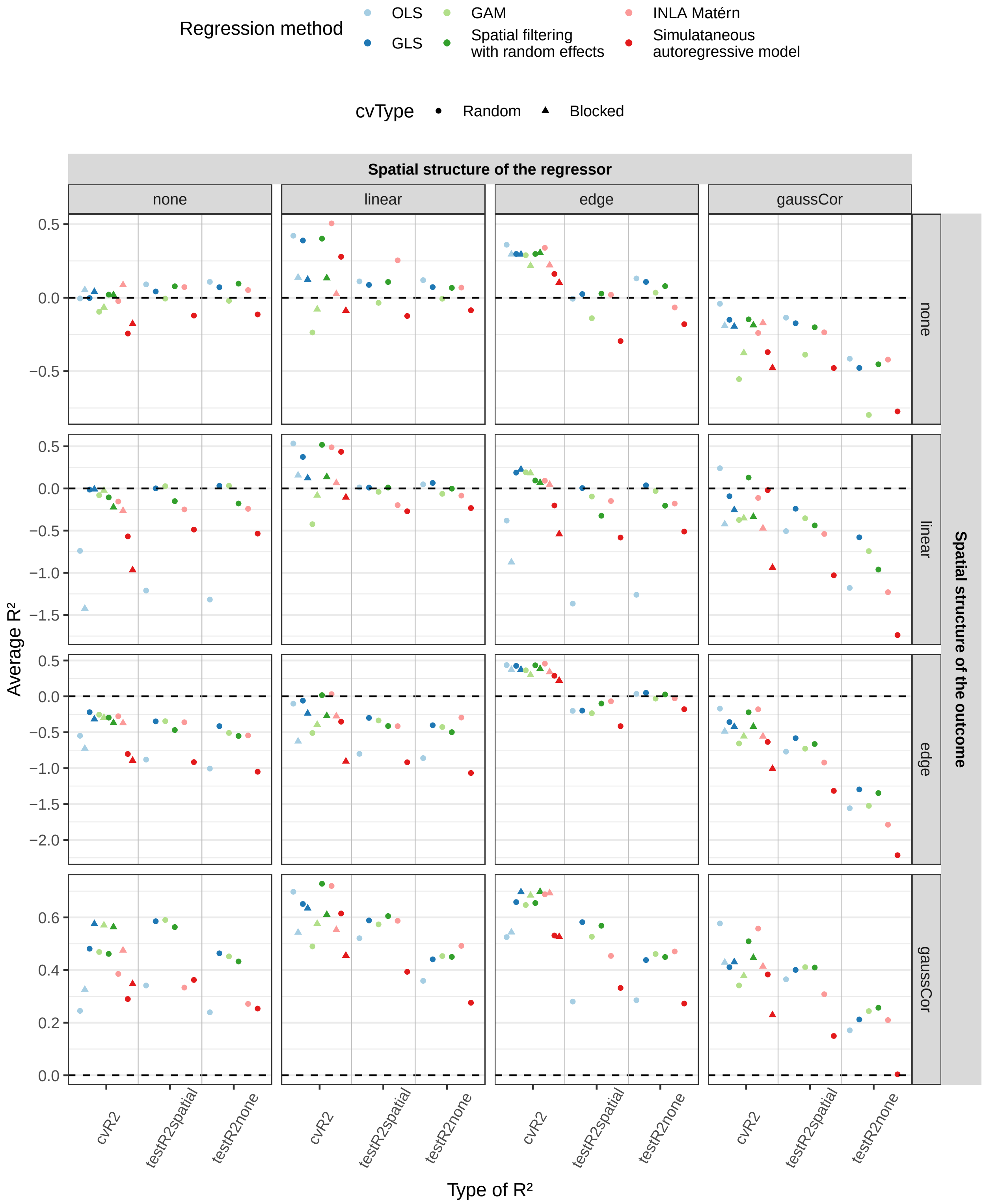

Figure S20: Average R^2 (y-axis) estimated by different cross-validation (shapes) and test datasets (x-axis) as a function of different spatial structures of the regressors (columns) and outcome (rows), and regression method (colour) for the low-dimensional scenario. 1,000 Monte Carlo runs were executed, the standard deviation of the non-zero components of β was 0.5. When there is Gaussian correlation in the regressors or outcome, the test R^2 is generally lower on observations without spatial structure (testR^2none) than on a new test field with the same spatial structure as the training data (testR^2spatial) (see Figure 4 and Supplementary Figures S20-S21)). Likely this is because SAC renders the outcomes more similar, reducing the amount of variability to be explained.

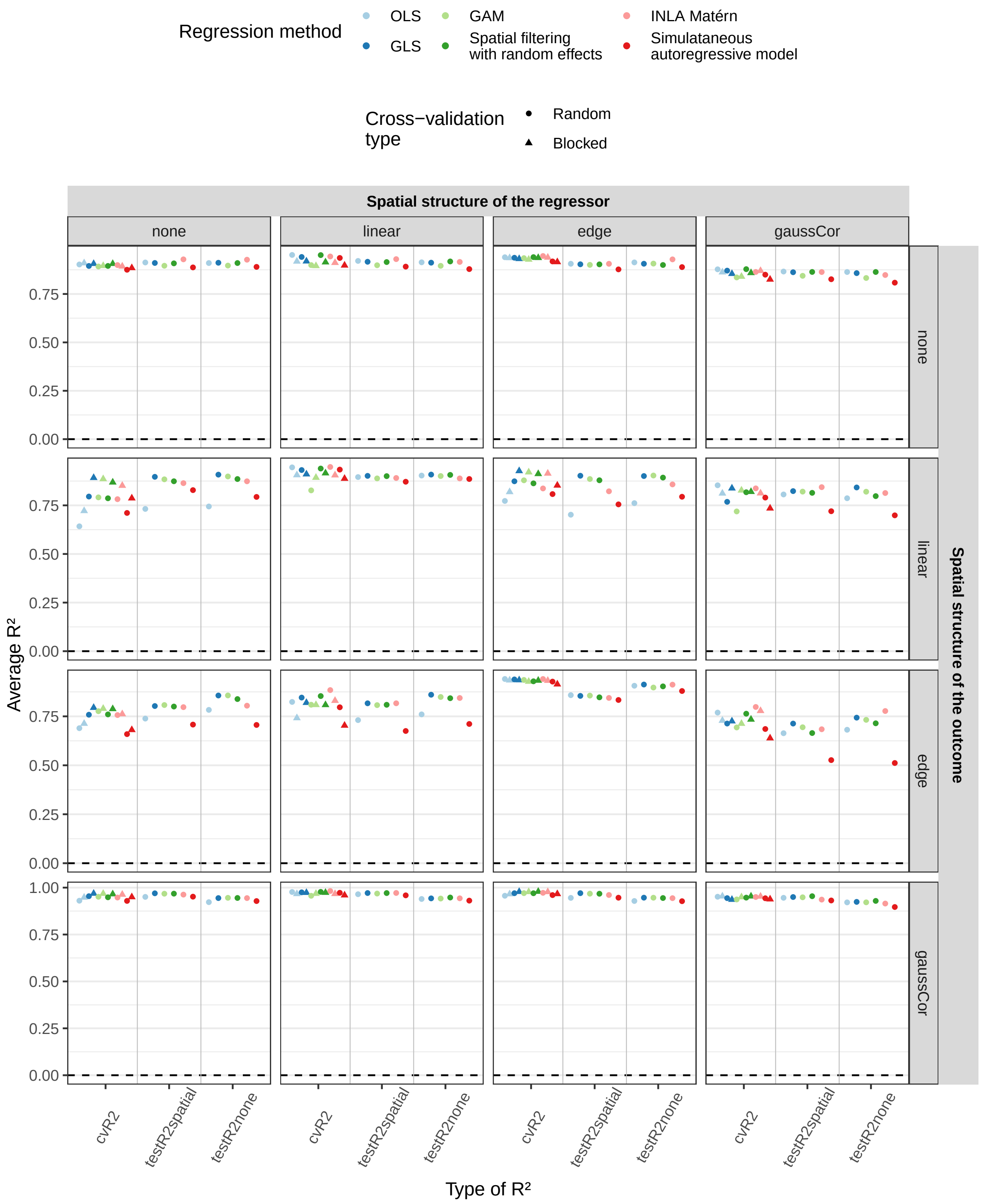

Figure S21: Average R^2 (y-axis) estimated by different cross-validation (shapes) and test datasets (x-axis) as a function of different correlation structures of the regressors (columns), regression method (colour), and spatial structure of the outcome values (rows) for the low-dimensional scenario. 1,000 Monte Carlo runs were executed, the standard deviation of the non-zero components of β was 2.

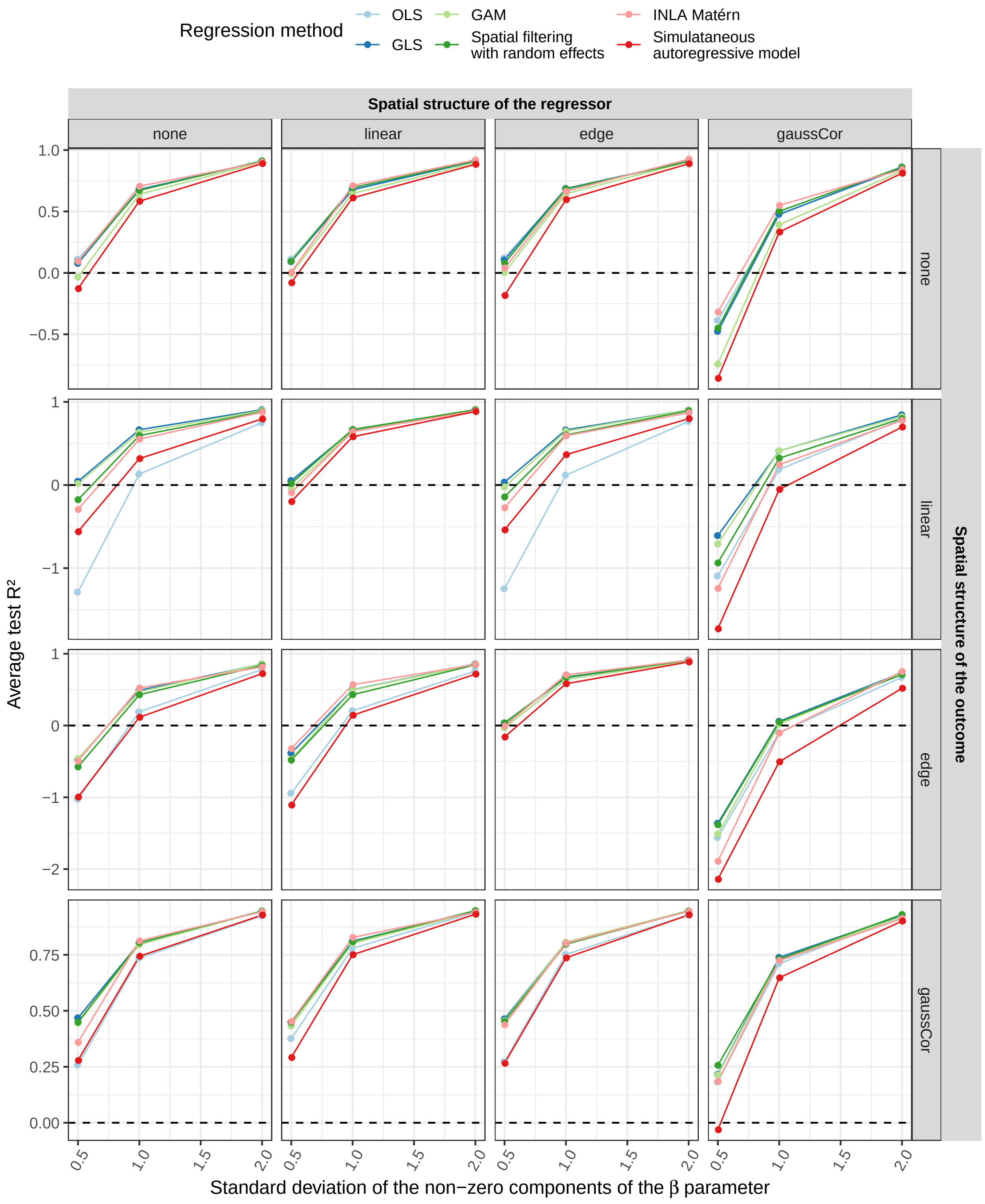

Figure S22: Average R^2 on test dataset without spatial structure (testR^2none, y-axis) as a function of regression method (colours) and standard deviation of non zero components of $\boldsymbol{\beta}$ (x-axis) for different correlation structures of the regressors (columns) and outcome values (rows) for the low-dimensional scenario. 1,000 Monte Carlo runs were executed.

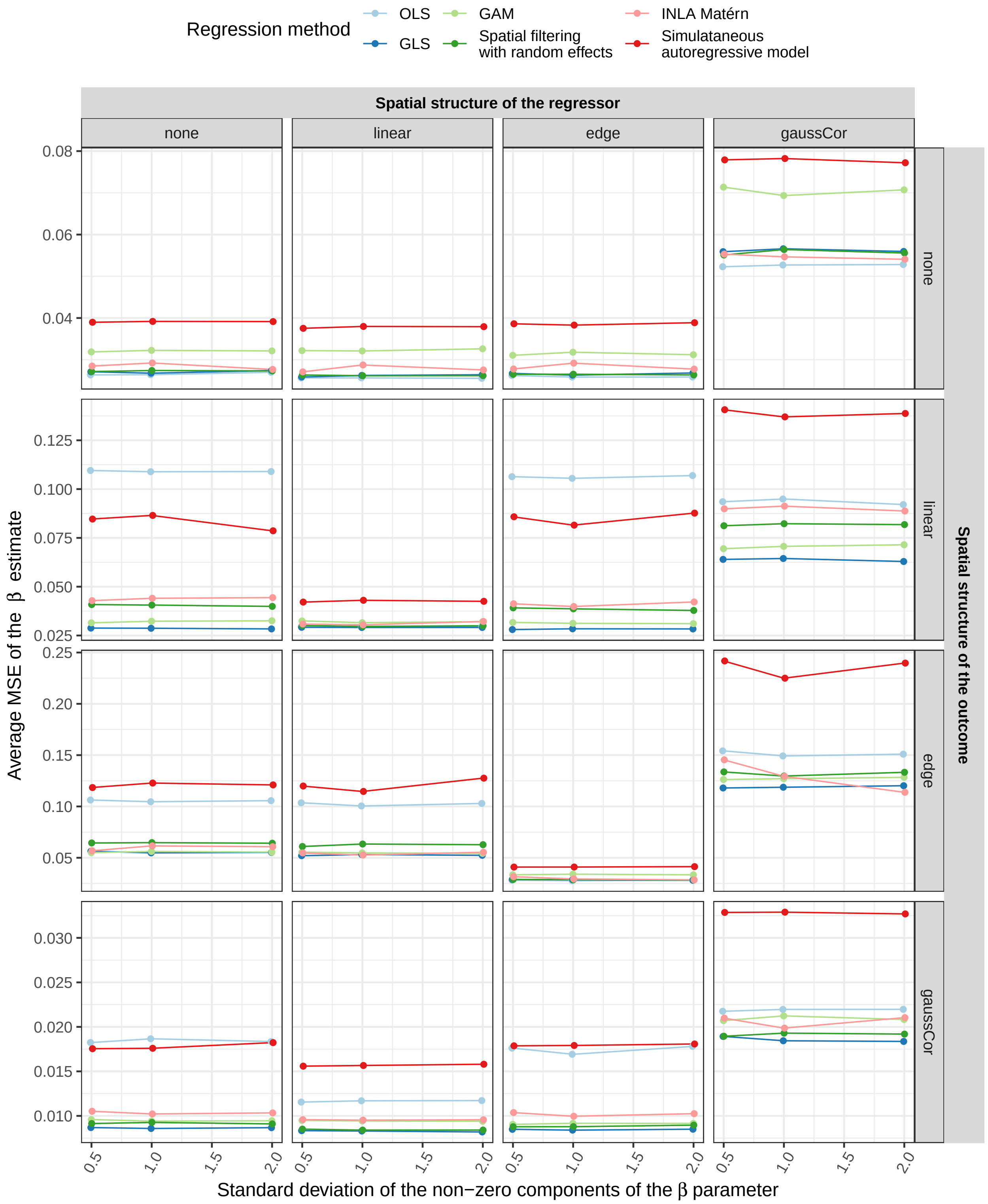

Figure S23: Average MSE of the $\boldsymbol{\beta}$ parameter (y-axis) as a function of standard deviation of the non-zero components of the $\boldsymbol{\beta}$ parameter (a measure of signal strength, x-axis) for different correlation structures of the regressors (columns) and outcome values (rows) for the low-dimensional scenario. Colours reflect regression method used. 1,000 Monte Carlo runs were executed. The differences in predictive performance are also reflected in the MSE of $\boldsymbol{\beta}$.

2.2.1 The role of the number of features

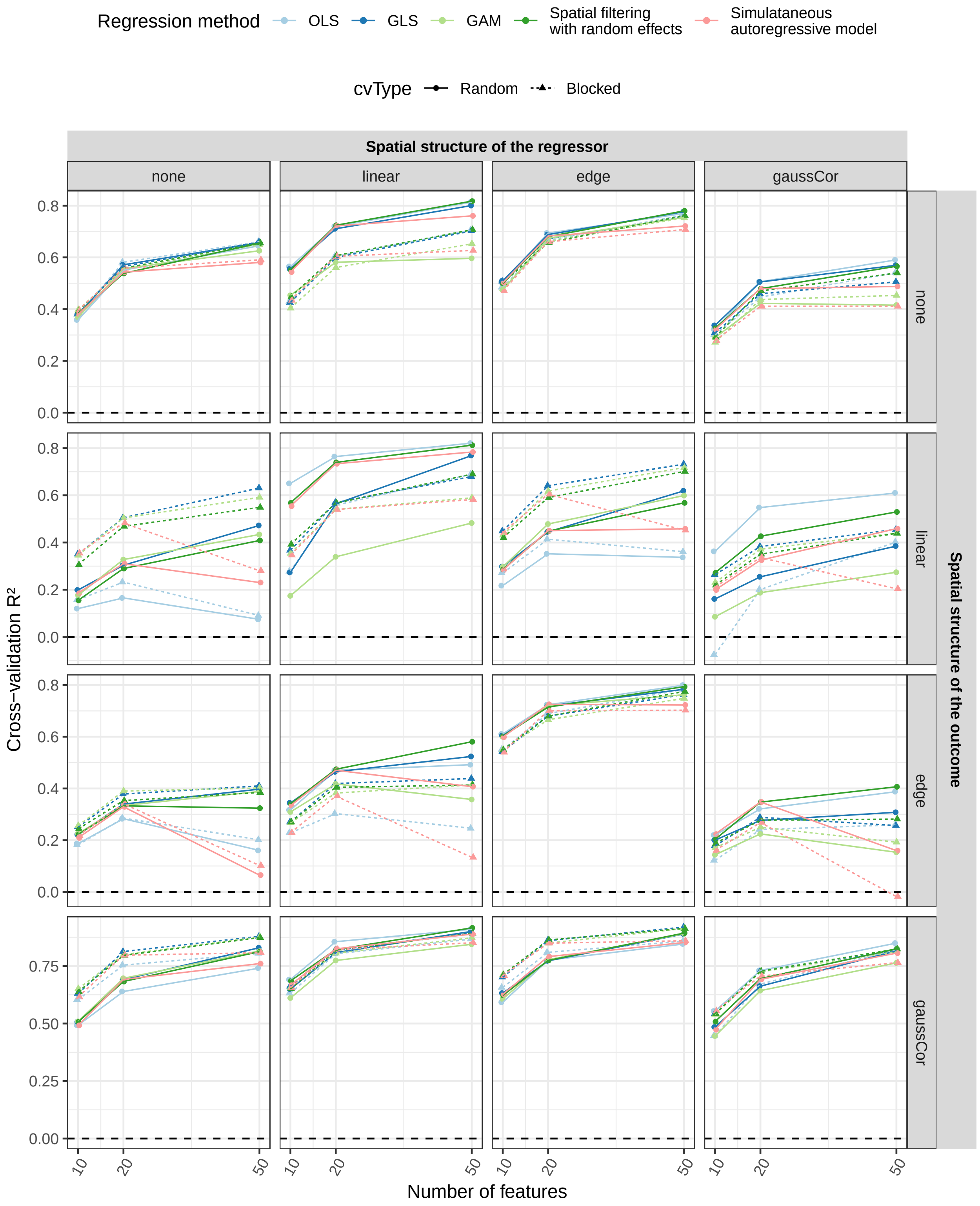

Figure S24: Cross-validation R^2 (y-axis) as a function of the number of features (x-axis) when using different cross-validation paradigms (linetype and point shape) for different regression method (colours), correlation structure of the regressors (columns) and of the outcome (rows) for the low-dimensional scenario. 1,000 Monte Carlo runs were executed, standard deviation of the non-zero components of the $\boldsymbol{\beta}$ parameter was 1.

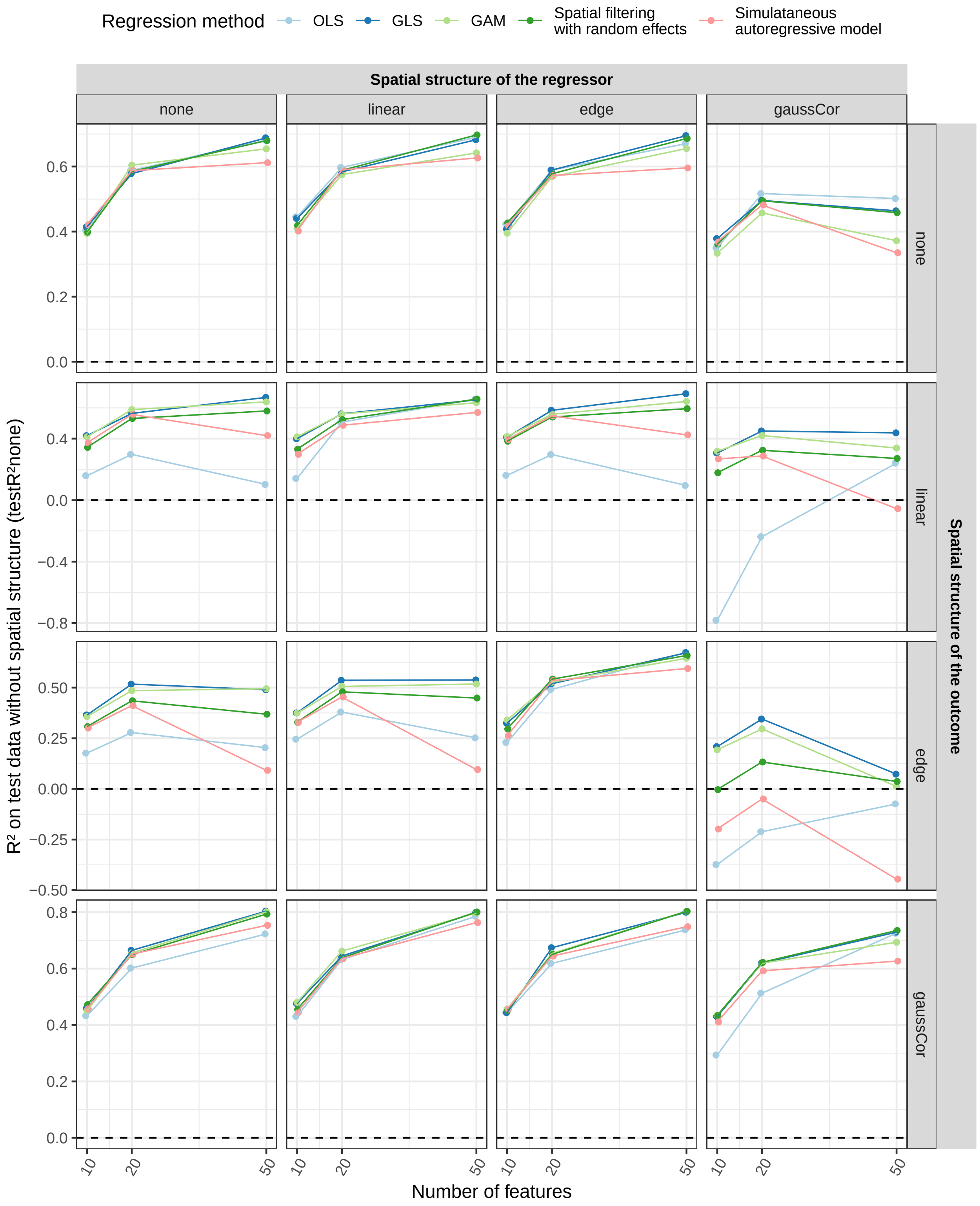

Figure S25: TestR²none (y-axis) as a function of the number of features (x-axis) and regression method (colours), correlation structure of the regressors (columns) and of the outcome (rows) for the low-dimensional scenario. 1,000 Monte Carlo runs were executed, the standard deviation of the non-zero components of the β parameter was 1.

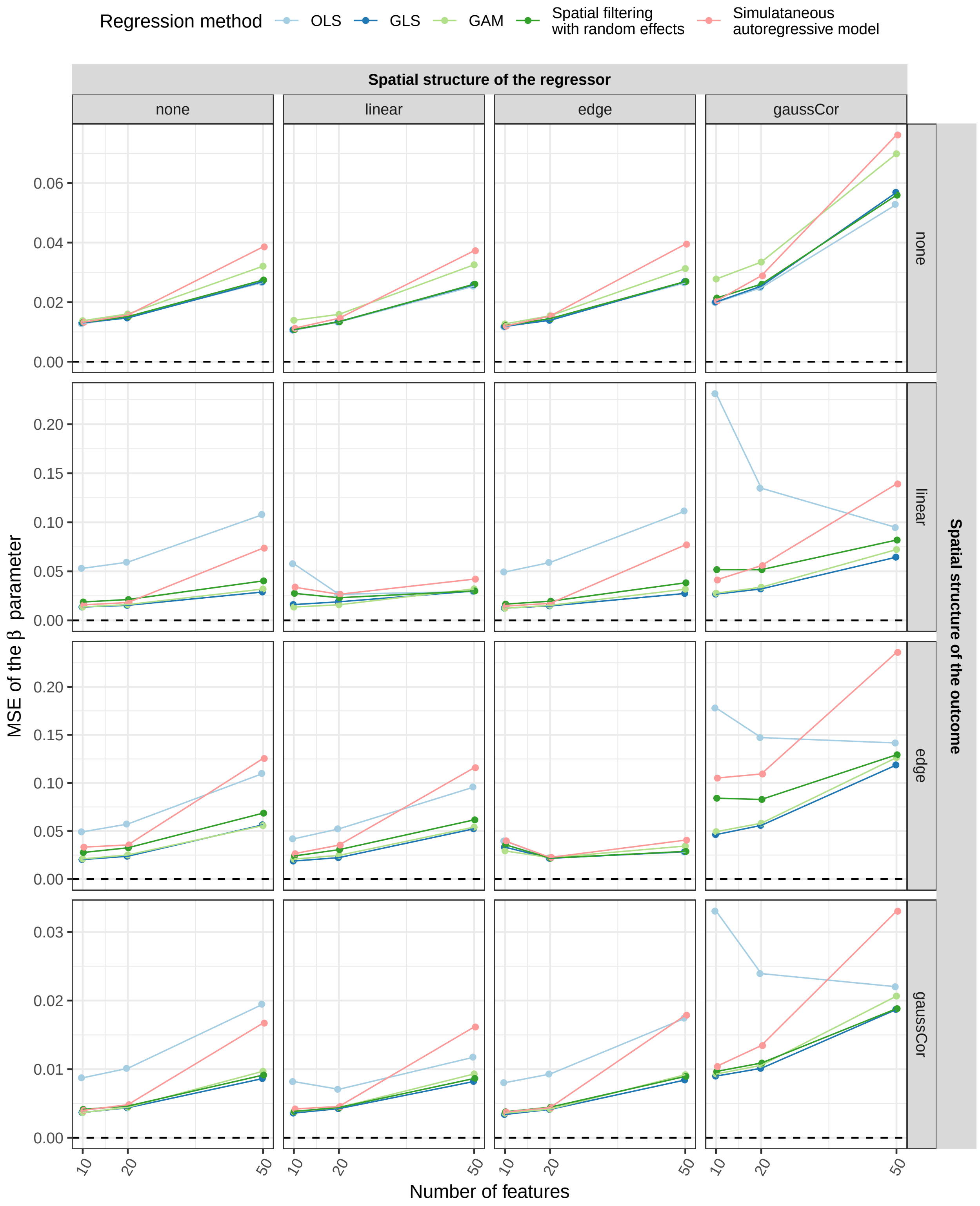

Figure S26: MSE of the $\boldsymbol{\beta}$ parameter (y-axis) as a function of the number of features (x-axis) and regression method (colours), correlation structure of the regressors (columns) and of the outcome (rows) for the low-dimensional scenario. 1,000 Monte Carlo runs were executed, standard deviation of the non-zero components of the $\boldsymbol{\beta}$ parameter was 1.

2.3 High-dimensional scenario

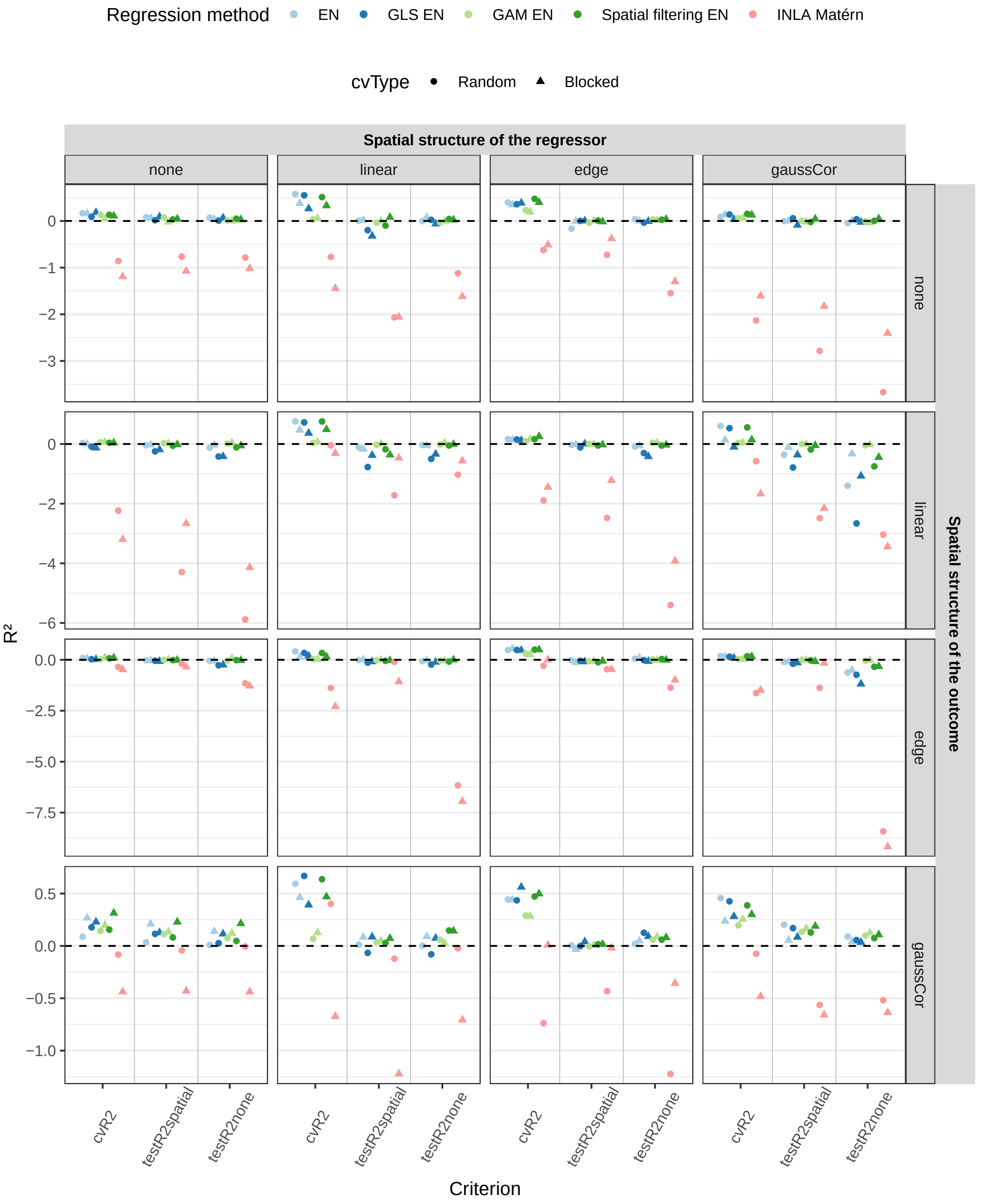

Figure S27: R² (y-axis) estimated in different ways (x-axis) when using different cross-validation paradigms (shapes) as a function of high-dimensional regression method (colours) correlation structure of the regressors (columns) and of the outcome (rows) for the high-dimensional scenario. 1,000 Monte Carlo runs were executed, standard deviation of the non-zero components of the $\boldsymbol{\beta}$ parameter was 0.25, the number of regressors was 100. As in the low-dimensional scenario, CV overestimates the R^2 on test datasets in the presence of linear trends or gaussCor in the regressors in the high-dimensional scenario, although blocked cross-validation reduces this overestimation. In case of edge effects in the regressors, also blocked CV suffers from overestimation of the R^2.

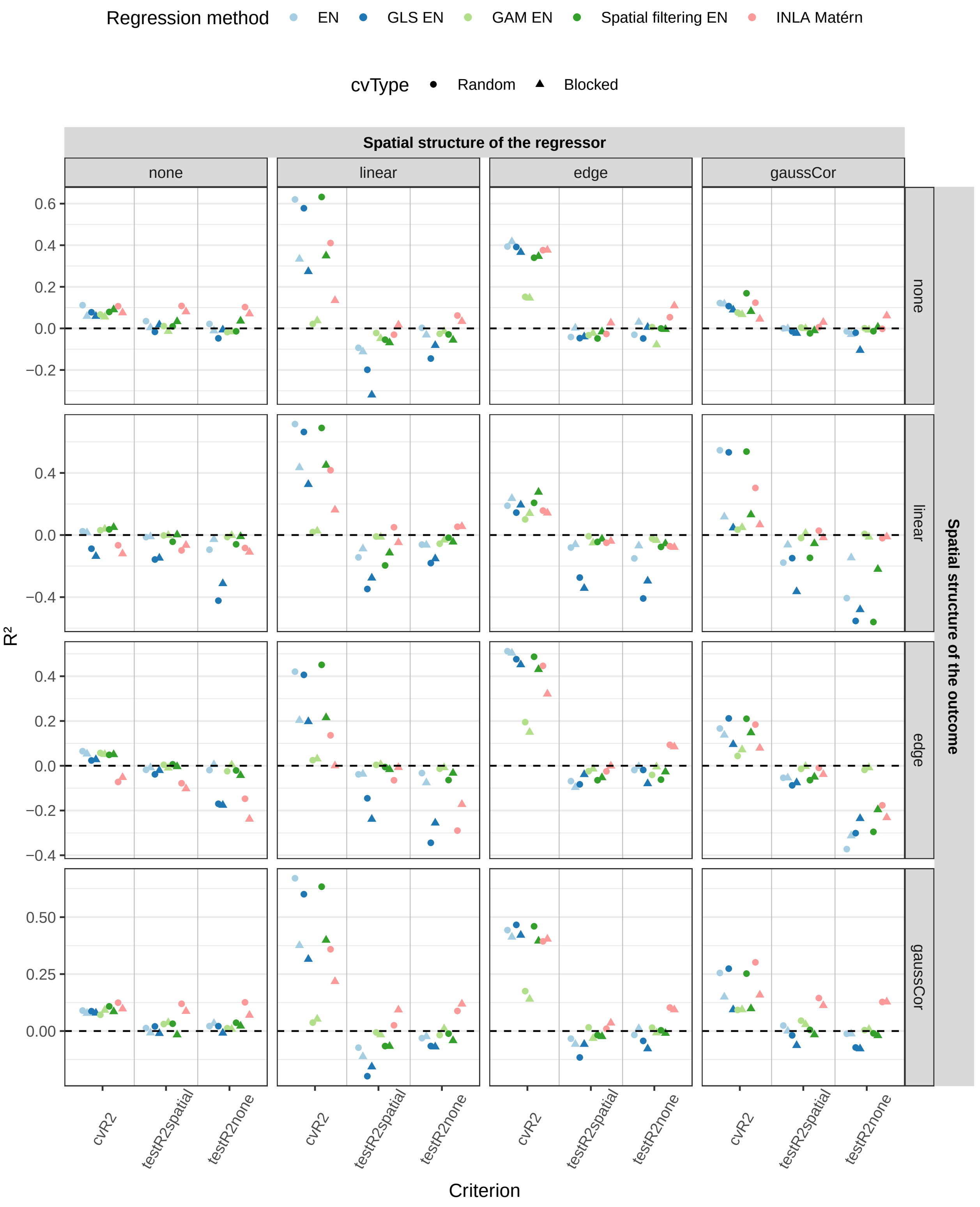

Figure S28: R^2 (y-axis) estimated in different ways (x-axis) when using different cross-validation paradigms (shapes) as a function of high-dimensional regression method (colours) correlation structure of the regressors (columns) and of the outcome (rows) for the high-dimensional scenario. 200 Monte Carlo runs were executed, standard deviation of the non-zero components of the β parameter was 0.25, the number of regressors was 300.

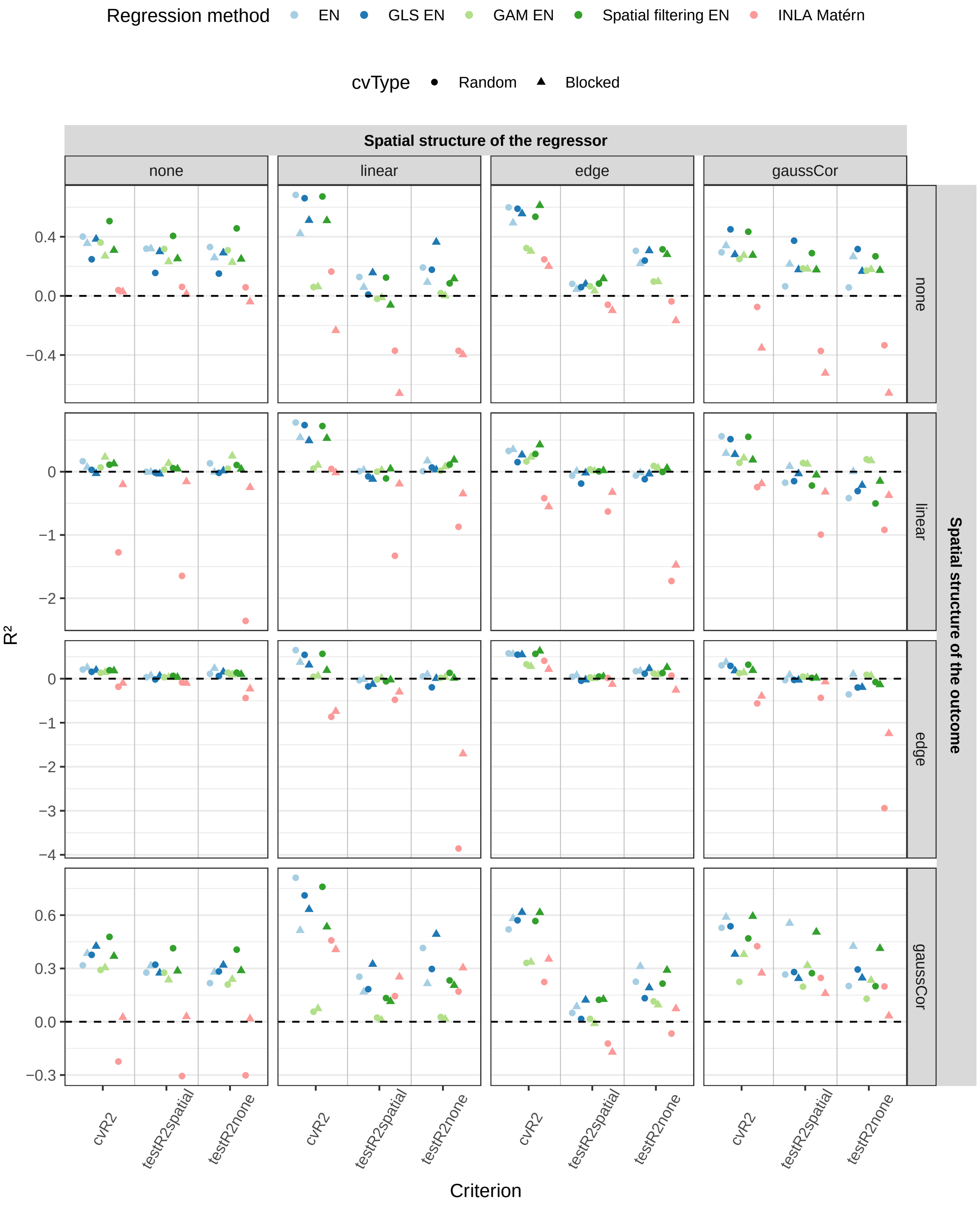

Figure S29: R^2 (y-axis) estimated in different ways (x-axis) when using different cross-validation paradigms (shapes) as a function of high-dimensional regression method (colours) correlation structure of the regressors (columns) and of the outcome (rows) for the high-dimensional scenario. 200 Monte Carlo runs were executed, standard deviation of the non-zero components of the β parameter was 0.5, the number of regressors was 100.

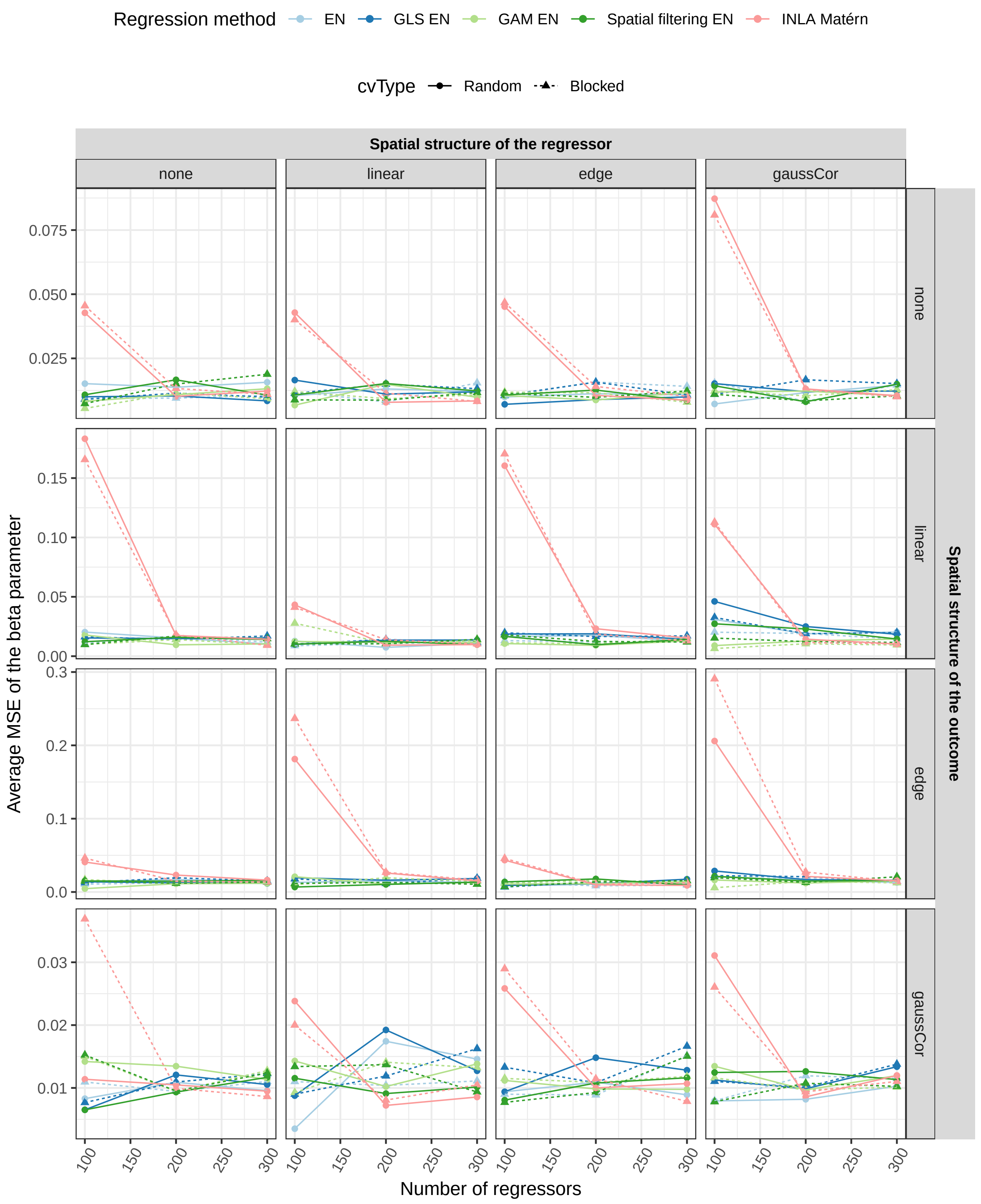

Figure S30: Average MSE of the $\boldsymbol{\beta}$ parameter (y-axis) when using different cross-validation paradigms (shapes and linetypes) as a function of the number of regressors (x-axis), high-dimensional regression method (colours) correlation structure of the regressors (columns) and of the outcome (rows) for the high-dimensional scenario. The standard deviation of the non-zero components of the $\boldsymbol{\beta}$ parameter was 0.25. 200 Monte Carlo runs were executed.

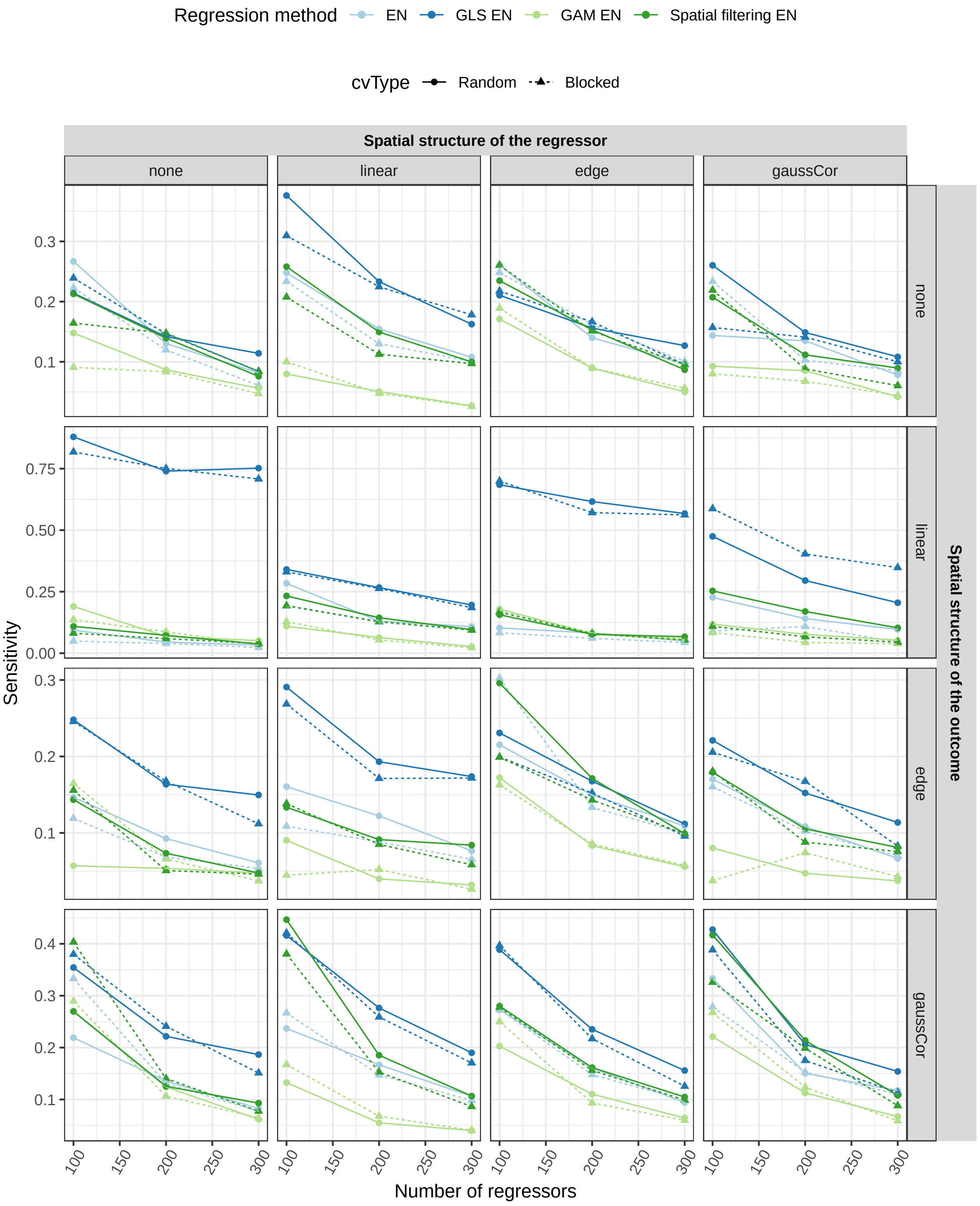

Figure S31: Sensivity (y-axis) when using different cross-validation paradigms (shapes and linetypes) as a function of number of regressors (x-axis), high-dimensional regression method (colours) correlation structure of the regressors (columns) and of the outcome (rows) for the high-dimensional scenario. The INLA Matérn model does not perform feature selection and is not included in this comparison. 200 Monte Carlo runs were executed, standard deviation of the non-zero components of the β parameter was 0.25.

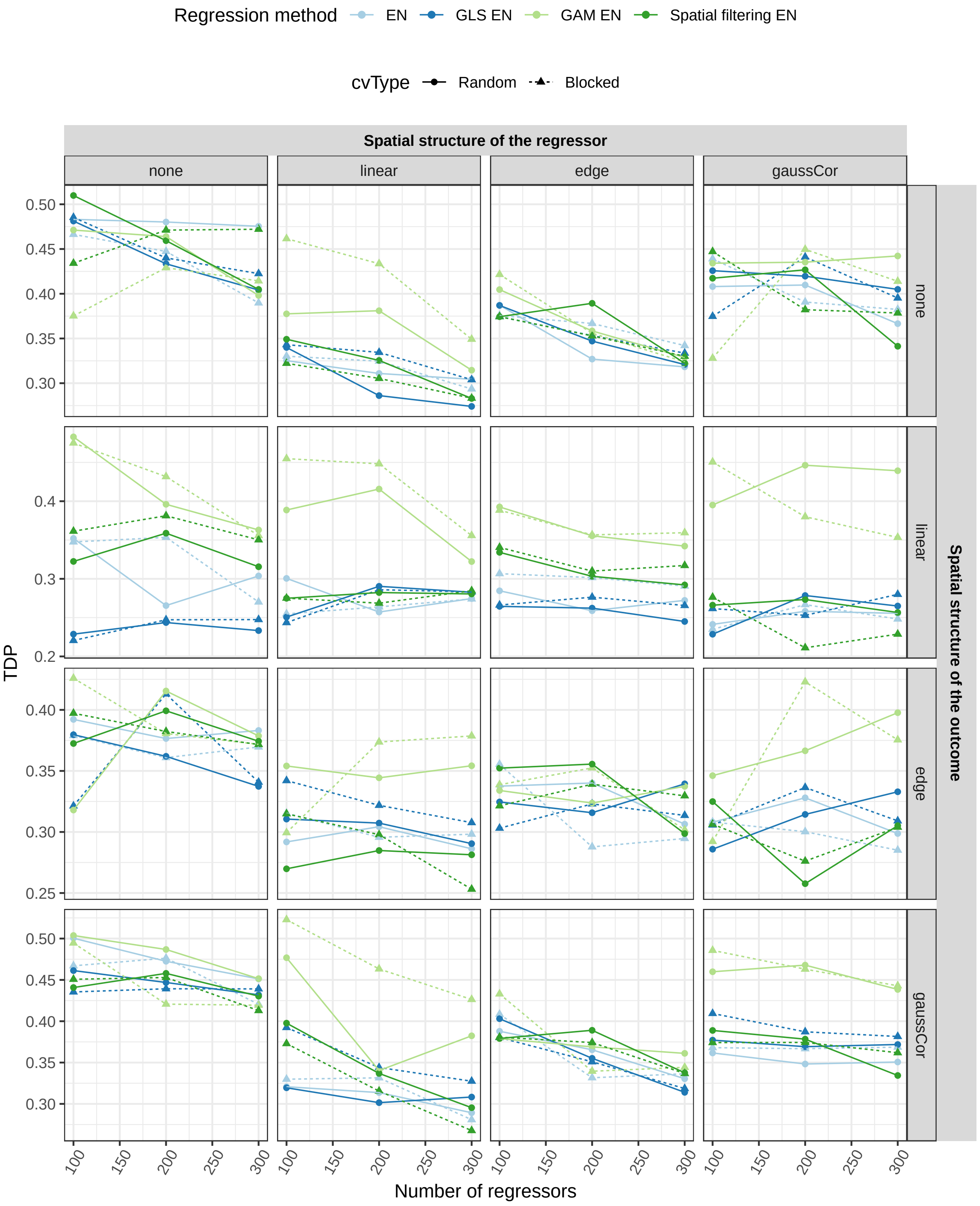

Figure S32: TDP (y-axis) when using different cross-validation paradigms (shapes and linetypes) as a function of number of regressors (x-axis), high-dimensional regression method (colours) correlation structure of the regressors (columns) and of the outcome (rows) for the high-dimensional scenario. The INLA Matérn model does not perform feature selection and is not included in this comparison. 200 Monte Carlo runs were executed, standard deviation of the non-zero components of the β parameter was 0.25.

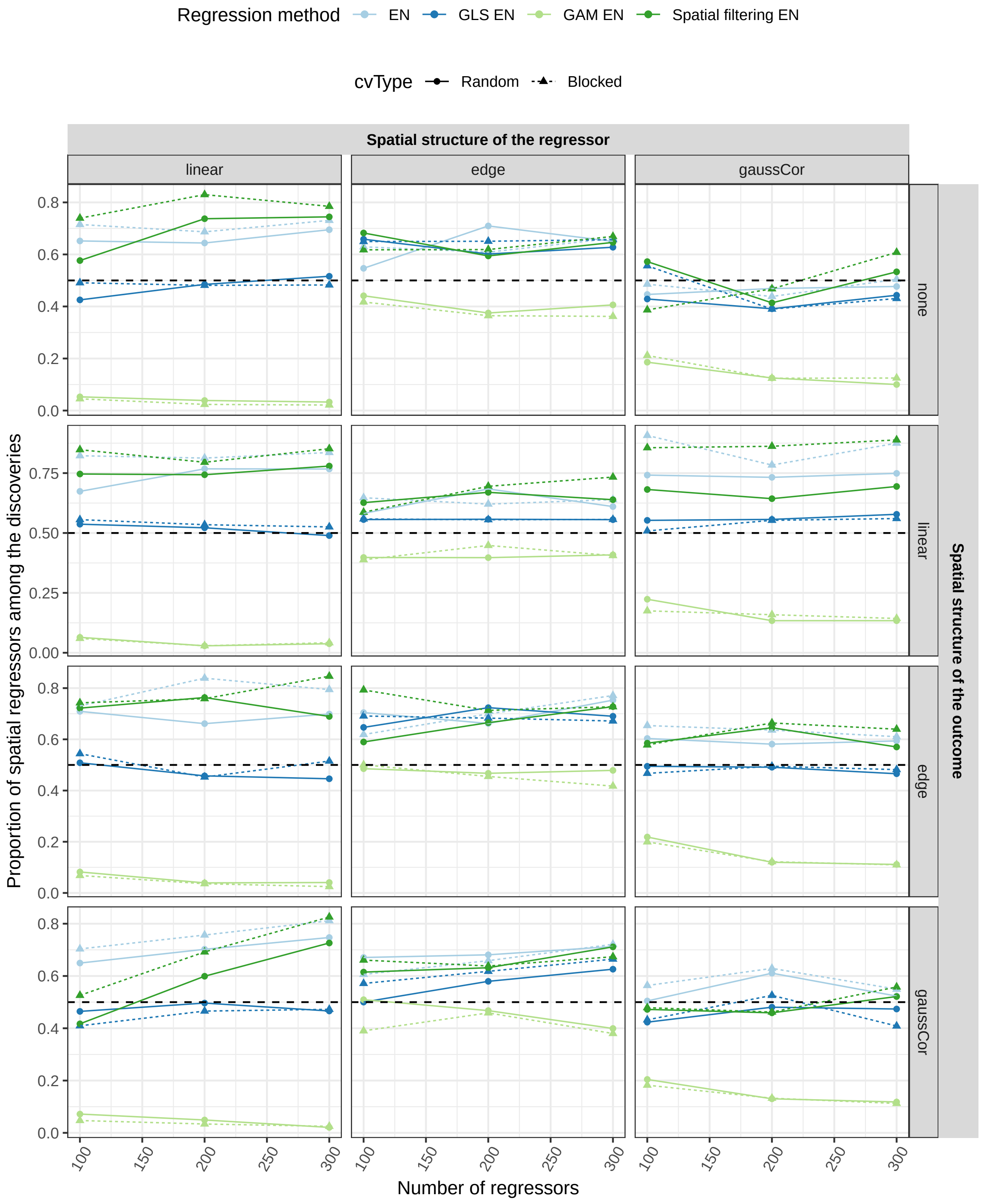

Figure S33: Average proportion of regressors with spatial signal among selected features (y-axis) when using different cross-validation paradigms (shapes and linetypes) as a function of number of regressors (x-axis), high-dimensional regression method (colours) correlation structure of the regressors (columns) and of the outcome (rows) for the high-dimensional scenario. The INLA Matérn model does not perform feature selection and is not included in this comparison. The standard deviation of the non-zero components of the $\boldsymbol{\beta}$ parameter was 0.25. The dashed line represents the expected proportion of 50% when there is no preference for spatial features.

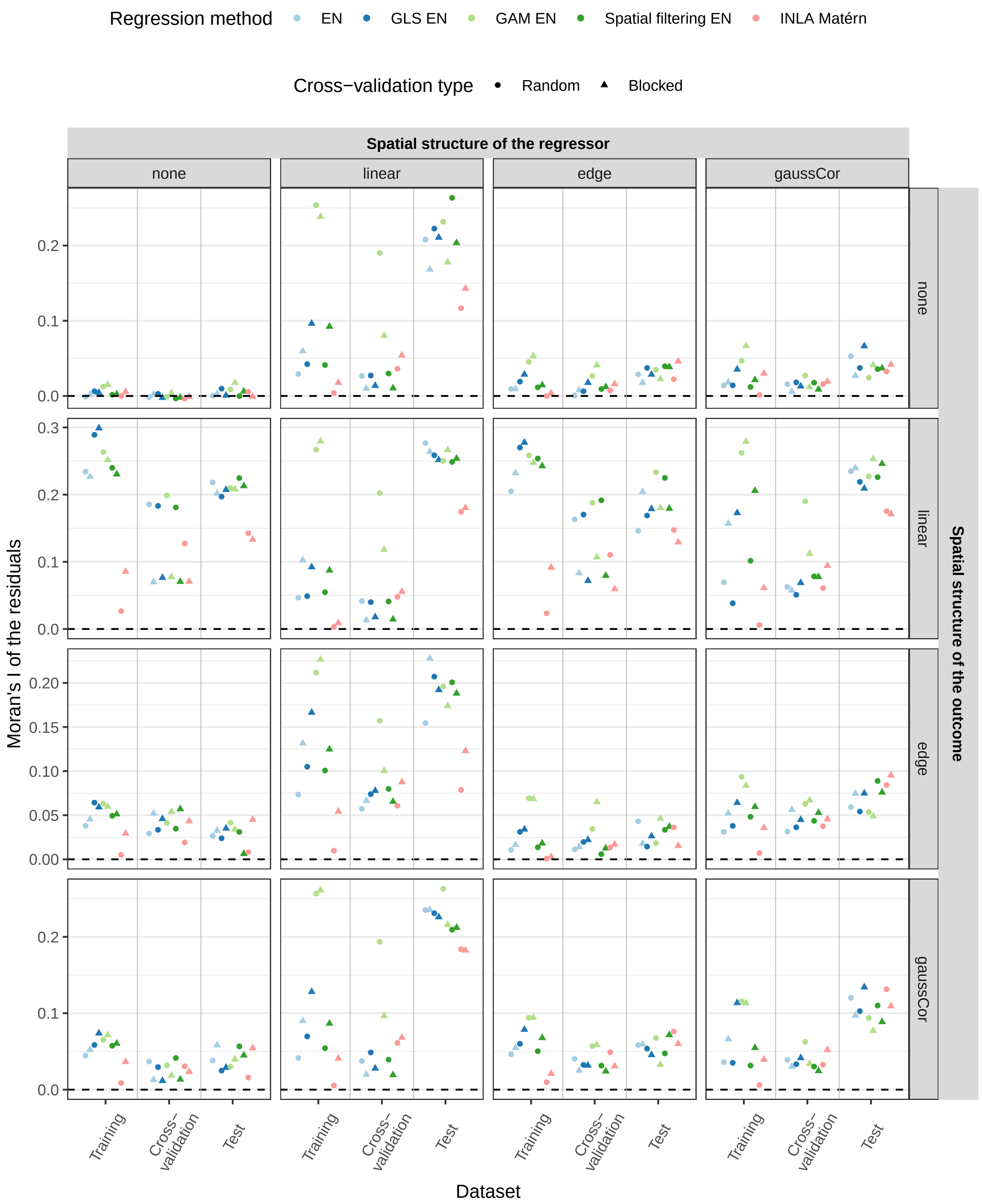

Figure S34: Average Moran's I statistic of the residuals (y-axis) of the training, cross-validation and test data (x-axis) when using different cross-validation paradigms (shapes) as a function of high-dimensional regression method (colours), correlation structure of the regressors (columns) and of the outcome (rows) for the high-dimensional scenario. The horizontal dashed line indicates the expected value in absence of spatial autocorrelation. The standard deviation of the components of β was 0.25, the number of features was 200.

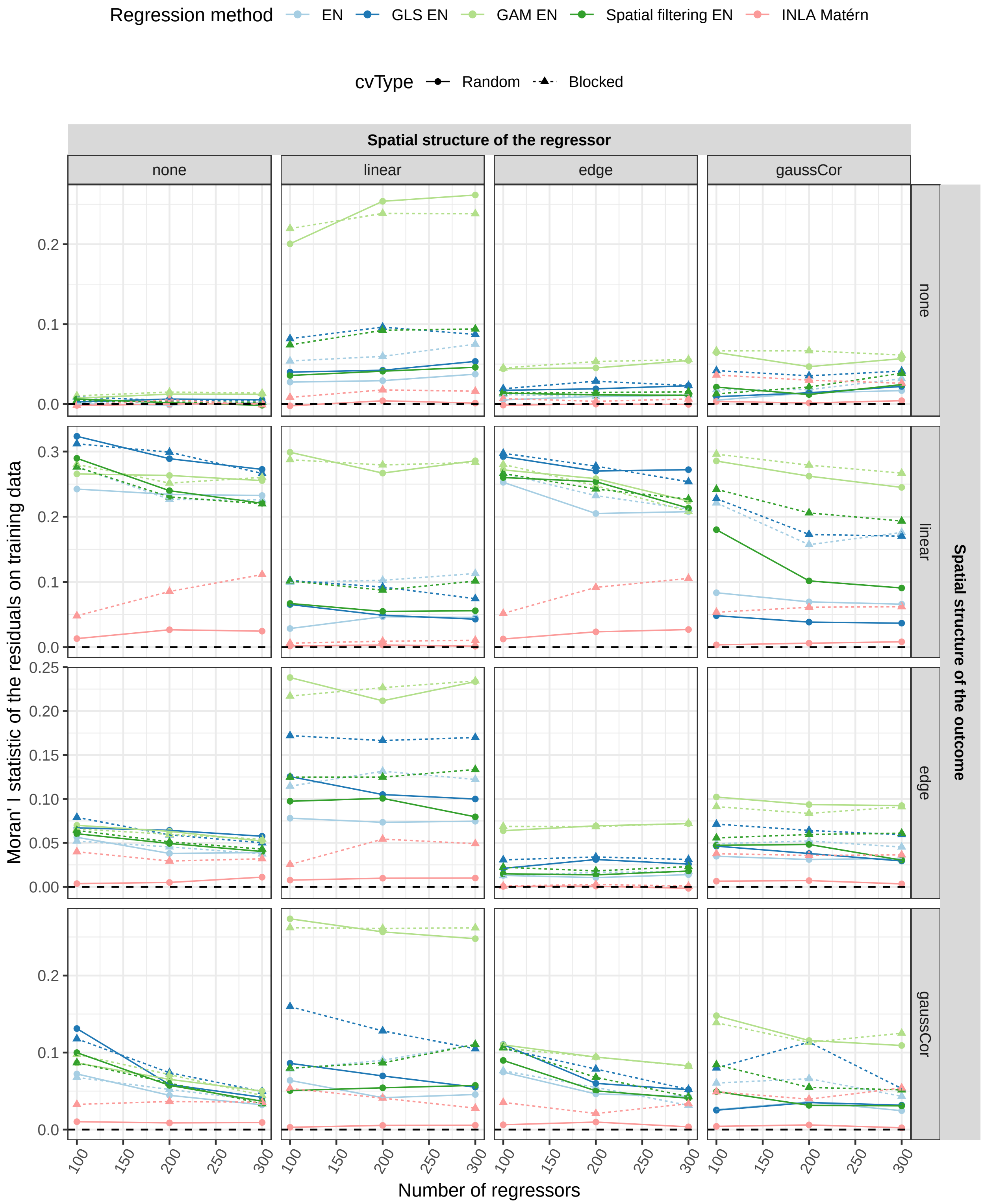

Figure S35: Average Moran's I statistic of the residuals of the training data (y-axis) when using different cross-validation paradigms (shapes and linetypes) as a function of number of features (a measure of effect strength, x-axis), high-dimensional regression method (colours) correlation structure of the regressors (columns) and of the outcome (rows) for the high-dimensional scenario. The standard deviation of the non-zero components of the $\boldsymbol{\beta}$ parameter was 0.25. The horizontal dashed line indicates the expected value in absence of spatial autocorrelation.

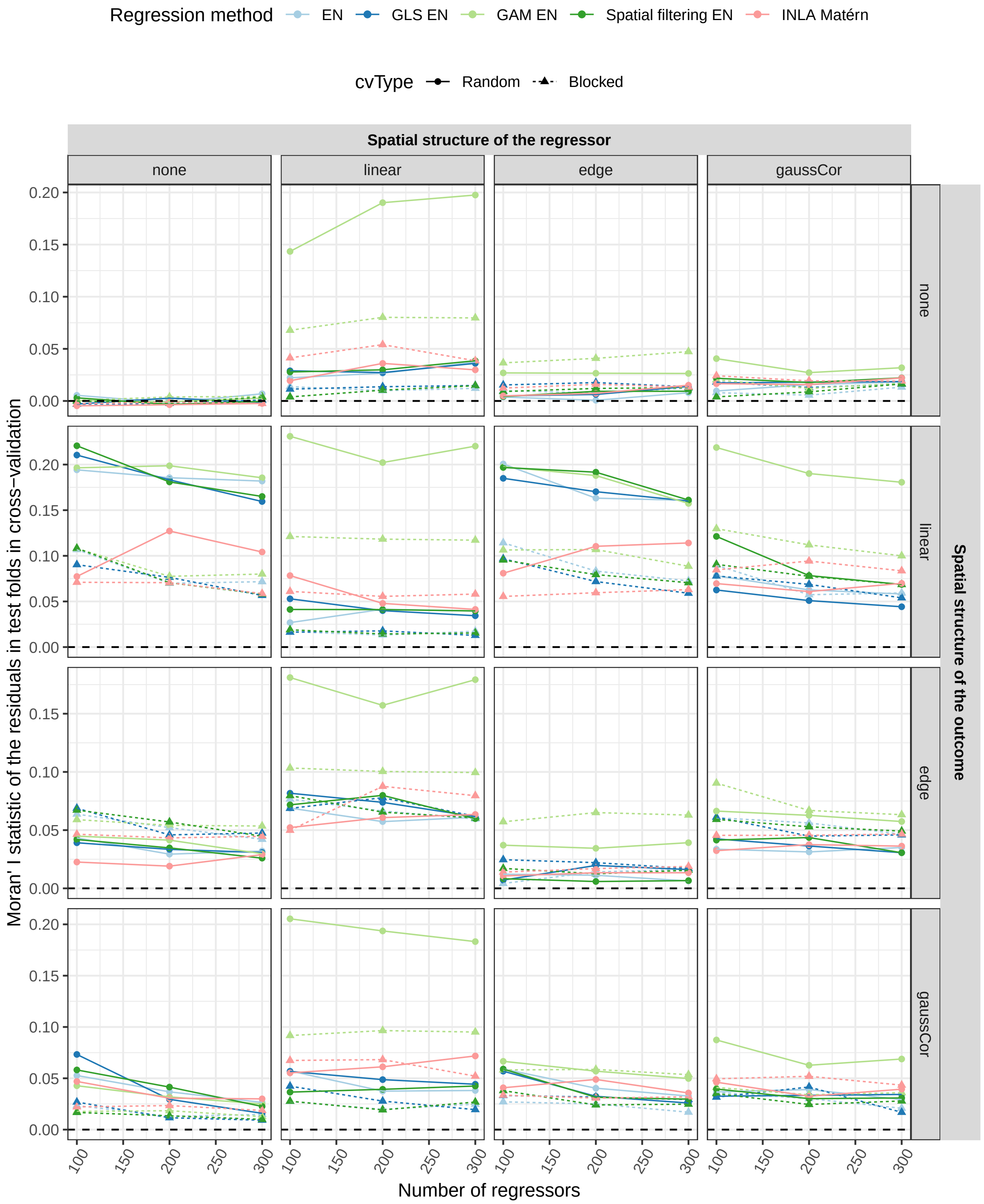

Figure S36: Average Moran's I statistic of the residuals in cross-validation (y-axis) when using different cross-validation paradigms (shapes and linetypes) as a function of number of features (a measure of effect strength, x-axis), high-dimensional regression method (colours) correlation structure of the regressors (columns) and of the outcome (rows) for the high-dimensional scenario. The standard deviation of the non-zero components of the $\boldsymbol{\beta}$ parameter was 0.25. The horizontal dashed line indicates the expected value in absence of spatial autocorrelation.

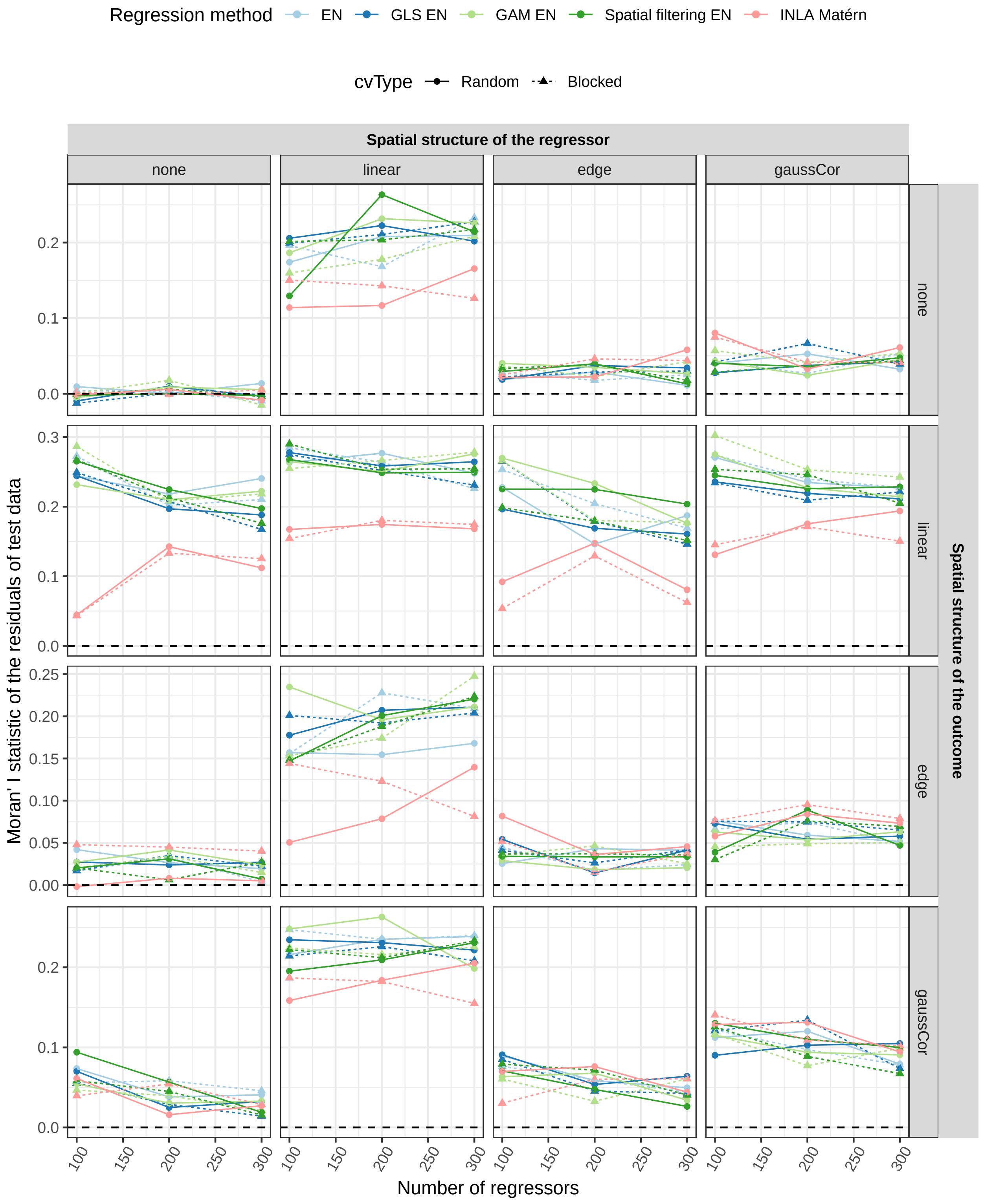

Figure S37: Average Moran's I statistic of the residuals of test data (y-axis) when using different cross-validation paradigms (shapes and linetypes) as a function of number of features (a measure of effect strength, x-axis), high-dimensional regression method (colours) correlation structure of the regressors (columns) and of the outcome (rows) for the high-dimensional scenario. The standard deviation of the non-zero components of the $\boldsymbol{\beta}$ parameter was 0.25. The horizontal dashed line indicates the expected value in absence of spatial autocorrelation.

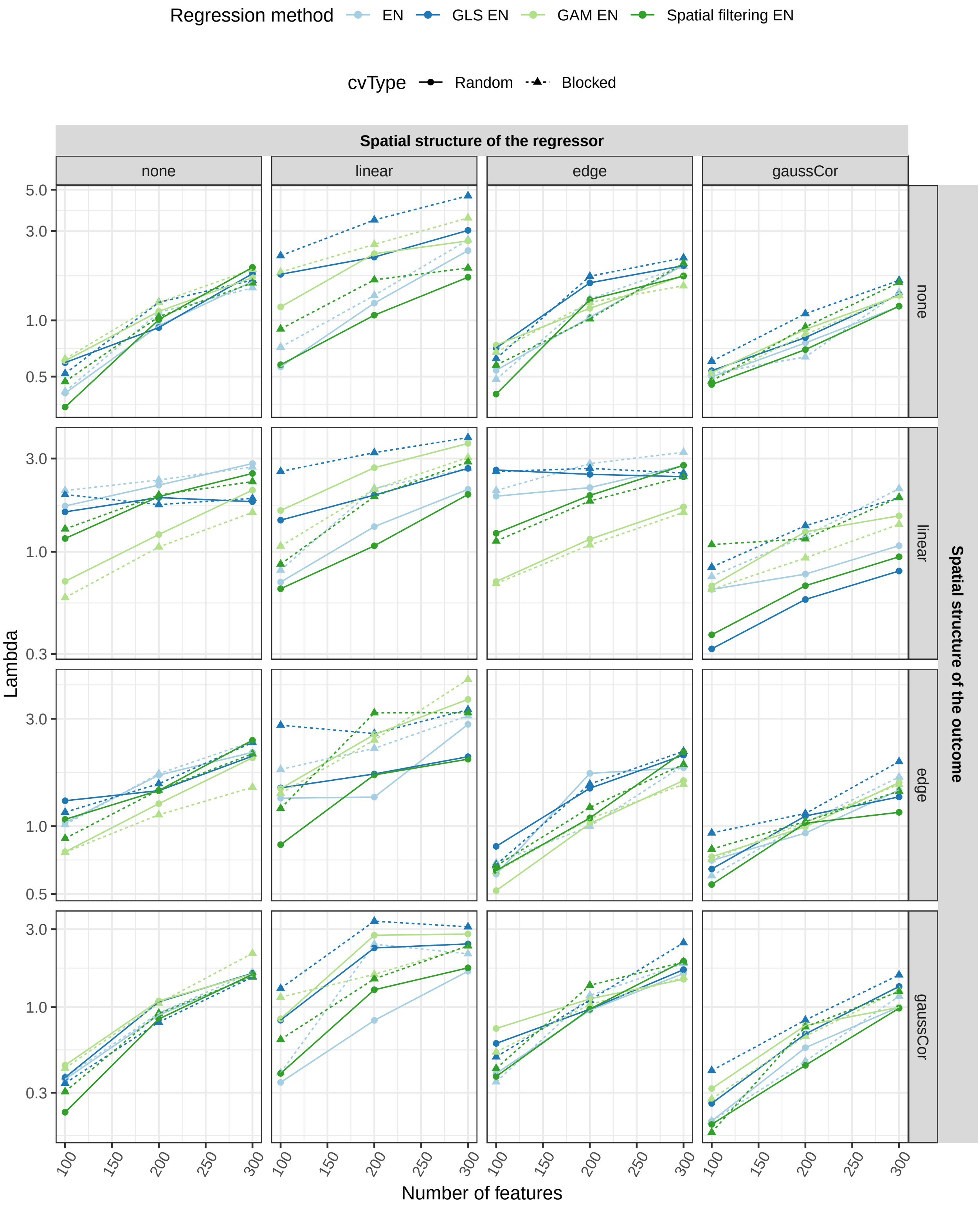

Figure S38: Lambda penalty parameter (y-axis) as a afunction of number of features (x-axis) when using different cross-validation paradigms (linetype) as a function of regression method (colours), correlation structure of the regressors (columns) and of the outcome (rows) for the high-dimensional scenario. The standard deviation of the components of the β was 0.25. 200 Monte Carlo runs were executed. No effect of the cross-validation paradigm on the penalty parameter can be seen.

2.4 Case study

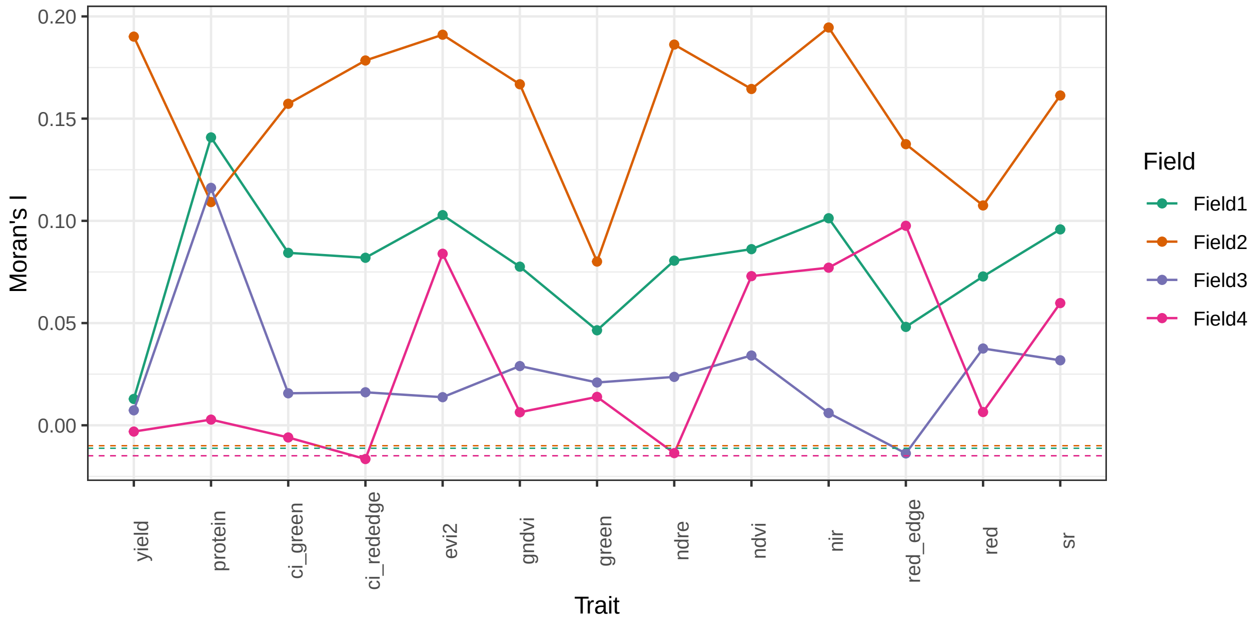

Figure S39: Moran's I statistic (y-axis) for different variables (x-axis) and fields (colours). The coloured dashed line represent corresponding expected values of the Moran's I statistic in absence of spatial autocorrelation.

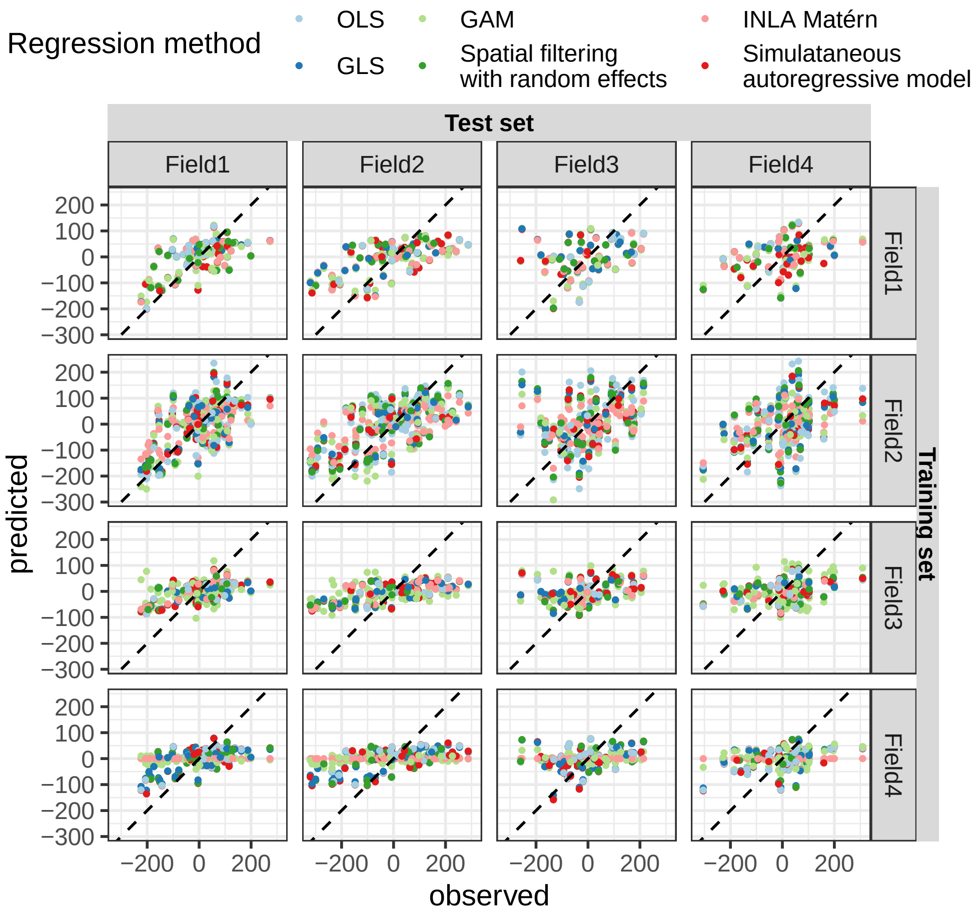

Figure S40: Scatterplot of predicted versus observed yield (both are mean centered), for different regression methods (colours) for different test and training fields (columns and rows respectively). The diagonal dashed line indicates perfect prediction.

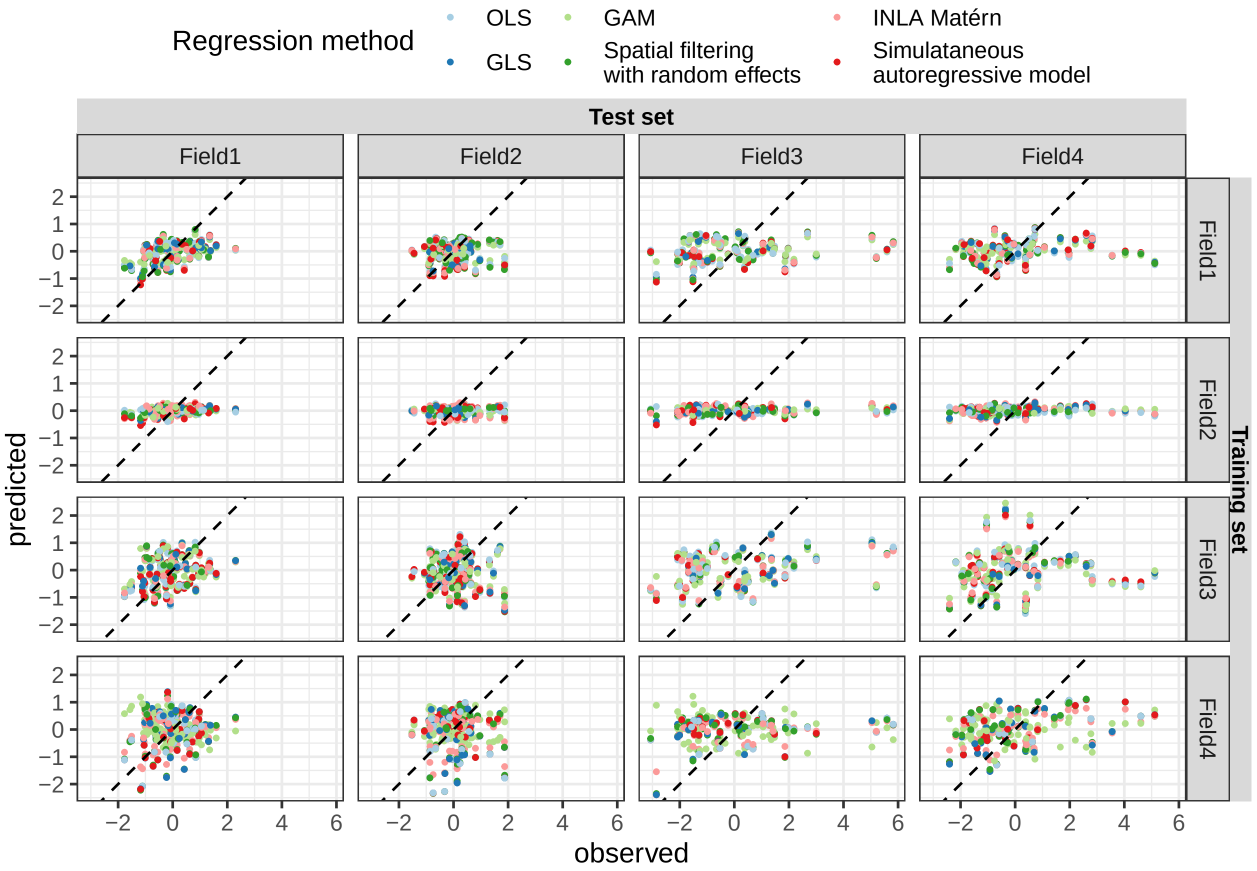

Figure S41: Scatterplot of predicted versus observed protein content (both are mean centered), for different regression methods (colours) for different test and training fields (top and right respectively). The diagonal dashed line indicates perfect prediction.

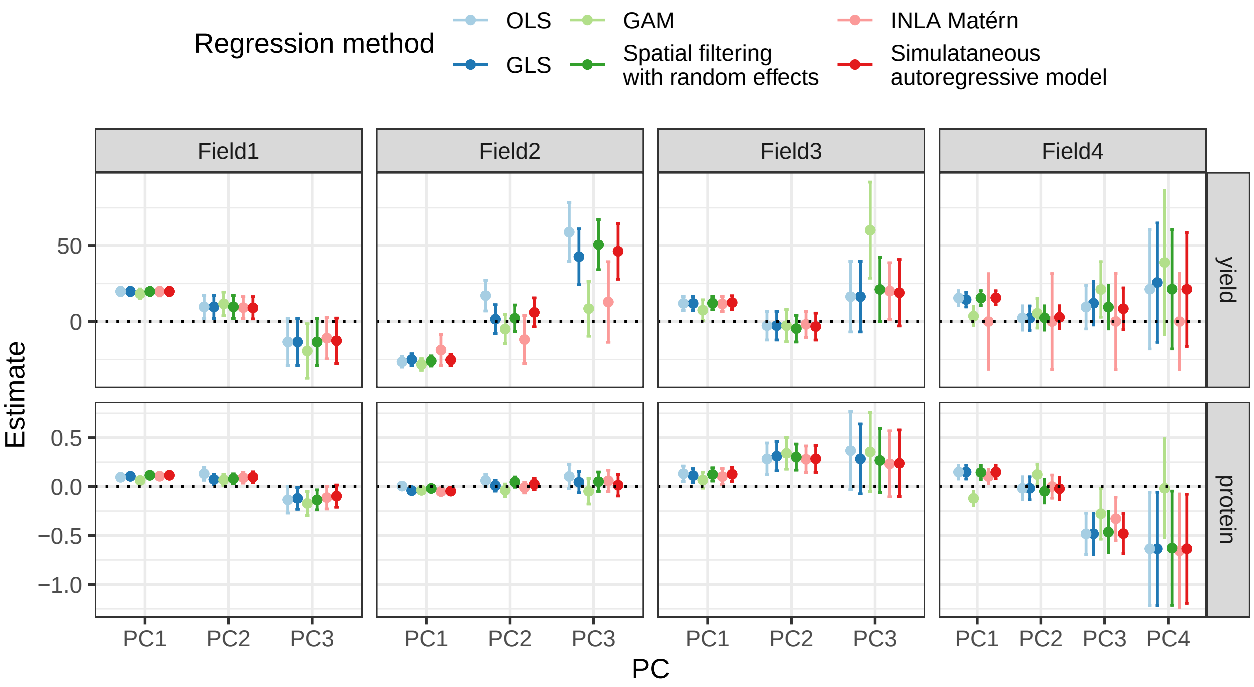

Figure S42: Parameter estimates (y-axis) for different principal components (x-axis) as a function of regression model (colour), training set (columns) and trait (rows). Error bars indicate standard errors (for INLA, the standard deviation of the posterior distribution of the parameters is displayed). As principal components differ between fields, estimates should only be compared among the regression methods within the columns.

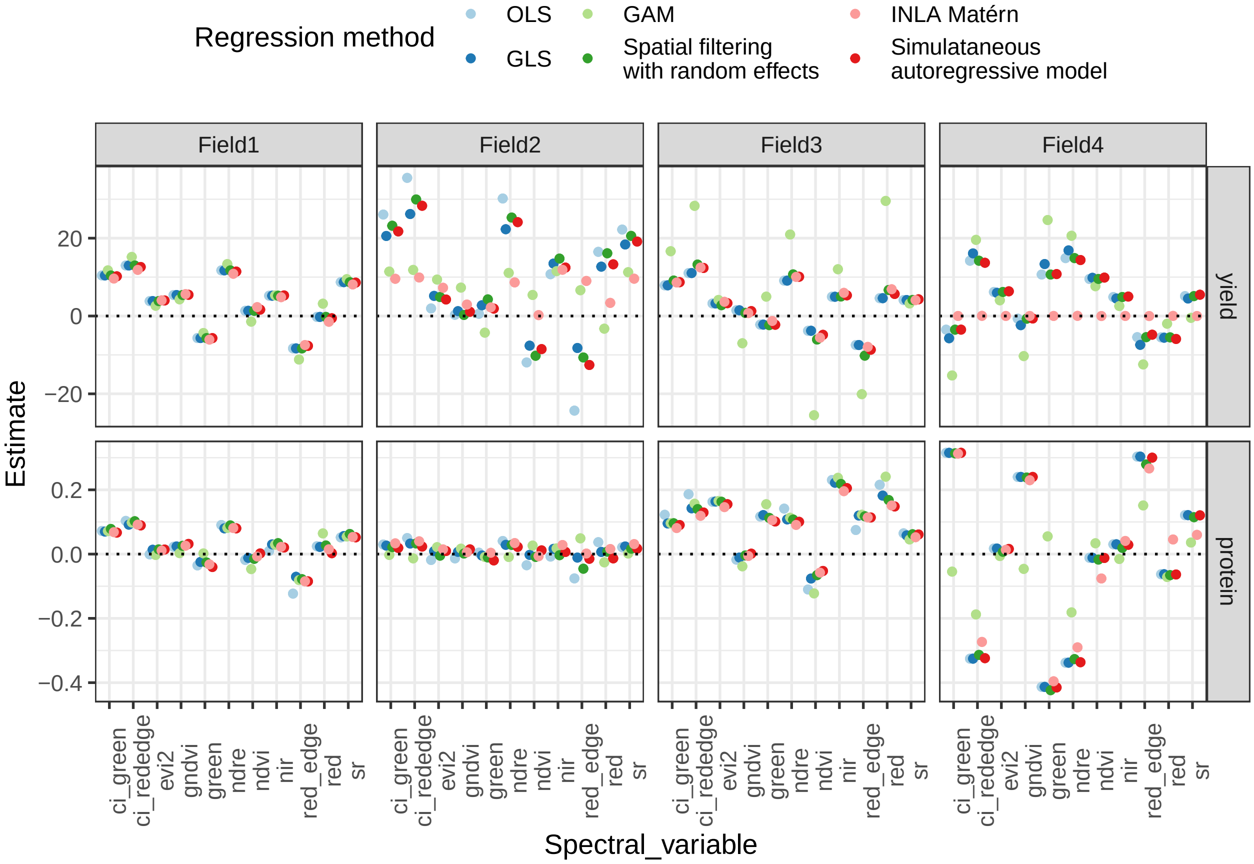

Figure S43: Sums of the products of the parameters with principal component loadings $\boldsymbol{\beta}\mathbf{u1}$, with $\mathbf{u}$ the matrix of principal components included in the model (y-axis) for different spectral variables (x-axis) as a function of regression model (colour), training set (columns) and trait (rows). Parameter loadings can be compared across fields in this case.

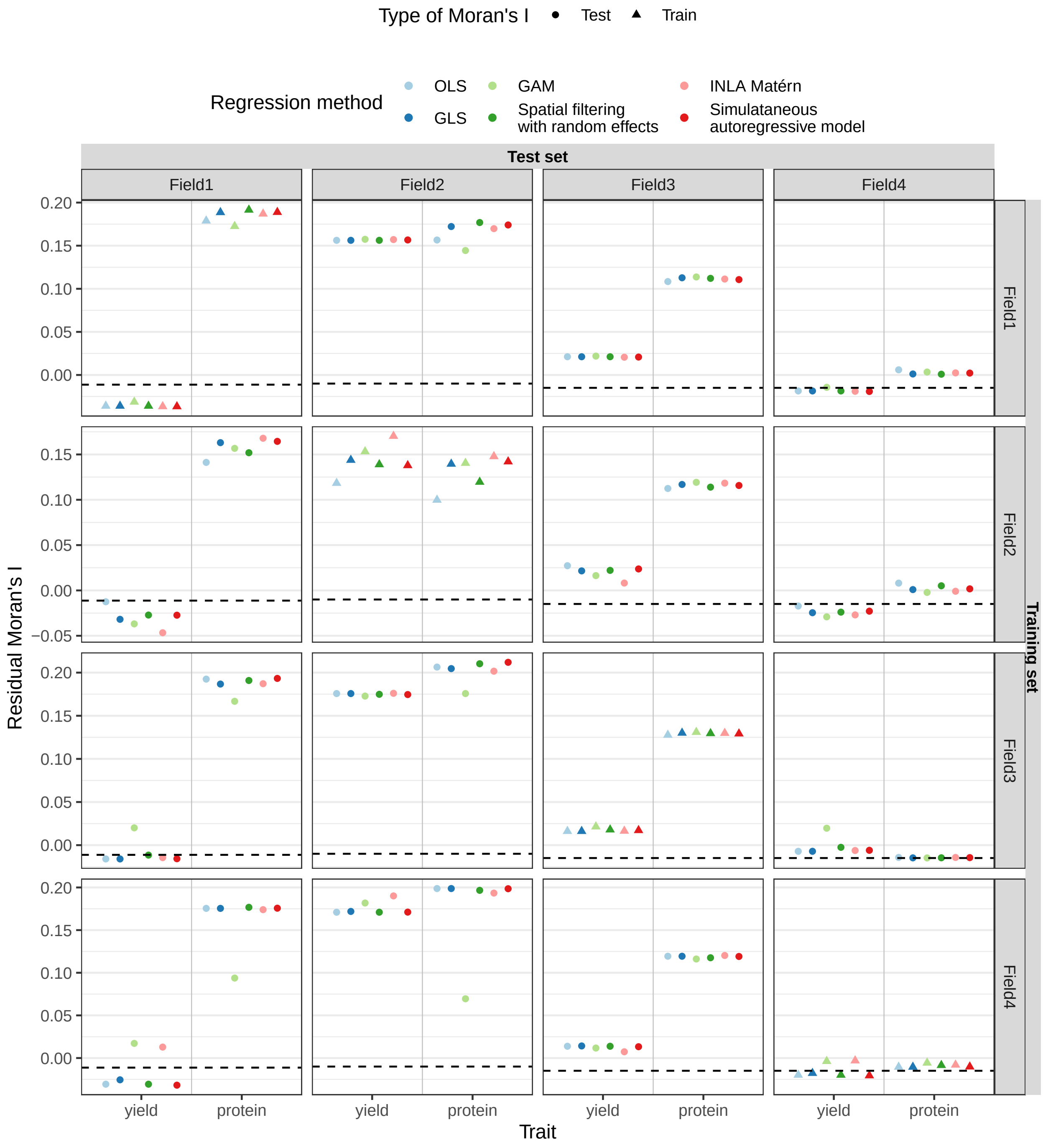

Figure S44: Moran's I statistic of the residuals (y-axis) for models trained on different fields (rows) and applied to all four fields (columns), as a function of outcome variable (x-axis), when applying different methods (colours). Dashed line represents expected value of the statistic in absence of spatial autocorrelation.

3 A closer look at parameter estimation in the GLS method

3.1 Point estimates

The GLS algorithm estimates three parameters related to variance and covariance of the residuals: residual variance (σ^2), nugget (τ) and range (r). The nugget parameter lies between 0 and 1 and reflects the proportion of total variance that is attributed to independent identically distributed noise (e.g. measurement error), as opposed to spatially autocovarying noise [1]. For the case of Gaussian correlation, the range parameter is the distance at which the spatial autocorrelation between two observations equals $\exp(-1)(1-\tau) = 0.368(1-\tau)$. Figure S45 shows their estimates for the univariate scenarios over 100 Monte-Carlo instances with β drawn from a zero-mean normal distribution with standard deviation 0.5. Spatial structure of the outcome is the main driver of the distribution of the estimates. If there is no spatial structure in the outcome, the nugget is large and the residual variance is small. With the nugget close to 1, the range has no effect on the covariance structure and can vary without constraint. For linear effects in the outcome, the nugget is almost 0, the range is much larger than the dimensions of the plot, and the residual variance is large (much larger than the marginal variance of the outcome, see Figure S1). Edge effects in the outcome lead to small nuggets and intermediate residual variances, except when the regressors also experience an edge effect. Gaussian correlation structures lead to correctly estimated nuggets of around 0.25. These results illustrate the flexibility of the three parameter variance-covariance model of GLS to accommodate many different types of spatial patterns.

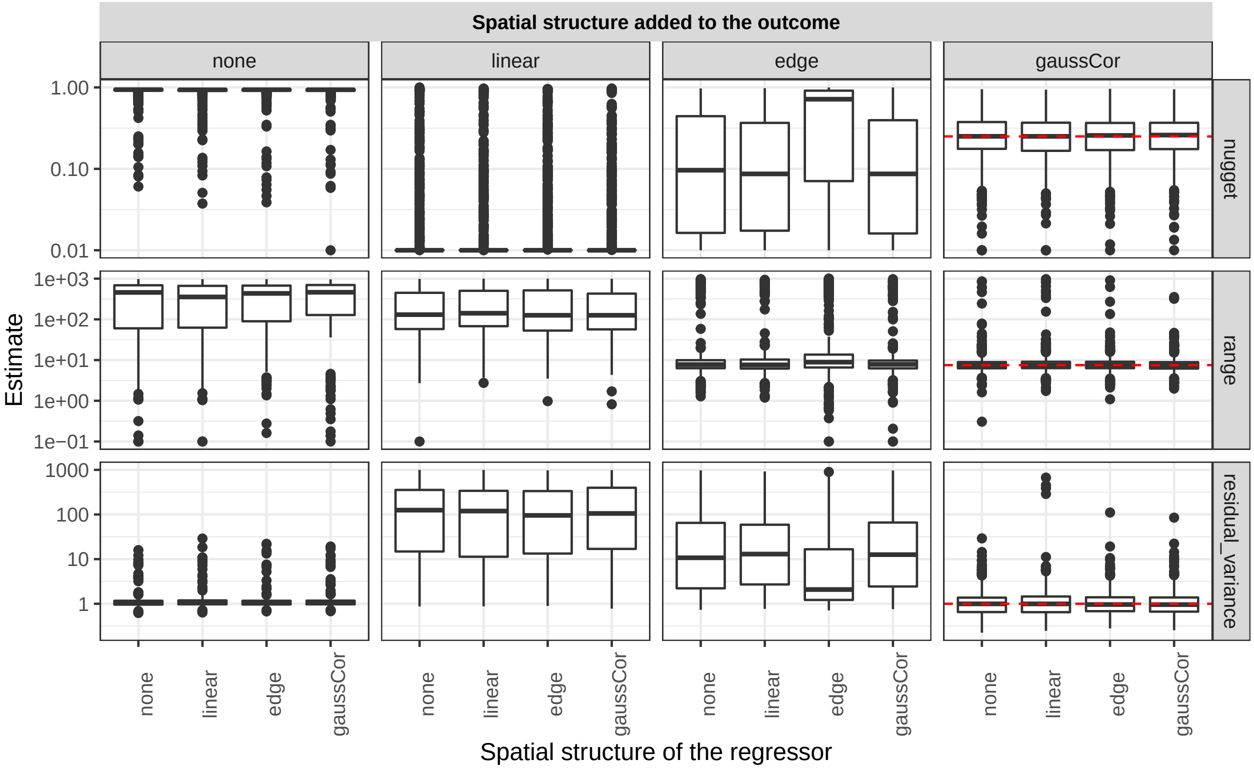

Figure S45: Boxplots of parameter estimates over 1,000 Monte Carlo instances (y-axis on log10-scale) for range, nugget and σ^2 (residual variance) of GLS (rows) for different spatial structures of the regressors (x-axis) and outcome (columns). Nuggets smaller than 0.01 were set to 0.01, and ranges smaller than 0.1 were set to 0.1 for legibility. The red dashed line indicates true parameter values for the Gaussian covariance case. Only ranges and variances below 1,000 are shown.

3.2 Correlations of the parameter estimators

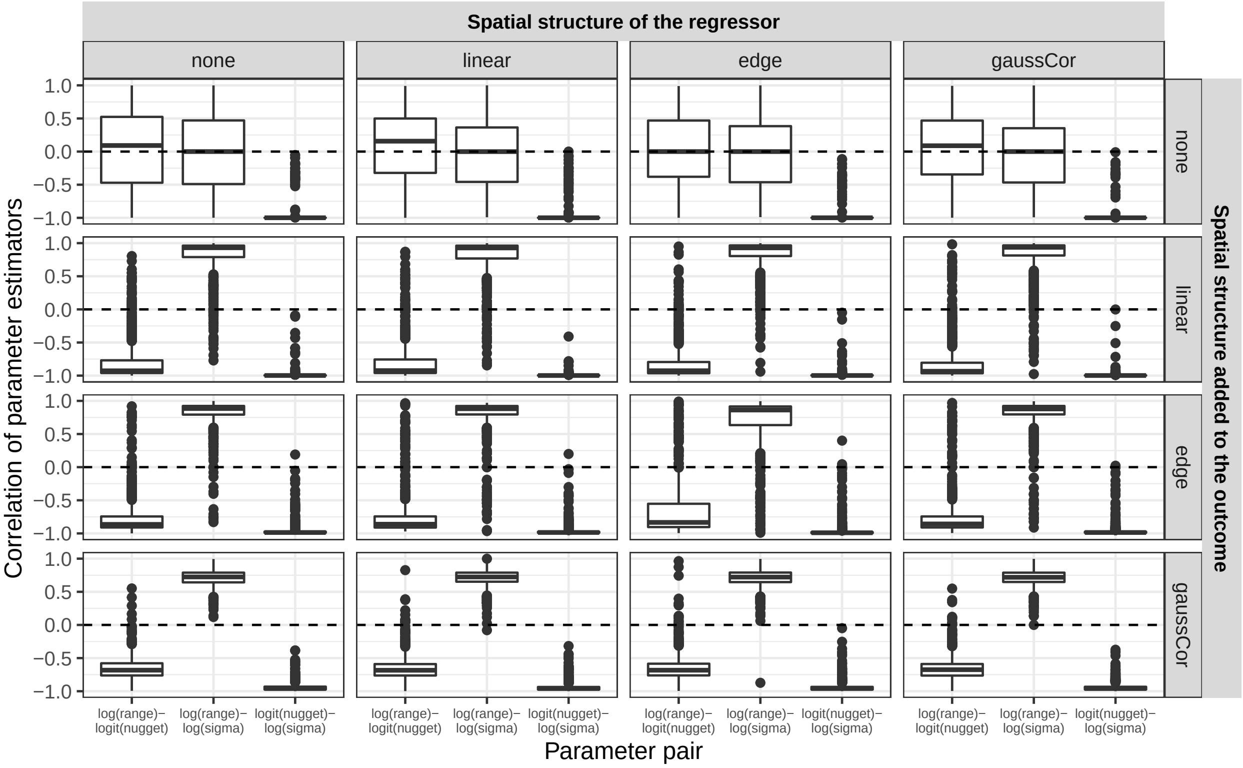

Figure S46: Boxplots of approximate estimated correlations of parameter estimators over 1,000 Monte Carlo instances (y-axis) for combinations of range, nugget and σ^2 (residual variance) of GLS (x-axis) for different spatial structures of the regressors (rows) and outcome (columns).

4 Computation times

Average computation times on a single Intel 2.6 GHz core over 10 repeats of the different methods for the low and high-dimensional scenarios are shown in Table S1. Samples sizes were 100 and 50, and number of features was 50 and 100 for the low- and high-dimensional scenario respectively. No spatial structure was introduced in either outcome or regressors. In all cases, 5 fold blocked cross-validation was applied. The *fmesher* function in of the INLA Matérn model was allowed to run for 1 minute in this case.

Method	Seconds
OLS	0.157
GLS	1.86
GAM	1.38
Spatial filtering random effects	1.54
Simultaneous autoregressive model	3.11
INLA AR(1)	NaN
INLA Matérn	51.9
EN	0.107
GLS EN	27.9
GAM EN	268
Spatial filtering EN	0.129
INLA AR(1) (high-dimensional)	43.9
INLA Matérn (high-dimensional)	47.9

Table S1: Average computation times in seconds of different regression methods over 10 repeats. NaN indicates model fitting failed for all 10 repeats.

5 Software versions

R-software and package versions are shown below. The *plsmselect* package is a custom version that allows the user to specify the mixing parameter α, thus allowing ridge regression and elastic net to be fitted. It is available on the journal's website.

```
## R version 4.0.3 (2020-10-10)
## Platform: x86_64-pc-linux-gnu (64-bit)
## Running under: Ubuntu 20.04.3 LTS
##
## Matrix products: default
## BLAS:   /usr/lib/x86_64-linux-gnu/blas/libblas.so.3.9.0
## LAPACK: /usr/lib/x86_64-linux-gnu/lapack/liblapack.so.3.9.0
##
## locale:
##  [1] LC_CTYPE=en_US.UTF-8       LC_NUMERIC=C
##  [3] LC_TIME=en_GB.UTF-8        LC_COLLATE=en_US.UTF-8
##  [5] LC_MONETARY=en_GB.UTF-8    LC_MESSAGES=en_US.UTF-8
##  [7] LC_PAPER=en_GB.UTF-8       LC_NAME=C
##  [9] LC_ADDRESS=C               LC_TELEPHONE=C
## [11] LC_MEASUREMENT=en_GB.UTF-8 LC_IDENTIFICATION=C
##
## attached base packages:
## [1] grid      parallel  stats     graphics  grDevices utils     datasets
## [8] methods   base
##
## other attached packages:
##  [1] som.nn_1.1.0     gtable_0.3.0     raster_3.5-2     spmoran_0.2.2
```

```
##  [5] mvtnorm_1.1-3    spdep_1.1-12     spatialreg_1.2-1 sf_1.0-4
##  [9] spData_2.0.1     glmnet_4.1-3     xtable_1.8-4     INLA_21.02.23
## [13] sp_1.4-6         foreach_1.5.1    Matrix_1.3-4     plsmselect_0.3.0
## [17] mgcv_1.8-38      nlme_3.1-153     vegan_2.5-7      lattice_0.20-45
## [21] permute_0.9-5    reshape2_1.4.4   ggplot2_3.3.5
##
## loaded via a namespace (and not attached):
##  [1] doParallel_1.0.16  gmodels_2.18.1     RColorBrewer_1.1-2 tools_4.0.3
##  [5] utf8_1.2.2         R6_2.5.1           KernSmooth_2.23-20 DBI_1.1.1
##  [9] colorspace_2.0-2   withr_2.4.2        tidyselect_1.1.1   gridExtra_2.3
## [13] compiler_4.0.3     expm_0.999-6       labeling_0.4.2     scales_1.1.1
## [17] hexbin_1.28.2      classInt_0.4-3     proxy_0.4-26       stringr_1.4.0
## [21] digest_0.6.28      rmarkdown_2.11     pkgconfig_2.0.3    htmltools_0.5.2
## [25] highr_0.9          fastmap_1.1.0      maps_3.4.0         rlang_0.4.12
## [29] FNN_1.1.3          farver_2.1.0       shape_1.4.6        generics_0.1.1
## [33] gtools_3.9.2       dplyr_1.0.7        magrittr_2.0.1     s2_1.0.7
## [37] dotCall64_1.0-1    Rcpp_1.0.7         munsell_0.5.0      fansi_0.5.0
## [41] viridis_0.6.2      lifecycle_1.0.1    terra_1.4-20       stringi_1.7.5
## [45] yaml_2.2.1         MASS_7.3-54        plyr_1.8.6         gdata_2.18.0
## [49] crayon_1.4.2       deldir_1.0-6       splines_4.0.3      knitr_1.36
## [53] pillar_1.6.4       boot_1.3-28        codetools_0.2-18   LearnBayes_2.15.1
## [57] wk_0.5.0           glue_1.5.0         evaluate_0.14      vctrs_0.3.8
## [61] spam_2.7-0         purrr_0.3.4        assertthat_0.2.1   xfun_0.28
## [65] e1071_1.7-9        RSpectra_0.16-0    coda_0.19-4        class_7.3-19
## [69] survival_3.2-13    viridisLite_0.4.0  rARPACK_0.11-0     tibble_3.1.6
## [73] iterators_1.0.13   fields_13.3        units_0.7-2        cluster_2.1.2
## [77] ellipsis_0.3.2
```

References

1. Gringarten, E. & Deutsch, C. V. *Mathematical Geology* **33,** 507 –534 (2001).
2. Zhou, X., Kono, Y., Win, A., Matsui, T. & Tanaka, T. S. T. Predicting within-field variability in grain yield and protein content of winter wheat using UAV-based multispectral imagery and machine learning approaches. *Plant Production Science* **24,** 137 –151 (2021).
