# Supplementary material for: Spatial Regression Models for Field Trials: A Comparative Study and New Ideas
Source: Front Plant Sci. 2022 Mar 30;13:858711. doi: 10.3389/fpls.2022.858711 (PMC9006620; doi:10.3389/fpls.2022.858711)
Supplement: Supplementary file 3 [file Data_Sheet_3.zip › plsmselect/vignettes/plsmselect.html]

The plsmselect package


# The plsmselect package

#### Indrayudh Ghosal and Matthias Kormaksson

#### April 07, 2021

## 1. Introduction

Generalized Additive Models (GAMs) are characterized by a linear predictor that can be broken down into a sum of a linear term (the \(X\beta\) term) and a smooth term (the \(\sum\_j f\_j(x\_j)\) term). The goal of `plsmselect` is to provide a flexible interface for parameter estimation under various penalty structures on the linear parameters \(\beta\) (“none”, \(\ell\_1\), or \(\ell\_2\)) and the *smooth* functions \(f\_j\)’s (\(\ell\_1\) or \(\ell\_2\)). Most noteworthy, the package allows users to fit so called *GAMLASSO* models that add an \(\ell\_1\) penalty on both the linear parameters \(\beta\) and the smooth functions \(f\_j\)’s. Extension to Cox regression is also implemented, which allows users to fit *GAMLASSO* models to censored time-to-event data. Demonstration of package usage for all families (`gaussian`, `binomial`, `poisson`, and `cox`) can be found in the Examples section and users that are more interested in package functionalities (than methodological details) may skip right to that section.

To the best of our knowledge this is the first R-package that can fit GAM models with \(\ell\_1\) penalty on both the linear and smooth terms (i.e. so called *GAMLASSO* models) for `gaussian`, `binomial`, `poisson`, and `cox` families. (Chouldechova and Hastie 2015) proposed a similar method that selects between fitting each of the smooth terms \(f\_j\)’s as zero, linear, or nonlinear, as determined by the data. This method has been implemented in the R-package `gamsel` (Chouldechova, Hastie, and Spinu 2018) but is only applicable for the `gaussian` and `binomial` families. When dealing with continuous variables both `gamsel` and `plsmselect` retain the interpretability advantages of linear (or zero) fits when appropriate, while capturing strong non-linear relationships when they are present.

The main idea of the *GAMLASSO* model involves combining codes from the two benchmark packages for fitting GAM models on the one hand, `mgcv` (Wood 2019), and LASSO models on the other, `glmnet` (Friedman et al. 2019). The former package allows for flexible modeling of non-linear effects (but does not facilitate \(\ell\_1\) penalty on linear terms) while the latter package allows for \(\ell\_1\) penalty on linear terms (but does not facilitate estimation of non-linear effects). `plsmselect` allows for both by borrowing strength between the two. The main model object of `plsmselect` (`gamlasso`) in fact inherits objects from `mgcv::gam` and `glmnet::cv.glmnet` making it particularly easy to work with for users that are familiar with both of those packages.

## 2. Methods

The goal of `plsmselect` is to estimate the parameters of Generalized Additive Models of the form \[\begin{align}
g(\mu) = \beta\_0 + X \beta + \sum\_{j=1}^q f\_j(Z\_j), \qquad\qquad (1)
\end{align}\] under different penalty structures on the linear parameters \(\beta\) (“none”, \(\ell\_1\), or \(\ell\_2\)) and the *smooth* functions \(f\_j\)’s (\(\ell\_1\) or \(\ell\_2\)). In the above \(\mu\) denotes the mean of a response \(Y\) that is assumed to follow a distribution from the exponential dispersion family (with associated link function \(g\)), \(X\) is a model matrix for *linear* predictors (e.g. indicators corresponding to categorical variables) and \(Z\_j\)’s are *smooth* predictors (e.g. all continuous covariates that potentially have a non-linear relationship with the response).

The package also fits generalized additive models for survival data whose models are of the form: \[\begin{equation}
\lambda(t) = \lambda\_0(t) \exp \left( X \beta + \sum\_{j=1}^q f\_j(Z\_j) \right), \qquad\qquad (2)
\end{equation}\] where \(\lambda(t)\) is the hazard function corresponding to a censored time-to-event response \(T\) and \(\lambda\_0(t)\) is the baseline hazard.

**Penalties**: `gamlasso` - the main function of `plsmselect` deals with all the combinations of penalty structures as presented in the following table:

|  | None | \(\ell\_1\) | \(\ell\_2\) |
| --- | --- | --- | --- |
| \(\beta\) | ✓ | ✓ | ✓ |
| \(f\_j\)’s |  | ✓ | ✓ |

For example having no penalty on \(\beta\) and an \(\ell\_2\) penalty on the \(f\_j\)’s is the same fit as can be obtained by simply using `mgcv::gam`. We could also fit a model with \(\ell\_1\) penalty on \(\beta\) and no smooth component at all in which case we could simply use `glmnet::cv.glmnet`.

The main novelty of `plsmselect` is when we impose \(\ell\_1\) penalty on both \(\beta\) and the \(f\_j\)’s. This means the model expects some of the elements of \(\beta\) to be 0 and some of the functions \(f\_j \equiv 0\), i.e., we need to do variable selection for linear and smooth predictors simultaneously. In this case we use the *GAMLASSO* algorithm described in the following section.

Note that the \(\ell\_1\) and \(\ell\_2\) penalties on \(\beta\) are the standard Lasso and Ridge penalties. As for penalties on the \(f\_j\)’s note that by \(\ell\_2\) penalty we imply the standard smoothness penalty used on the functions (detailed in the background section). The \(\ell\_1\)-type penalty used on the functions \(f\_j\) is actually a variant of the smoothness penalty but it forces a function to be 0 in case it is not significant - in this case it acts similar to the Lasso penalty. See `?mgcv::smooth.terms` for more details on this type of penalty.

### The *GAMLASSO* algorithm

When we are fitting a model with \(\ell\_1\) penalty on the linear coefficients \(\beta\) we apply the *GAMLASSO* algorithm:

---

- \(offset = 0\)
- ***loop until convergence*** of the fitted values
- Fit \(Y \sim \beta\_0 + \sum\_{j=1}^q f\_j(Z\_j) + offset\) with either \(\ell\_1\) or \(\ell\_2\) penalty on \(f\_j\)’s.  
  Set \(offset \leftarrow \hat{\beta}\_0 + \sum\_{j=1}^q \hat{f\_j}(Z\_j)\)
- Fit \(Y \sim X \beta + offset\) with \(\ell\_1\) penalty on \(\beta\).  
  Set \(offset \leftarrow X \hat{\beta}\).

---

We can think of this approach as a **block coordinate descent algorithm**. The first block having the intercept \(\beta\_0\) and the linear coefficients of the basis functions corresponding to the the smooth functions \(f\_j\) (more details in the background section). The second block containing the \(\beta\)’s corresponding to the linear predictors. The former block is implemented by invoking the `mgcv` package while the latter is implemented with `glmnet`.

The other combinations with none or \(\ell\_2\) penalties on \(\beta\) already have closed form solutions. They have been implemented in the `mgcv` package and so in these cases `gamlasso` acts as a wrapper to those implementations.

### Background on fitting a generalized additive model (gam)

For a simple model \(Y = f(X) + \epsilon\), given data \((y\_i,x\_i)\_{i=1}^n\) we can estimate \(f\) assuming it to be of the form \[
f(x) = \sum\_{k=1}^K b\_k(x) \beta\_k,
\] for some basis functions \(b\_k\) and corresponding weights \(\beta\_k\).

In practice the basis functions are known. So replacing \(f\) in the first equation gives us a linear model which we can solve for the \(\beta\_k\) values.

Of course in general the choice of basis functions is key. One choice could be polynomials (spline fitting) with prespecified knots. Now polynomials are sufficient for approximating functions at specific points but could become too “wiggly” over a whole domain. Piecewise linear basis functions gives a “better” fit but is not “smooth”. Cubic regression splines with fixed knots \(x^\*\_1, \dots, x^\*\_K\) provide an attractive solution as a smooth alternative to the intuitive picewise linear splines. With such a spline basis representation we can add a “wigglyness” penalty \[
\lambda \sum\_{k=2}^{K-1} \big(f(x^\*\_{j-1}) - 2f(x^\*\_j) + f(x^\*\_{j+1})\big)^2 = \lambda \beta^T S \beta,
\] The penalty is a second order one, as an approximation to the penalty on second derivatives used in spline fitting. Cross-validation is used for estimating \(\lambda\).

In the standard software used to fit a gam (`mgcv`) the *thin plate regularized spline* (tprs) basis functions are used. More details can be found in (Wood 2003)

For two or more smooth predictors the model is \[
Y = f\_1(X\_1) + f\_2(X\_2) + \dots + \epsilon
\] Now both \(f\_1\) and \(f\_2\) cannot be uniquely identified. So we always have an intercept \(\beta\_0\) and identifiability contraints \[
\sum\_{i=1}^n f\_1(X\_{1i}) = \sum\_{i=1}^n f\_2(X\_{2i}) = 0.
\]

**Note**:

- Both \(f\_1\) and \(f\_2\) will have their own \(\ell\_2\) smoothing/“wigglyness” penalties.
- This model is more restrictive than the general bivariate smoothing model \(Y = f(X\_1, X\_2) + \epsilon\).

In general for fitting a model with linear predictors and/or more than one smooth predictors we can write it as a linear model (similar to the univariate smooth case above) and proceed accordingly.

## 3. Examples

In this section we will go over some of the functionalities of `plsmselect` on a simulated dataset. In particular, we will demonstrate how to use the package to fit Generalized Additive Models under different penalty structures to data arising from the following families: `gaussian`, `binomial`, `poisson`, and `cox`. We will focus our attention on the so called *GAMLASSO* model that involves an \(\ell\_1\) penalty on both linear parameters \(\beta\) and smooth functions \(f\_j\)’s.

In the next subsection we will describe the simulated example data set. After that we demonstrate how to fit the *GAMLASSO* model to `gaussian`, `binomial` and `poisson` responses. Finally, we will show how to fit the Cox *GAMLASSO* model to survival data (censored time-to-event responses).

### Simulated dataset

`plsmselect` comes with a simulated toy data set that we will use to demonstrate package functionalities. This data set `simData` will be used in all below analyses.

```
library(plsmselect)
library(purrr)

data(simData)
```

Table 1. First 6 samples of the simulated data set: simData.

| id | x1 | x2 | x3 | x4 | x5 | x6 | x7 | x8 | x9 | x10 | z1 | z2 | z3 | z4 | lp | Yg | Yb | Yp | success | failure | time | status |
| --- | --- | --- | --- | --- | --- | --- | --- | --- | --- | --- | --- | --- | --- | --- | --- | --- | --- | --- | --- | --- | --- | --- |
| i1 | 0 | 1 | 0 | 0 | 0 | 1 | 1 | 0 | 1 | 0 | 0.27 | 0.80 | 0.86 | 0.03 | 0.01 | -0.12 | 0 | 1 | 3 | 2 | 44.83 | 1 |
| i2 | 1 | 0 | 1 | 1 | 1 | 1 | 1 | 1 | 1 | 1 | 0.59 | 0.73 | 0.89 | 0.49 | 2.46 | 2.54 | 1 | 13 | 2 | 0 | 2.24 | 1 |
| i3 | 0 | 0 | 1 | 0 | 0 | 1 | 1 | 1 | 1 | 0 | 0.16 | 0.22 | 0.49 | 0.54 | 1.13 | 1.05 | 1 | 3 | 1 | 1 | 10.61 | 1 |
| i4 | 1 | 1 | 1 | 0 | 1 | 1 | 1 | 0 | 1 | 1 | 0.85 | 0.74 | 0.72 | 0.84 | 2.24 | 2.17 | 1 | 8 | 9 | 1 | 3.02 | 1 |
| i5 | 1 | 0 | 0 | 1 | 1 | 1 | 1 | 1 | 1 | 0 | 0.85 | 0.87 | 0.49 | 0.73 | 2.02 | 2.03 | 1 | 7 | 3 | 1 | 30.37 | 1 |
| i6 | 0 | 1 | 1 | 1 | 1 | 1 | 0 | 1 | 0 | 1 | 0.48 | 0.53 | 0.99 | 0.56 | 1.00 | 0.97 | 1 | 0 | 2 | 0 | 20.87 | 1 |

- **id:** Sample (subject) ID
- **x1,…,x10:** Binary covariates
- **z1,…,z4:** Continuous covariates
- **lp:** True linear predictor of eq. (1) and (2) in Methods: \(l\_p=x\_1-0.6x\_2+0.5x\_3+z\_1^3 + \sin(\pi \cdot z\_2)\).
- **Yg:** Simulated Gaussian variable with mean \(l\_p\) and standard deviation \(\sigma=0.1\).
- **Yb:** Simulated Bernoulli variable with success probability \(p=\exp(l\_p)/(1+\exp(l\_p))\).
- **success:** Simulated \(Binomial(n,p)\) variable with varying sample size \(n\) per **id**.
- **failure:** \(n-\)**success**.
- **Yp**: Simulated \(Poisson(\lambda)\) variable with mean \(\lambda=\exp(l\_p)\).
- **time**: Time-to-event simulated from Cox-model (2) in Methods with linear predictor \(l\_p\).
- **status:** Indicator for whether the subject experienced event (status\(=1\)) or was censored (status\(=0\)).

The details of how this data is simulated can be found in the Appendix.

### Gaussian GAMLASSO model with `plsmselect`

In this section we fit the following GAM model to the response \(Y\_g\) from `simData` in Table 1: \[\begin{align}
Y\_g = \beta\_0 + X \beta + \sum\_{j=1}^4 f\_j(z\_j) + \varepsilon,
\end{align}\] where both \(\beta\) and the \(f\_j\)’s are subject to \(\ell\_1\) penalties. In the above \(\varepsilon \sim N(0,\sigma^2)\), \(X\) denotes the model matrix corresponding to the binary variables \(x\_1,\dots,x\_{10}\) and \(z\_1, \dots, z\_4\) denote the continuous variables of `simData`.

(Note that the notation here is vectorized, for example the \(i\) element of the vector \(Z\_j\) is used for the \(i\) element of the vector \(Y\_g\). We will use a similar vectorised notation henceforth)

Below we demonstrate the code for fitting this GAMLASSO model using the `plsmselect` formula specification. Note that for the formula specification the model (design) matrix \(X\) needs to be computed and appended to the simData set. The same code generalizes to situations where \(x\_1,\dots,x\_{10}\) are categorical factor variables.

```
## Create model matrix X corresponding to linear terms
## (necessary for the formula option of gamlasso below)
simData$X = model.matrix(~x1+x2+x3+x4+x5+x6+x7+x8+x9+x10, data=simData)[,-1]

## The formula approach
gfit = gamlasso(Yg ~ X +
                  s(z1, k=5, bs="ts") +
                  s(z2, k=5, bs="ts") +
                  s(z3, k=5, bs="ts") +
                  s(z4, k=5, bs="ts"),
                data = simData,
                seed = 1)
```

Alternatively, we can fit the above model using the `plsmselect` term specification:

```
## The term specification approach
gfit = gamlasso(response = "Yg",
                linear.terms = paste0("x",1:10),
                smooth.terms = paste0("z",1:4),
                data = simData,
                linear.penalty = "l1",
                smooth.penalty = "l1",
                num.knots = 5,
                seed = 1)
```

The term specification approach is helpful when you have a large data set with multiple columns, but has the disadvantage that the number of knots is constant across all smooth terms. With the formula approach on the other hand the user has full flexibility in defining different numbers of knots per smooth term.

**`gamlasso` object:** The `gamlasso` object `gfit` is a list with first component corresponding to the smooth gam part of the fit (`gfit$gam`: a `gam` object from the package `mgcv`; see `?mgcv::gam`) and a second component corresponding to the linear lasso part of the fit (`gfit$cv.glmnet`: a `cv.glmnet` object from the package `glmnet`; see `?glmnet::cv.glmnet`).

```
# mgcv::gam object:
class(gfit$gam)
```

```
#> [1] "gam" "glm" "lm"
```

```
# glmnet::cv.glmnet object
class(gfit$cv.glmnet)
```

```
#> [1] "cv.glmnet"
```

The summary of the *GAMLASSO* model can be obtained with the following command:

```
summary(gfit)
```

```
#> $lasso
#> 11 x 1 sparse Matrix of class "dgCMatrix"
#>                       1
#> (Intercept)  .         
#> Xx1          0.99087556
#> Xx2         -0.58532317
#> Xx3          0.54394830
#> Xx4          0.03323999
#> Xx5          .         
#> Xx6          .         
#> Xx7          .         
#> Xx8          .         
#> Xx9          .         
#> Xx10         0.02623600
#> 
#> $gam
#> 
#> Family: gaussian 
#> Link function: identity 
#> 
#> Formula:
#> Yg ~ s(z1, k = 5, bs = "ts") + s(z2, k = 5, bs = "ts") + s(z3, 
#>     k = 5, bs = "ts") + s(z4, k = 5, bs = "ts")
#> 
#> Parametric coefficients:
#>             Estimate Std. Error t value Pr(>|t|)    
#> (Intercept) 0.798303   0.009222   86.57   <2e-16 ***
#> ---
#> Signif. codes:  0 '***' 0.001 '**' 0.01 '*' 0.05 '.' 0.1 ' ' 1
#> 
#> Approximate significance of smooth terms:
#>             edf Ref.df     F p-value    
#> s(z1) 3.573e+00      4 161.7  <2e-16 ***
#> s(z2) 3.518e+00      4 218.6  <2e-16 ***
#> s(z3) 5.519e-10      4   0.0   0.914    
#> s(z4) 1.792e-08      4   0.0   0.543    
#> ---
#> Signif. codes:  0 '***' 0.001 '**' 0.01 '*' 0.05 '.' 0.1 ' ' 1
#> 
#> R-sq.(adj) =  0.987   Deviance explained = 93.8%
#> GCV = 0.009253  Scale est. = 0.0085044  n = 100
```

We note that the summary is broken into a *lasso* summary and a *gam* summary. The *lasso* summary shows the linear parameter estimates and we note that in the above case only the first three linear parameters are estimated as non-zero. Compare these values to the underlying “truth” as defined in the simulation section of Appendix (\(\beta\_1=1\), \(\beta\_2=-0.6\), \(\beta\_3=0.5\), \(\beta\_4=\cdots=\beta\_{10}=0\)). The *gam* summary corresponds to the (`mgcv`) summary of the gam object gfit$gam (see `?mgcv::summary.gam`).

We can visualize the estimates of the smooth functions by calling the `mgcv::plot.gam` function:

```
## Plot the estimates of the smooth effects:
plot(gfit$gam, pages=1)
```

We note that the functions corresponding to the variables \(z\_3\) and \(z\_4\) are estimated to be near zero (confidence bands include zero). Compare these estimates to the underlying “truth” as defined in the simulation section of Appendix (\(f\_1(z)=z^3\), \(f\_2(z)=\sin(\pi\cdot z)\), \(f\_3(z)=f\_4(z)=0\)). Note that `mgcv` applies a sum constraint on each function \(\sum\_k f\_1(z\_{1k})=\cdots=\sum\_k f\_4(z\_{4k})=0\), where the sum is taken across all observations (samples) of \(z\_1,\dots,z\_4\) (see Methods section). Hence the model only estimates the functions (and overall intercept) correctly up to a vertical shift. However, we can verify that the model’s fitted values \(\hat{Y}\_g\) match nicely those that were observed \(Y\_g\):

```
## Plot fitted versus observed values:
plot(simData$Yg, predict(gfit), xlab = "Observed values", ylab = "Fitted Values")
```

### Poisson GAMLASSO regression with `plsmselect`

In this section we fit a Poisson regression model to the response \(Y\_p \sim Poi(\lambda)\) from `simData` \[\begin{align}
\log(\lambda) = \beta\_0 + X \beta + \sum\_{j=1}^4 f\_j(z\_j),
\end{align}\] where both \(\beta\) and the \(f\_j\)’s are subject to \(\ell\_1\) penalties.

The above model can be fit using the formula approach:

```
## Create model matrix X corresponding to linear terms
## (necessary for the formula option of gamlasso below)
simData$X = model.matrix(~x1+x2+x3+x4+x5+x6+x7+x8+x9+x10, data=simData)[,-1]

## Poisson response. Formula approach.
pfit = gamlasso(Yp ~ X + 
                  s(z1, bs="ts", k=5) + 
                  s(z2, bs="ts", k=5) + 
                  s(z3, bs="ts", k=5) + 
                  s(z4, bs="ts", k=5),
                data = simData,
                family = "poisson",
                seed = 1)
```

or alternatively with the term specification approach:

```
## Poisson response. Term-specification approach.
pfit = gamlasso(response = "Yp",
                linear.terms = paste0("x",1:10),
                smooth.terms = paste0("z",1:4),
                data = simData,
                linear.penalty = "l1",
                smooth.penalty = "l1",
                family = "poisson",
                num.knots = 5,
                seed = 1)
```

We can obtain the linear parameter estimates directly:

```
coef(pfit$cv.glmnet, s="lambda.min")
```

```
#> 11 x 1 sparse Matrix of class "dgCMatrix"
#>                      1
#> (Intercept)  .        
#> Xx1          0.7535747
#> Xx2         -0.4572487
#> Xx3          0.4357671
#> Xx4          .        
#> Xx5          .        
#> Xx6          .        
#> Xx7          .        
#> Xx8          .        
#> Xx9          .        
#> Xx10         .
```

and plot the smooth estimates of individual terms:

```
par(mfrow=c(1,2))
plot(pfit$gam, select=1) # estimate of smooth term z1
plot(pfit$gam, select=2) # estimate of smooth term z2
```

We note that the linear parameter estimates reasonably capture the underlying truth: \(\beta\_1=1\), \(\beta\_2=-0.6\), \(\beta\_3=0.5\), \(\beta\_4=\dots,\beta\_{10}=0\) (only shrunk towards zero as expected). Similarly we see that the estimates of the smooth effects corresponding to \(z\_1\) and \(z\_2\) are consistent with the true functions \(f\_1(z)=z^3\) and \(f\_2(z)=\sin(\pi\cdot z)\) up to a constant vertical shift (due to sum constraint). We can further verify how well the model estimates the true expected counts \(\exp(l\_p)\):

```
plot(predict(pfit, type="response"), exp(simData$lp), xlab="predicted count", ylab="true expected count")
```

### Binary logistic GAMLASSO regression with `plsmselect`

In this section we fit a binary logistic regression model to the response \(Y\_b \sim Bernoulli(p)\) from `simData`: \[\begin{align}
\log\left(\frac{p}{1-p}\right) = \beta\_0 + X \beta + \sum\_{j=1}^4 f\_j(z\_j),
\end{align}\] where both \(\beta\) and the \(f\_j\)’s are subject to \(\ell\_1\) penalties.

The above model can be fit using the formula approach:

```
## Create model matrix X corresponding to linear terms
## (necessary for the formula option of gamlasso below)
simData$X = model.matrix(~x1+x2+x3+x4+x5+x6+x7+x8+x9+x10, data=simData)[,-1]

## Bernoulli trials response
bfit = gamlasso(Yb ~ X + 
                  s(z1, bs="ts", k=5) + 
                  s(z2, bs="ts", k=5) + 
                  s(z3, bs="ts", k=5) + 
                  s(z4, bs="ts", k=5),
                data = simData,
                family = "binomial",
                seed = 1)
```

or alternatively using the term specification approach:

```
## The term specification approach
bfit = gamlasso(response = "Yb",
                linear.terms = paste0("x",1:10),
                smooth.terms = paste0("z",1:4),
                data = simData,
                family="binomial",
                linear.penalty = "l1",
                smooth.penalty = "l1",
                num.knots = 5,
                seed = 1)
```

We can evalute the `summary`, plot the smooth terms and compare predicted probabilities to underlying truth as above (not evaluated):

```
summary(bfit)
plot(bfit$gam, pages=1)
pred.prob <- predict(bfit, type="response")
true.prob <- exp(simData$lp)/(1+exp(simData$lp))
plot(pred.prob, true.prob, xlab="predicted probability", ylab="true probability")
```

Note that the above model does not fit the data well since this is a small data set (\(N=100\)) for Binary logistic regression.

### Binomial counts GAMLASSO model with `plsmselect`

In this section we fit a binomial logistic regression model to the response \(success \sim Binomial(n, p)\) from `simData` (where \(n\) denotes varying count totals across subjects **id**): \[\begin{align}
\log\left(\frac{p}{1-p}\right) = \beta\_0 + X \beta + \sum\_{j=1}^4 f\_j(z\_j),
\end{align}\] where both \(\beta\) and the \(f\_j\)’s are subject to \(\ell\_1\) penalties.

We define \(failure=n-success\) and use the formula specification approach to fit the above model:

```
## Create model matrix X corresponding to linear terms
## (necessary for the formula option of gamlasso below)
simData$X = model.matrix(~x1+x2+x3+x4+x5+x6+x7+x8+x9+x10, data=simData)[,-1]

## Binomial counts response. Formula approach.
bfit2 = gamlasso(cbind(success,failure) ~ X + 
                   s(z1, bs="ts", k=5) + 
                   s(z2, bs="ts", k=5) + 
                   s(z3, bs="ts", k=5) + 
                   s(z4, bs="ts", k=5),
                 data = simData,
                 family = "binomial",
                 seed = 1)
```

or alternatively using the term specification approach:

```
## Binomial counts response. Term specification approach
bfit2 = gamlasso(c("success","failure"),
                 linear.terms=paste0("x",1:10),
                 smooth.terms=paste0("z",1:4),
                 data=simData,
                 family = "binomial",
                 linear.penalty = "l1",
                 smooth.penalty = "l1",
                 num.knots = 5,
                 seed=1)
```

As in the Bernoulli Example we can evalute the `summary`, plot the smooth terms and compare predicted probabilities to underlying truth (not evaluated):

```
summary(bfit2)
plot(bfit2$gam, pages=1)
pred.prob <- predict(bfit2, type="response")
true.prob <- exp(simData$lp)/(1+exp(simData$lp))
plot(pred.prob, true.prob, xlab="predicted probability", ylab="true probability")
```

### Cox GAMLASSO with `plsmselect`

In this section we fit a Cox regression model to the censored event `time` variable from `simData` (`status`=1 if event is observed, 0 if censored): \[\begin{align}
\lambda(t) = \lambda\_0(t) \exp \left( \beta\_0 + X \beta + \sum\_{j=1}^4 f\_j(z\_j) \right),
\end{align}\] where both \(\beta\) and the \(f\_j\)’s are subject to \(\ell\_1\) penalties.

We fit the above Cox GAMLASSO model using the formula approach:

```
## Create model matrix X corresponding to linear terms
## (necessary for the formula option of gamlasso below)
simData$X = model.matrix(~x1+x2+x3+x4+x5+x6+x7+x8+x9+x10, data=simData)[,-1]

# Censored time-to-event response. Formula approach.
cfit = gamlasso(time ~ X +
                  s(z1, bs="ts", k=5) +
                  s(z2, bs="ts", k=5) +
                  s(z3, bs="ts", k=5) +
                  s(z4, bs="ts", k=5),
                data = simData,
                family = "cox",
                weights = "status",
                seed = 1)
```

or alternatively using the term specification approach:

```
# Censored time-to-event response. Term specification approach.
cfit = gamlasso(response = "time",
                linear.terms = paste0("x",1:10),
                smooth.terms = paste0("z",1:4),
                data = simData,
                linear.penalty = "l1",
                smooth.penalty = "l1",
                family = "cox",
                weights="status",
                num.knots = 5,
                seed = 1)
```

Once the model is fit we can perform similar diagnostics as in the Gaussian, Poisson, Bernoulli and Binomial examples above. But in addition since this is a survival model we can also estimate the hazard function. In particular the function `cumbasehaz` will output the estimated cumulative baseline hazard function.

```
## Obtain and plot predicted cumulative baseline hazard:
H0.pred <- cumbasehaz(cfit)

time.seq <- seq(0, 60, by=1)
plot(time.seq, H0.pred(time.seq), type="l", xlab = "Time", ylab="",
     main = "Predicted Cumulative \nBaseline Hazard")
```

We can predict the survival probability \(S(t)=P(T>t)\) for each sample at a single or a sequence of event times \(t\).

```
## Obtain predicted survival at days 1,2,3,...,60:
S.pred <- predict(cfit, type="response", new.event.times=1:60)

## Plot the survival curve for sample (subject) 17:
plot(1:60, S.pred[17,], xlab="time (in days)", ylab="Survival probability", type="l")
```

Note that the object `S.pred` above is a matrix whose rows are samples (subjects) and columns are the new event times (at which survival probabilities are calculated).

## A. Appendix

### Data simulation (details)

We provide below the code that was used to generate `simData`. Note that due to simulation using different seeds the output from the code (`simData2`) below may not exactly match `data(simData)`.

**Simulate covariates:**

We first simulate the covariate predictors with the helper function `generate.inputdata` below. We simulate \(N=100\) samples of \(10\) binary (linear) predictors \(x\_1,\dots,x\_{10}\) and \(4\) continuous (smooth) predictors \(z\_1,\dots,z\_4\).

```
generate.inputdata <- function(N) {
  
  id <- paste0("i",1:N)
  ## Define linear predictors
  linear.pred <- matrix(c(rbinom(N*3,size=1,prob=0.2),
                          rbinom(N*5,size=1,prob=0.5),
                          rbinom(N*2,size=1,prob=0.9)),
                        nrow=N)
  colnames(linear.pred) = paste0("x", 1:ncol(linear.pred))
  
  ## Define smooth predictors
  smooth.pred = as.data.frame(matrix(runif(N*4),nrow=N))
  colnames(smooth.pred) = paste0("z", 1:ncol(smooth.pred))
  
  ## Coalesce the predictors
  dat = cbind.data.frame(id, linear.pred, smooth.pred)
  
  return(dat)
  
}

## Simulate N input data observations:
N <- 100
simData2 <- generate.inputdata(N)
```

We encourage the user to change `N` to a higher number (e.g. \(N=1000\)) and rerun the code in the Examples section with the new `simData2` in order to get a better model fit.

**Calculate “true” linear predictor:**

In all below simulations (of the various response types `gaussian`, `binomial`, `poisson`, and `cox`) we will assume that the “true” underlying linear predictor is \[
l\_p = x\_1-0.6x\_2+0.5x\_3+z\_1^3 + \sin(\pi \cdot z\_2),
\] i.e. we assume that the linear parameters associated with \(x\_1,\dots,x\_{10}\) are respectively \(\beta\_1=1\), \(\beta\_2=-0.6\), \(\beta\_3=0.5\), \(\beta\_4=\cdots=\beta\_{10}=0\) and the functional relationships between \(l\_p\) and \(z\_1,\dots,z\_4\) are respectively \(f\_1(z)=z^3\), \(f\_2(z)=\sin(\pi\cdot z)\), \(f\_3(z)=f\_4(z)=0\). The below R code calculates the “true” linear predictor based on the above formula.

```
## "True" coefficients of linear terms (last 7 are zero):
beta <- c(1, -0.6, 0.5, rep(0,7))

## "True" nonlinear smooth terms:
f1 <- function(x) x^3
f2 <- function(x) sin(x*pi)

## Calculate "True" linear predictor (lp) based on above data (simData2)
simData2$lp <- as.numeric(as.matrix(simData2[,paste0("x",1:10)]) %*% beta + f1(simData2$z1) + f2(simData2$z2))
```

**Simulate Gaussian, Bernoulli, and Poisson responses:**

We then go on and simulate `gaussian`, `bernoulli`, and `poisson` responses assuming the above linear predictor as ground truth: \[\begin{align}
Y\_g &\sim N(\mu,\sigma^2), &&\mu=l\_p, \sigma=0.1, \\
Y\_b &\sim Bernoulli(p), &&\log\left(\frac{p}{1-p}\right)=l\_p, \\
Y\_p &\sim Poi(\lambda), &&\log(\lambda) = l\_p,
\end{align}\]

```
## Simulate gaussian response with mean lp:
simData2$Yg = simData2$lp + rnorm(N, sd = 0.1)

## Simulate bernoulli trials with success probability exp(lp)/(1+exp(lp))
simData2$Yb = map_int(exp(simData2$lp), ~ rbinom(1, 1, p = ( ./(1+.) ) ) )

## Simulate Poisson response with log(mean) = lp
simData2$Yp = map_int(exp(simData2$lp), ~rpois(1, .) )
```

**Simulate Binomial counts:**

We then simulate binomial success/failure counts (with varying count totals \(n\) also simulated) assuming the above linear predictor as ground truth: \[\begin{align}
n &\sim unif\{1,\dots,10\}, \\
success &\sim Binomial(n, p), \\
failure &= n - success,
\end{align}\] where \(p = \exp(l\_p)/(1+\exp(l\_p))\).

```
## Simulate binomial counts with success probability exp(lp)/(1+exp(lp))
sizes = sample(10, nrow(simData2), replace = TRUE)
# Simulate success:
simData2$success = map2_int(exp(simData2$lp), sizes, ~rbinom(1, .y, p = ( .x/(1+.x) )))
# Calculate failure:
simData2$failure = sizes - simData2$success
```

**Simulate time-to-event response from Weibull model:**

Finally we simulate censored event times \(T\) according to a Cox regression model with exponential baseline hazard (i.e. baseline hazard function is constant), assuming the above linear predictor as ground truth. The censoring times \(C\) are also simulated according to an exponential distribution. \[\begin{align}
T &\sim Exp(\lambda(t)), \textrm{ } \lambda(t)=\lambda\_0 e^{l\_p}, \nonumber \\
C &\sim Exp(\lambda\_C)
\end{align}\] The censoring variable is then defined as \(status=1(T \geq C)\).

```
## Function that simulates N samples of censored event times (time, status)
## Event times ~ Weibull(lambda0, rho) with linear predictor lp
## rho=1 corresponds to exponential distribution
simulWeib <- function(N, lambda0, rho, lp)
{
  
  # Censoring times ~ Exponential(lambdaC)
  lambdaC=0.0005 # very mild censoring
  
  # Simulate Weibull latent event times
  v <- runif(n=N)
  Tlat <- (- log(v) / (lambda0 * exp(lp)))^(1 / rho)
  
  # Simulate censoring times
  C <- rexp(n=N, rate=lambdaC)
  
  # Calculate follow-up times and event indicators
  time <- pmin(Tlat, C)
  status <- as.numeric(Tlat <= C)
  
  data.frame(time=time, status=status)
  
}

## Set parameters of Weibull baseline hazard h0(t) = lambda*rho*t^(rho-1):
lambda0 <- 0.01; rho <- 1;

## Simulate (times, status) from above censored Weibull model and cbind to our data:
simData2 <- cbind.data.frame(simData2, simulWeib(N, lambda0, rho, simData2$lp))
```

# References

Chouldechova, Alexandra, and Trevor Hastie. 2015. “Generalized Additive Model Selection.” *arXiv:1506.03850*.

Chouldechova, Alexandra, Trevor Hastie, and Vitalie Spinu. 2018. *Gamsel: Fit Regularization Path for Generalized Additive Models*.

Friedman, J., T. Hastie, R. Tibshirani, N. Simon, B. Narasimhan, and J. Qian. 2019. *Glmnet: Lasso and Elastic-Net Regularized Generalized Linear Models*.

Wood, Simon. 2019. *Mgcv: Mixed Gam Computation Vehicle with Automatic Smoothness Estimation*.

Wood, Simon N. 2003. “Thin Plate Regression Splines.” *Journal of the Royal Statistical Society: Series B (Statistical Methodology)* 65 (1): 95–114.
