# Supplementary figures and images for: Spatial Regression Models for Field Trials: A Comparative Study and New Ideas
Source: Front Plant Sci. 2022 Mar 30;13:858711. doi: 10.3389/fpls.2022.858711 (PMC9006620; doi:10.3389/fpls.2022.858711)

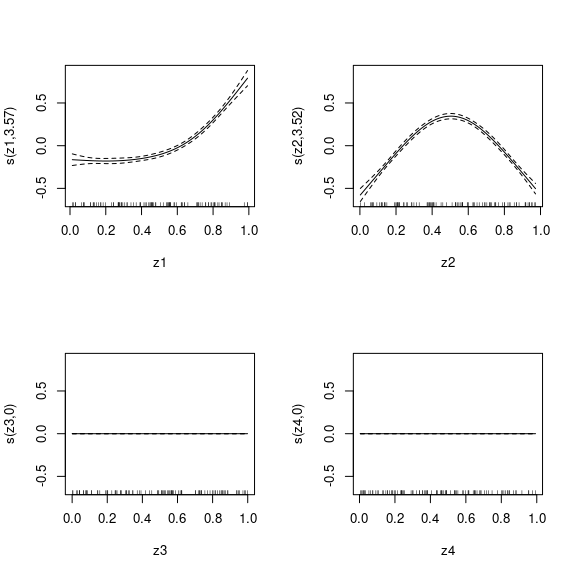

Supplement: Supplementary file 3 [file Data_Sheet_3.zip › plsmselect/vignettes/plsmselect_files/figure-html/plotgam-1.png]

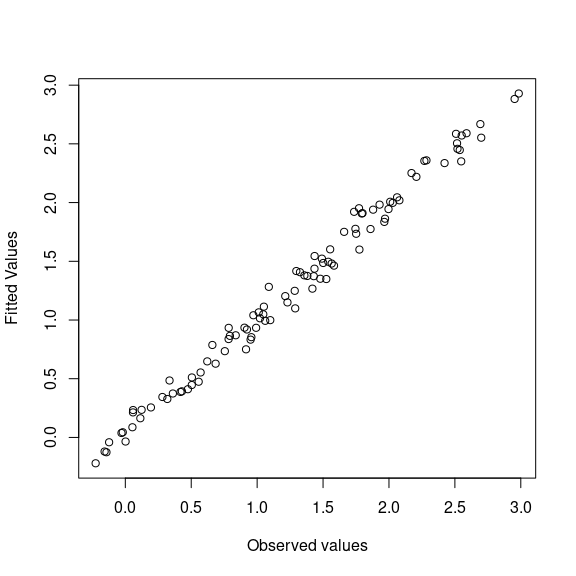

Supplement: Supplementary file 3 [file Data_Sheet_3.zip › plsmselect/vignettes/plsmselect_files/figure-html/fittedvsobserved-1.png]

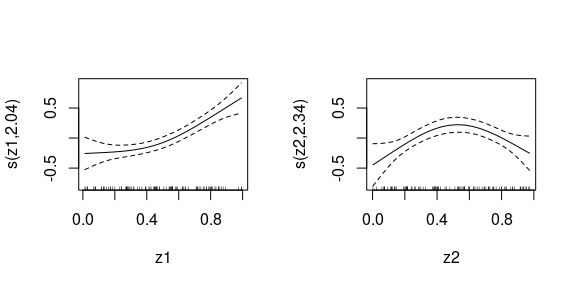

Supplement: Supplementary file 3 [file Data_Sheet_3.zip › plsmselect/vignettes/plsmselect_files/figure-html/unnamed-chunk-7-1.png]

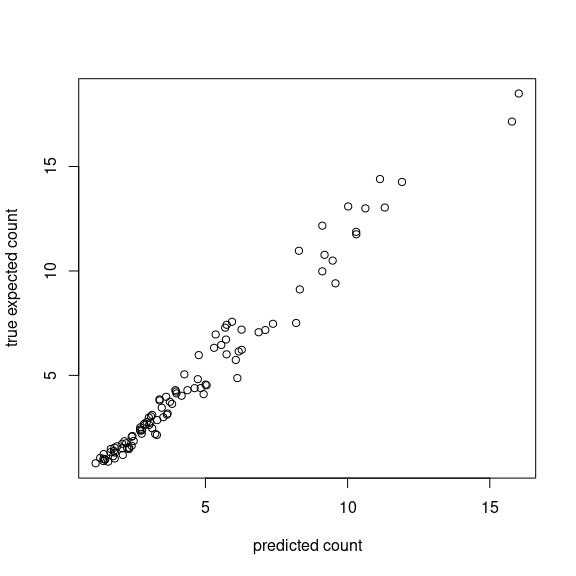

Supplement: Supplementary file 3 [file Data_Sheet_3.zip › plsmselect/vignettes/plsmselect_files/figure-html/unnamed-chunk-8-1.png]

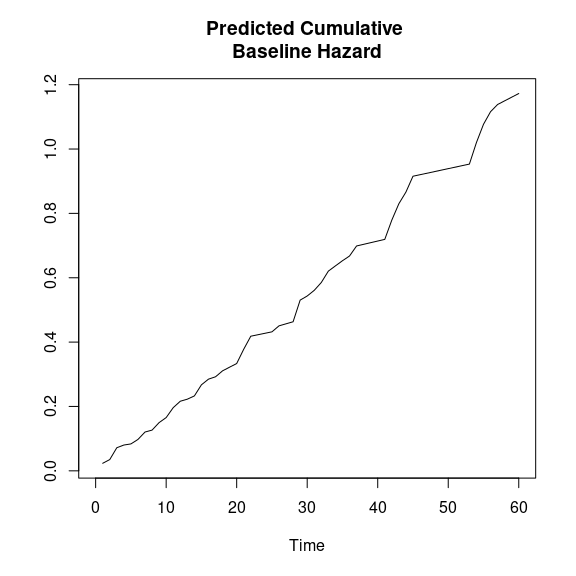

Supplement: Supplementary file 3 [file Data_Sheet_3.zip › plsmselect/vignettes/plsmselect_files/figure-html/coxdiagnos-1.png]

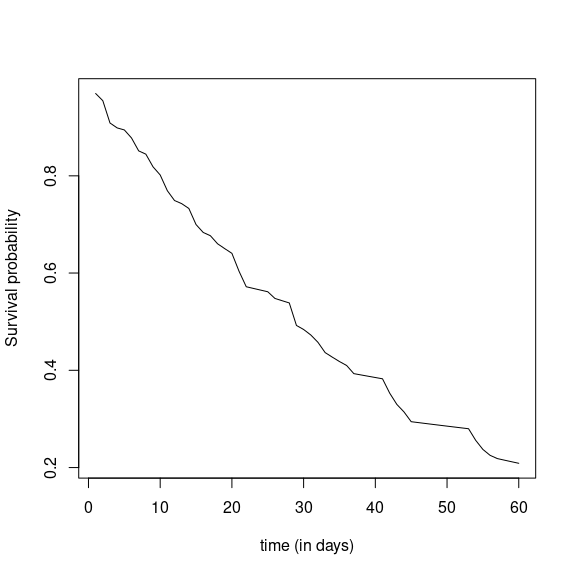

Supplement: Supplementary file 3 [file Data_Sheet_3.zip › plsmselect/vignettes/plsmselect_files/figure-html/coxpredict-1.png]
